# Vertical Flux of Microplastics in the Deep Subtropical Pacific Ocean: Moored Sediment-Trap Observations within the Kuroshio Extension Recirculation Gyre

**DOI:** 10.1021/acs.est.4c02212

**Published:** 2024-08-26

**Authors:** Takahito Ikenoue, Ryota Nakajima, Satoshi Osafune, Eko Siswanto, Makio C. Honda

**Affiliations:** Research Institute for Global Change, Japan Agency for Marine-Earth Science and Technology (JAMSTEC), 2-15 Natsushima-cho, Yokosuka 237-0061, Japan

**Keywords:** microplastic mass flux, sinking particle, marine snow, seasonal variation, biological pump, western North Pacific Ocean

## Abstract

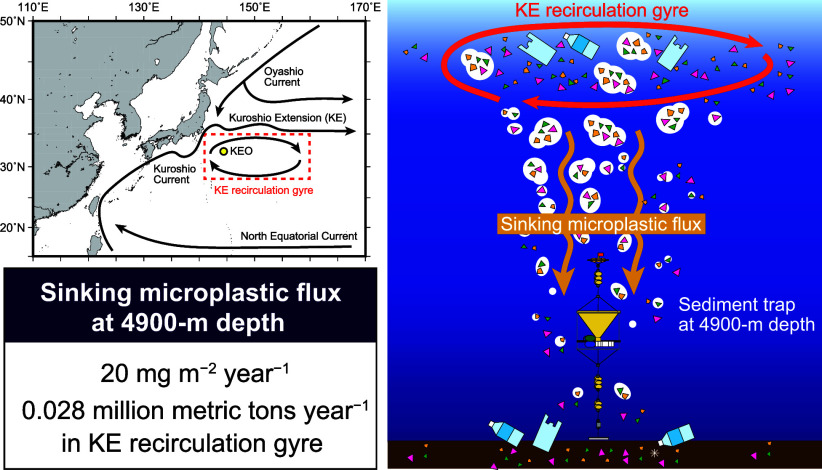

The Kuroshio Extension recirculation gyre in the western North Pacific is an accumulation site of plastic litter transported by the Kuroshio Current. A sediment trap was moored at a depth of 4900 m at Station KEO within the Kuroshio Extension recirculation gyre, and the vertical flux of microplastics in sinking particles of size <1 mm was observed. Forty-one sediment-trap samples collected from July 1, 2014, to October 2, 2016, were analyzed with a micro-Fourier transform infrared spectrometer and microplastics were detected in all samples. Seventeen polymer types were identified, and 90% of the microplastics were less than 100 μm in size. Microplastic sinking was driven by the action of the biological pump, which was in turn driven by seasonal variations in solar radiation and increased surface primary production typical of the spring season. Microplastic mass flux varied from 4.5 × 10^–3^ to 0.38 mg m^–2^ day^–1^ during the sampling period, with a mean and standard deviation of 0.054 ± 0.075 mg m^–2^ day^–1^. Extrapolating the annual microplastic mass flux at Station KEO to the entire Kuroshio Extension recirculation gyre, it is estimated that 0.028 million metric tons of microplastics are transported annually to 4900 m depth in this area.

## Abstract 2

Few studies have examined a long-term time series of microplastic flux in the ocean. This study reports the mass and characteristics of sinking microplastics in the deep North Pacific Ocean.

## 1 Introduction

Marine plastic pollution has spread throughout the world’s oceans,^1,2^ with widespread contamination reported even in the sparsely populated Pacific Arctic Ocean and Southern Ocean.^1,3−5^ Degradation by ultraviolet light, biological factors, and physical fragmentation by waves result in plastic litter that is fragmented into microplastics (MPs), which are defined as plastic pieces of size 5 mm or smaller.^6^ MPs readily adsorb persistent organic pollutants,^7^ and the accumulation of contaminants in organisms via MPs has been reported to extend into deep waters.^8−10^ Therefore, MPs, which do not decompose or degrade in the ocean, can cause serious ocean pollution and have a significant impact on biogeochemical cycles as carriers of various substances.^11^

Considering the 1–3-year residence time of plastic debris in the ocean surface layer, the amount of plastic debris floating on the ocean surface is much smaller than the amount of plastic debris entering the ocean, and the water column and seafloor are considered to be major reservoirs of plastic debris.^2,12−15^ Indeed, large amounts of MPs have been found in deep-sea sediments,^1,16^ and records from sediment cores indicate that the number of plastic particles sinking to the seafloor has continued to increase from the 1950s to the present as the plastics industry has continued to grow.^17,18^ The incorporation of MPs into biogenic aggregates called marine snow (composed of planktonic remains, detritus, fecal pellets, and mucus secreted by algae and bacteria) and the subsequent rapid sinking of marine snow have been suggested as important mechanisms for transporting MPs from the ocean surface to the deep sea.^19^ However, very few studies have estimated the transport of MPs by marine snow to the deep open ocean; those that have were carried out only in the North Atlantic and have relied on drifting sediment traps and moored long-term time-series sediment traps.^20,21^

**Figure 1:**
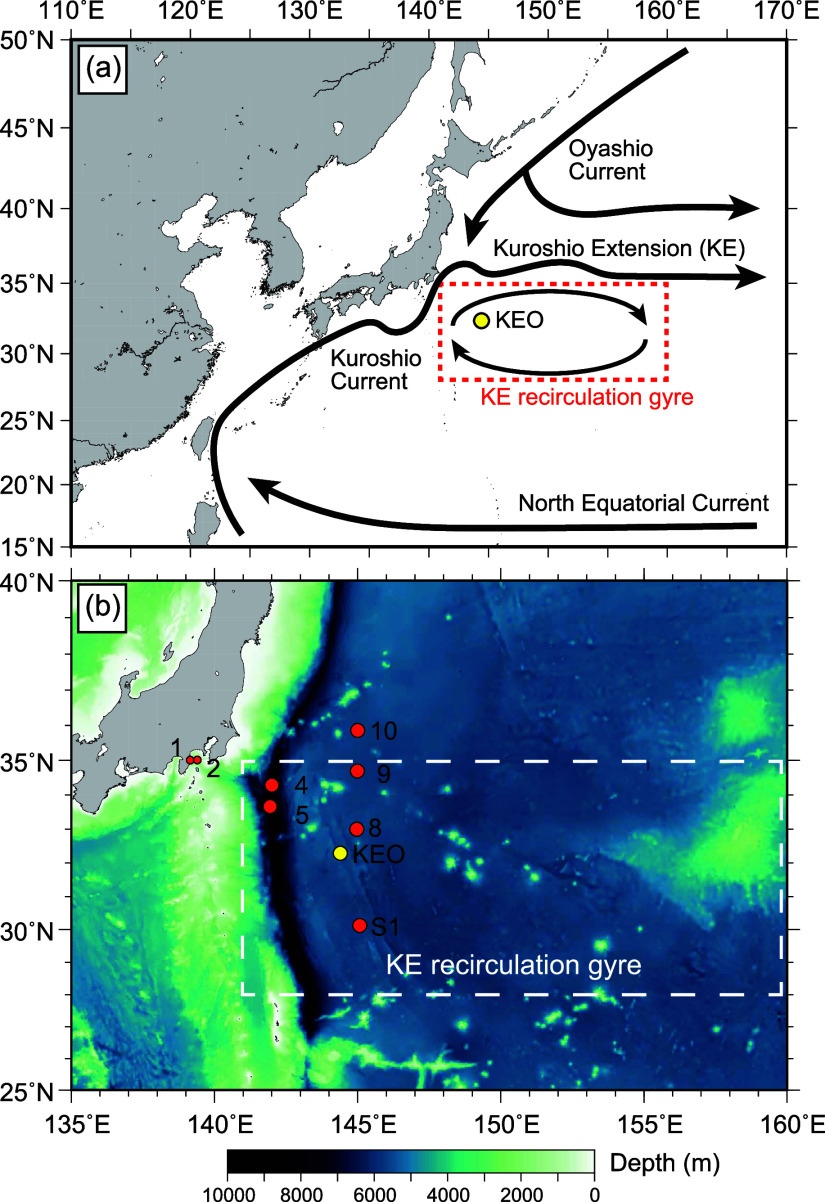
Map of the study area. (a) Schematic general surface circulation in the western North Pacific Ocean. The yellow circle represents the location of the sediment trap mooring at Stn. KEO. Black arrows represent surface currents. Dashed square indicates the extent of the Kuroshio Extension recirculation gyre as defined by Kitamura.^27^ (b) Seafloor map of the area around Stn. KEO. The yellow circle represents the location of Stn. KEO. Orange circles indicate sampling points from previous studies; Stns. 1, 2, 4, 5, 8, 9, and 10 are sediment sampling sites used by Tsuchiya,^30^ and Stn. S1 is the sediment trap mooring site used by Honda.^41^

In Asia, there is a large amount of plastic debris discarded into the environment, and the majority of the world’s marine plastic debris discharged into the ocean via rivers originates from East and Southeast Asia.^22,23^ This plastic debris is transported northward on the Kuroshio Current (KC) to the waters around Japan,^24^ where the concentration of MPs floating on the ocean surface is 27 times higher than the global average,^25^ with large amounts of plastic bags and other debris found on the seafloor at depths of 6500 m.^26^ When the KC merges with the Oyashio Current, it changes its course eastward from off the Boso Peninsula to become the Kuroshio Extension (KE), and the Kuroshio Extension recirculation gyre (KERG), where eddies frequently occur, exists to the south of the KE (Figures 1 and S1).^27^ The KERG is considered an accumulation site of plastic debris carried by the KC,^28^ and large amounts of MPs and plastic debris have been found on the seafloor beneath the KERG.^29,30^

Here we present the first study of the vertical transport of MPs into the deep ocean beneath the KERG off the coast of Japan. We collected sinking marine particles using a long-term time-series sediment trap and quantified the number, mass, and carbon content of MPs in the sinking particles. Using these data to quantify the extent to which MPs advected offshore of Japan via the KC are transported from the surface to the deep sea could contribute greatly to our understanding of plastics that are currently unaccounted for.

## 2 Materials and Methods

### 2.1 Field Sampling

A time-series sediment trap (Mark VII-21; McLane, East Falmouth, MA) with 21 sampling bottles was installed and used to collect sinking particles at a depth of approximately 4900 m (1000 m above the seafloor) at Station (Stn.) KEO (32°22′N, 144°25′E; depth 5900 m) located in the KERG (Figure 1). The Kuroshio Extension Observatory (KEO) time-series station was established in 2004 by Pacific Marine Environmental Laboratory (PMEL) of National Oceanic and Atmospheric Administration (NOAA) with the deployment of a surface mooring (https://www.pmel.noaa.gov/ocs/data/disdel/). The sampling bottles were made of high-density polyethylene and had a capacity of 250 mL. The sediment-trap body consisted of a polyethylene cone and a polycarbonate baffle. Sampling intervals (sampling period per sampling bottle) ranged from 18 to 21 days. In this study, chemical and MP analyses were performed on the <1 mm fractions of 41 sediment-trap samples collected from 1 July 2014 to 2 October 2016. The <1 mm fraction of the sediment-trap samples were divided into 10 equal 50 mL polypropylene cylinders for each analysis using a wet sample divider (WSD-10; McLane) and prefiltered neutralized formalin seawater (the same as that in the sampling bottles). Details of the field sampling and acquisition of hydrography data around station KEO are described in Note S1.

### 2.2 Chemical Analysis

One of the 1/10th-split sediment-trap samples was pretreated (filtered, dried, weighed for total mass flux, and crushed) in a land-based laboratory and then measured for organic carbon by using an elemental analyzer (2400 CHN/O; PerkinElmer, Waltham, MA). The details of the procedure were as previously described by Honda.^31,32^ Total mass flux data and chemical composition data are available online from the KEO Sediment Trap Database (https://www.jamstec.go.jp/egcr/e/oal/oceansites_keo/index.html#data). Some have also been published by Honda.^32^

### 2.3 MP Analysis

In the land-based laboratory, pretreatment procedures were performed on an open clean bench whenever possible, wearing nitrile gloves and cotton lab coats to minimize contamination by external MPs. Milli-Q water was used to clean laboratory instruments, and unused instruments were always covered with aluminum foil. Washing bottles, pipet tips, centrifuge tubes, and glassware, etc. used for pretreatment were washed with Milli-Q water and dried beforehand. Hydrogen peroxide and sodium iodide used in this study were prefiltered through 10-μm stainless-steel filters that were washed and dried in an ultrasonic cleaner.

One of the 1/10th-split sediment-trap samples was purified for 2–7 days with 5–200 mL of 30% hydrogen peroxide, depending on sample volume, for analysis of MPs. The purified sample was collected on a stainless-steel mesh filter (diameter 13 mm) with a pore size of 10 μm to remove the hydrogen peroxide and washed with pure water. After purification, the sample was collected in a 50 mL polypropene (PP) centrifuge tube with 5.3-M sodium iodide solution and centrifuged (2500 rpm for 3 min) to separate contaminants and plastic particles by specific gravity. After centrifugation, a micropipette (pipet tip made of PP) was used repeatedly and 25 mL of supernatant was collected in a glass beaker. The sodium iodide solution was replenished, and the above operation of specific gravity separation of MPs and collection of supernatants was repeated three times, and a total of 75 mL of supernatant was collected. To wash away sodium iodide, the collected supernatant was washed with distilled water on a nylon mesh with a mesh size of 10 μm. The sample was then transferred into a 50 mL glass bottle with distilled water and diluted with distilled water to a volume of 50 mL for use in MP analysis. From the well-mixed sample, 0.5 to 5.0 mL was aliquoted depending on the concentration. The aliquoted sample was collected on a 10-μm stainless-steel mesh filter (diameter 13 mm) using a glass vacuum filter holder with an effective filtration area of 0.13 cm^2^ (KGS-04; Advantec, Tokyo, Japan). Samples on the filter were measured with a micro–Fourier transform infrared spectrometer (μFT-IR) (AIM-9000; Shimadzu, Kyoto, Japan). The size limit of MPs that can be identified by μFT-IR is 10 μm. Therefore, MPs larger than 20 μm in size were targeted for counting in this study.

All particles on the entire filter were tentatively identified by the reflection method and point coordinates were set for all potential plastic particles. Spectra were then acquired for each point coordinate on the entire filter by transmission analysis (aperture size, 20 × 20 μm; integration frequency, 3; spectral range, 700–4000 cm^–1^). Because the infrared light cannot transmit through the stainless-steel mesh wire (thickness 20 μm), we confirmed preliminarily that none of the candidate plastic particles were entirely occluded by the wire. The polymer type was identified from the obtained spectra using the polymer spectra libraries of the Shimadzu Standard Library and UV-Damaged Plastics Library (Shimadzu). The polymer types were finally determined by the spectra, with a hit index of around 60 as the borderline for acceptance. The number of particles on the filter that were identified as MPs was denoted as *N*_filter_.

### 2.4 Calculation of MP Mass

To estimate the mass of plastic particles, fragment MPs were assumed to be ellipsoids, and the volume of MPs (*V*) was calculated using the following equation based on the ellipsoid volume model.^33^
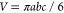
1where *a* is the major axis (the maximum Ferret’s diameter), *b* is the minor axis (the longest axis perpendicular to the major axis), and *c* is the intermediate axis. The major and minor axes of MPs were measured from visible-light images by using LabSolutions IR software (Shimadzu). Aspect ratio, an index of particle shape, was defined as the ratio of short-axis length to major-axis length (0 < aspect ratio ≤ 1). The median aspect ratio was calculated based on the aspect ratios of all fragment-shaped particles (*n* = 362) among the identified MPs, excluding fiber-shaped MPs. The intermediate axis of each MP particle was estimated by multiplying the short axis by the median aspect ratio of all MPs.^34^

The volume of fiber-shaped MPs (*V*) was assumed to be cylindrical with a void fraction of 40%^35^ and calculated as follows
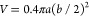
2

The mass of MPs (*M*_MP_) was calculated from the volume of each particle and the density of the polymer (ρ) as follows
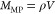
3

The densities (ρ) of the specific polymers used in the calculations are listed in Table S1.

Polymers with a theoretical density of ρ < 1.025 g cm^–3^ were classified as buoyant polymers, and those with ρ > 1.025 g cm^–3^ were classified as dense polymers.

The carbon mass of MPs (*C*_MP_) was determined by using the percentage of carbon in the chemical formula of the polymer (*C*%) (Table S1):
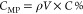
4

### 2.5 Blank and Recovery Test

Three potential MP contamination types were considered for the quantitative evaluation of MP amounts in sediment-trap samples.

Type 1: MP contamination from the prefiltered neutralized formalin seawater added to the sampling bottles (250 mL, polyethylene) prior to deployment of the sediment trap and MPs generated from the sampling bottle during each mooring period (approximately 1 year).

Type 2: MP contamination from the neutralized formalin deep-seawater (about 200 mL) used to sieve the sample through a 1 mm nylon mesh and divide it into 10 portions.

Type 3: MP contamination during the series of procedures starting from purification treatment of the 1/10th-split sample to μFT-IR measurement.

Two blanks (A and B) were prepared to quantify the amounts of the three contamination types; Blank A corresponds to types 1–3 and Blank B to Type 3, details of which are shown in Table S2. Both blanks A and B were fixed at a volume of 50 mL by using the same procedure as the pretreatment for MP analysis of sediment-trap samples. Each blank was then filtered through a stainless-steel filter with a pore size of 10 μm, and all MPs on the filter were measured by using μFT-IR. The number of MPs in the filter samples for Blank A and Blank B are denoted *N*_A_ and *N*_B_, respectively; *N*_B_ was not necessary for the MP flux calculations described below, but was measured to determine the difference between contamination by formalin seawater (contamination types 1 and 2) and contamination that occurred during the sample analysis process (Type 3). Processes not included in types 1–3, such as filling the sediment-trap sampling bottles with seawater and dividing the original sample into tenths, had already been performed before the MP analysis in this study was planned^32^ and were not performed on an open clean-bench environment. Therefore, contamination of MPs from the atmosphere could have occurred during these steps. In addition, the possibility of fiber contamination by researchers has been noted.^35^ Therefore, we chose to exclude fiber MPs from the analysis and only discuss the abundance, mass, and carbon fluxes of fragment MPs to minimize contamination effects, while taking into account the contamination types described above.

To test the possibility that MPs were lost during the procedures described in Section 2.3, MPs recovery was examined. Five recovery tests were conducted and a mean of 94.6% of MPs were recovered, indicating that the analytical procedure used in this study was valid. See Note S2 and Table S3 for more details.

### 2.6 Flux Calculation

Before calculating the MP abundance flux (pieces m^–2^ day^–1^), the blank-corrected MP number (*N*) per 1/10th-split sediment-trap sample (containing about 50 mL of prefiltered neutralized formalin seawater) was calculated as follows:
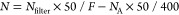
5where *N*_filter_ is the number of MPs in the filter sample, *F* is the volume of sample prepared by micropipetting for μFT-IR analysis, and *N*_A_ is the number of MPs in the Blank A filter sample.

The MP abundance flux (pieces m^–2^ day^–1^) was calculated from MP count data using the following formula:
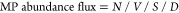
6where *N* is the blank-corrected number of MPs per 1/10th-split sediment-trap sample, *V* is the aliquot size (1/10), *S* is the aperture area of the sediment trap (0.5 m^2^), and *D* is the sampling interval (days).

The MP mass flux (mg m^–2^ day^–1^) was calculated using the following formula:
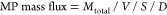
7where *M*_total_ is sum of the individual *M*_MP_ per 1/10th-split sediment-trap sample.

The MP carbon flux (mg-C m^–2^ day^–1^) was calculated using the following formula:
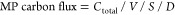
8where *C*_total_ is sum of the individual *C*_MP_ per 1/10th-split sediment-trap sample.

## 3 Results

### 3.1 Hydrography and Temporal Variations of Sinking Particle Fluxes

**Figure 2:**
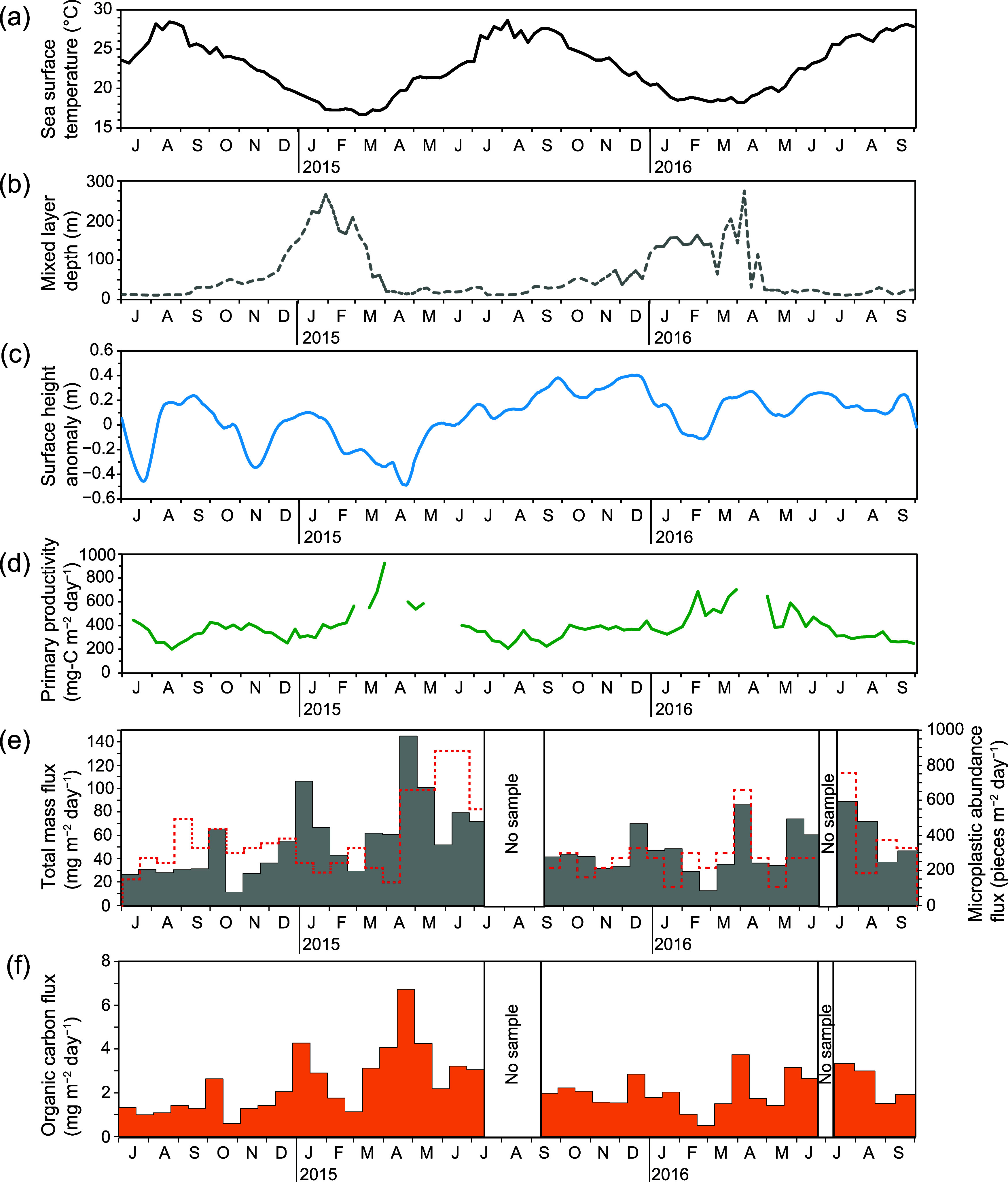
Seasonal variations at Stn. KEO of (a) sea surface temperature, (b) mixed layer depth, (c) sea surface height anomaly, (d) primary productivity (mg-C m^–2^ day^–1^), (e) sinking particle total mass flux, and (f) sinking particle organic carbon flux. The red dashed line in (e) indicates microplastic abundance flux.

Sea surface temperature (SST) around Stn. KEO varied from about 16.7 to 28.6 °C during the sampling period, with warming being observed above 25 °C from July to October and cooling below 19 °C from January to April (Table S4 and Figure 2a). Mixed layer depth (MLD) varied between 11 and 274 m and reaching depths greater than 100 m from January to March due to vertical mixing associated with the winter decline in SST (Table S4 and Figure 2b). Sea surface height anomaly (SSHA) varied from −0.49 to 0.40 m, with values below–0.2 m observed in July and November in 2014 and in February to May in 2015 (Table S5 and Figure 2c); in 2016, negative values were only observed in February. Primary productivity varied from 201 to 926 mg-C m^–2^ day^–1^, with a mean and standard deviation of 396 ± 130 mg-C m^–2^ day^–1^, and exceeded 500 mg-C m^–2^ day^–1^ from February to May (late winter to spring) (Table S6 and Figure 2d). The distribution of annual mean absolute dynamic topography around Stn. KEO in 2014, 2015, and 2016 indicates that the station was located within the KERG during the sampling period (Figure S1).

Total mass flux at Stn. KEO varied from 11.5 to 144.8 mg m^–2^ day^–1^ during the sampling period (52.0 ± 26.8 mg m^–2^ day^–1^) (Table S7 and Figure 2e). Organic carbon flux varied between 0.51 and 6.7 mg-C m^–2^ day^–1^ during the sampling period (2.3 ± 1.2 mg-C m^–2^ day^–1^) (Table S7 and Figure 2f). Total mass flux and organic carbon flux increased from March to June 2015 (late winter to late spring), from March to August 2016 (late winter to summer), in October 2014, and from late December 2014 to January 2015. Most of these data have already been reported by Honda.^32^

### 3.2 Polymer Types and Size Distributions of MPs Collected by the Sediment Trap

A total of 17 polymer types were identified at Stn. KEO from 1 July 2014 to 2 October 2016. The number of MPs counted in each filter sample ranged from 5 to 20 pieces, for a total of 405 pieces (Table S8). Of these, 362 were fragments and 43 were fibers. The length of the major axis of the detected fragment MPs ranged from 20 to 480 μm (66 ± 53 μm) (Table S7). MPs smaller than 100 μm in size accounted for 90% of all fragment MPs, and the 30–60 μm size range accounted for 75% (Table S8 and Figure S2). The median aspect ratio of fragment MPs was 0.75 (0.75 ± 0.20) (Table S7).

### 3.3 Temporal Variations of MP Abundance, Mass, and Carbon Fluxes

Two PET fragments were detected in Blank A, and no MPs were detected in Blank B (Table S8). This suggests the possibility of MP contamination from the seawater used to fill the sampling bottles and used for sample splitting, rather than from the sample pretreatment and analysis processes. Because the seawater was prefiltered and the storage container was made of PE, it is likely that the detected PET was not originally present in the seawater and instead entered the samples during the process of filling the container with seawater. Two PET fragments in Blank A is the equivalent of 0.25 MPs 1/10th-split sediment-trap sample. Therefore, the amount of MP contamination from this contamination route was negligible, with essentially no change in the number of MPs in the samples after blank correction.

**Figure 3:**
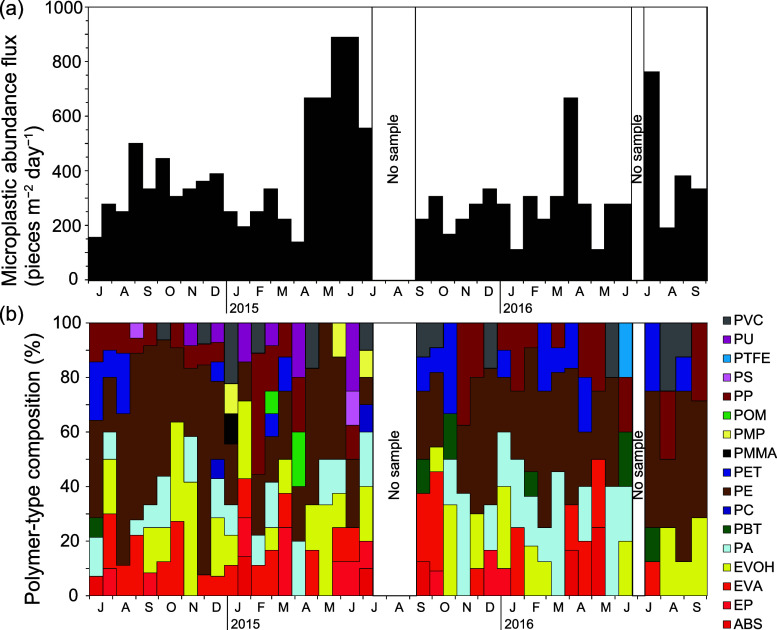
Time-series of microplastic abundance fluxes at Stn. KEO. (a) Microplastic abundance fluxes. (b) Polymer-type composition by number of particles. Polymer-type abbreviations are as shown in Table S1.

After blank correction, MP abundance flux, excluding fiber MPs, varied from 111 to 889 pieces m^–2^ day^–1^ during the sampling period (352 ± 194 pieces m^–2^ day^–1^) (Tables S7 and S9, and Figure 3a). Variations in MP abundance flux were observed from year to year and month to month, with fluxes more than double the overall mean from April to June 2015, from March to April 2016, and in July 2016 (Figure 3a). Polymer types that accounted for more than 5% of mean MP abundance flux during the sampling period were PE (37.4%), EVOH (12.1%), PA (10.0%), EVA (9.6%), PP (7.7%), and PET (5.9%), accounting for 82.7% of the total MP abundance flux (Table S10 and Figure 3b). MPs of <100 μm size accounted for 90.1% of the total MP abundance and MPs of >100 μm size for 9.9% (Table S11 and Figure S3). Buoyant and dense polymers accounted for 56 and 44% of the total MP abundance, respectively. The Shannon diversity index (*H*′) (using 2 as the base of the logarithm) calculated based on the number of polymers in all samples during the sampling period was 3.0 (Table S7).

**Figure 4:**
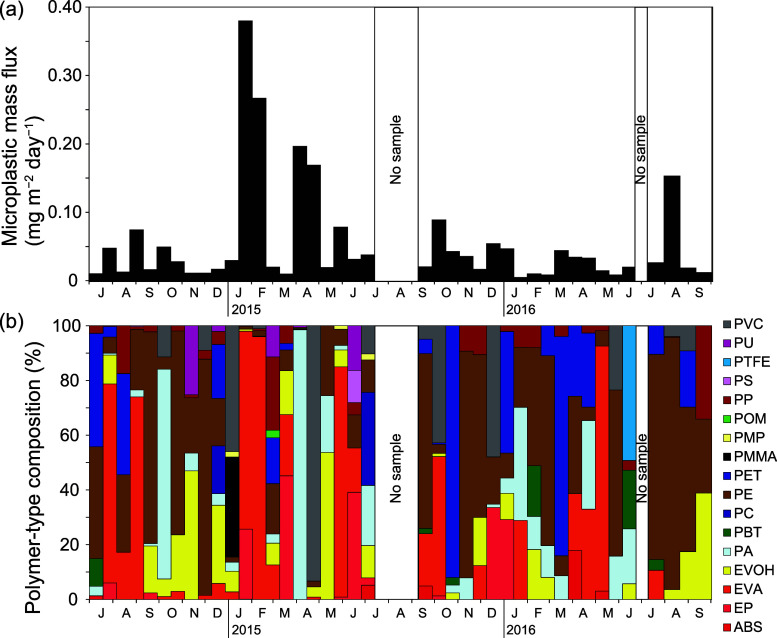
Time-series of microplastic mass fluxes at Stn. KEO. (a) Microplastic mass fluxes. (b) Polymer-type composition by mass. Polymer-type abbreviations are as shown in Table S1.

MP mass fluxes varied from 4.5 × 10^–3^ to 0.38 mg m^–2^ day^–1^ during the sampling period (0.054 ± 0.075 mg m^–2^ day^–1^) (Tables S7 and S12, and Figure 4a). Fluxes more than double the mean were observed in January, February, and April 2015 and in August 2016 (Figure 4a). Polymer types that accounted for more than 5% of the mean MP mass flux during the sampling period were EVA (35.3%), PE (18.7%), PA (12.6%), PVC (11.6%), EP (6.8%), and PET (6.3%), representing 91.2% of the total MP mass flux (Table S10 and Figure 4b). MPs with sizes <100 μm accounted for 24.1% of the total MP mass flux and MPs with sizes >100 μm accounted for 75.9% (Table S11 and Figure S4).

MP carbon flux varied from 3.2 × 10^–3^ to 0.24 mg m^–2^ day^–1^ during the sampling period (0.035 ± 0.048 mg m^–2^ day^–1^). Fluxes more than double the mean were observed in January, February, and April 2015 and in August 2016, as with MP mass fluxes (Tables S7 and S13, and Figure S5a). Polymer types that accounted for more than 5% of the mean MP carbon flux during the sampling period were EVA (34.2%), PE (24.6%), PA (12.3%), PVC (6.9%), EP (6.7%), and PET (6.0%), representing 90.8% of the total MP carbon flux (Table S10 and Figure S5b).

## 4 Discussion

### 4.1 Relationship between MP Abundance Flux and Sinking Particle Flux

Primary production around Stn. KEO increased from February to May in both 2015 and 2016, following the supply of nutrients to the subsurface by winter vertical mixing and subsequent spring warming (Figure 2d). Total mass flux and organic carbon flux at a depth of 4900 m at Stn. KEO increased from March to June in both years (Figure 2d,e), which would reflect this increase in primary production if we assume a sedimentation rate of 100–200 m day^–1^.^32^ In 2016, an increase in sinking particle flux was also observed from July to August, possibly due to the fact that the period of high primary production in 2016 was a month longer than in 2015. The decreases in SSHA in July and November 2014 reflect the passage of cyclonic eddies at Stn. KEO, and nutrient upwellings from the subsurface layer caused by the eddies were observed in July and November 2014.^32^ Therefore, the increase in sinking particle flux in October 2014 and from late December 2014 to January 2015 can be attributed to increased primary production in the subsurface due to the cyclonic eddies.^32^ Although cyclonic eddies also passed through Stn. KEO from February to May 2015 and in February 2016 (Figure 2c), these eddies did not contribute to nutrient supply during these periods because the surface layer was already supplied with nutrients from the subsurface due to wintertime vertical mixing caused by sea surface cooling at Stn. KEO.^32^ Thus, the two main factors for the increase in sinking particles from July 2014 to July 2015 at Stn. KEO were the typical springtime increase in surface primary production associated with seasonal variations in solar radiation and an increase in subsurface primary production due to cyclonic eddies. The increase in sinking particles from September 2015 to September 2016 was primarily due to an increase in surface primary production associated with seasonal variations in solar radiation.

Correlations between MP abundance flux and total mass flux were all weakly positive, with *r* = 0.49 (*p* = 0.002) for the entire sampling period, *r* = 0.42 (*p* = 0.057) from July 2014 to July 2015 (the first year), and *r* = 0.61 (*p* = 0.004) from September 2015 to September 2016 (the second year) (Figure 2e). The low *r* value in the first year and higher *r* value in the second year could indicate that the springtime increase in surface primary production contributed more to the sinking of MPs to the deep sea than did the increase in subsurface primary production due to cyclonic eddies. The sinking of MPs at Stn. KEO could have been enhanced by the formation of algal and bacterial biofilms on MPs floating at the ocean surface during spikes in surface primary production, or by the entrainment of MPs in aggregates^36^ or in the fecal pellets of zooplankton preying on phytoplankton near the surface.^37^ In fact, more MP deposition has been recorded in coastal marine sediments in years of high phytoplankton production,^18^ suggesting that aggregate formation of sinking particles is an important mechanism for transporting MPs from the surface, even in the open ocean. However, this mechanism requires that both MPs and aggregates be present at the ocean surface at the same time. The reason the peaks of MP abundance flux and total mass flux did not always coincide at our study site (and the correlation between them was low) is probably because MPs in the ocean surface layer are unevenly distributed depending on ocean conditions and the timing of MP discharge from land. Although cyclonic eddies can accumulate suspended surface MPs while incorporating surface water masses from outside the eddy,^38^ the timing of the cyclonic eddy’s passage did not match the timing of the increase in MP abundance flux in this study. Because cyclonic eddies create upwelling of subsurface water masses inside the eddy,^38^ they might not contribute to the sinking of MPs near Stn. KEO, but their role in the basin-scale accumulation and sinking of MPs is unknown. Recently, a carbon export mechanism called “physical injection pumps” has been attracting attention in the field of biogeochemistry, in which suspended particles near the surface, which are basically nonsinking, are transported to the ocean interior by the vertical flow of seawater and by the vertical migration of zooplankton.^39^ MP transport into the ocean interior by physical injection pumps should be studied in detail in the future.

### 4.2 Characteristics of Sinking MPs

Plastic debris and MPs are likely to accumulate in the surface layer of the KERG and sink to the abyssal plain.^29,30^ Data for MPs in western North Pacific sediments,^30^ excluding fiber MPs and recalculated with aspect ratios as the ratios of the minor to major axis, are shown in Table S14, and the mean size, aspect ratio, and *H*′ are shown in Table S15. In the abyssal sediments of the western North Pacific, small MPs are more prevalent in the open ocean than near the coasts, and the aspect ratio is close to 1 (Table S15).^30^ Therefore, small MPs with aspect ratios close to 1 are more likely to be transported farther from their sources and over longer distances. At Stn. KEO, MPs of size <100 μm accounted for 90% of MP abundance flux of all fragment MPs at a depth of 4900 m (Figure S2), consistent with the proportion (93%) in sediments of the abyssal plain (Stns. 8–10; Figure 1b) beneath the KERG in the western North Pacific.^30^ The mean aspect ratio of MPs at Stn. KEO was 0.75, which is close to 1, and is similar to the aspect ratio of MPs in abyssal plain sediments (0.67–0.68). Furthermore, at a depth of 2000 m at Stn. Kiel 276 in the Northeast Atlantic subtropical gyre, the percentage of MPs < 100 μm in MP abundance flux was 74%,^21^ which is less than that at a depth of 4900 m at Stn. KEO. This suggests that smaller MPs are more likely to be transported with aggregates to deep water. Because Stn. KEO is in the open ocean and the sediment trap was moored approximately 1000 m above the seafloor, suspended MPs originating from the seafloor are unlikely. Therefore, the sinking MPs collected at Stn. KEO were likely transported over long distances from their source regions by the KC and KE.

In western North Pacific sediments, the *H*′ of polymer type decreases from the coast to the open ocean.^30^ In the abyssal plain near Stn. KEO, *H*′ ranges from 0.5 to 0.8, and PE accounts for more than 75% of the polymers (Tables S14 and S15). However, in contrast to the sediments, the *H*′ of sinking MPs at Stn. KEO was 3.0, which is higher than that of coast sediments, and the polymer type composition was closer to that of the coastal sediments than to that of the abyssal plain. The surface 0–1 cm layer of abyssal plain sediments has a depositional age of 463–614 years (Table S15),^30^ accounting for the entire time period of MP deposition from the 1950s, when plastic industrialization began, to the present (Table S15). Therefore, the abyssal plain sediments reflect the polymer type composition of MPs accumulated since the 1950s, whereas the sinking MPs collected in the present study reflect the composition of MPs in the marine surface layer at the time of collection (2014–2016). The reason the size and aspect ratio of sinking MPs at station KEO show polymer type compositions resembling those of coastal sediments while also having characteristics that resemble open-ocean abyssal sediments is probably because the surface 0–1 cm layer of coastal sediments corresponds to only 3–4 years of MP deposition (Table S15).^30^ The percentage of buoyant polymers in sinking particles and in marine sediments is higher than that of dense polymers (Table S15), probably due to the higher production and disposal of buoyant polymers such as PE and PP on land at present.

Despite being located in the open ocean, Stn. KEO has a relatively high mean lithogenic material flux of sinking particles of 18.9 mg m^–2^ day^–1^,^40^ 2–3-fold higher than at Stn. S1 (7.1 mg m^–2^ day^–1^), located south of Stn. KEO and also within the KERG (Figure 1).^41^ The source of lithogenic material at Stn. KEO is not yet known, but as no earthquakes occurred during the observation period, it is possible that lithogenic material is regularly transported to Stn. KEO by the KE. Therefore, it might be possible to identify one of the sources of sinking MPs in this area by investigating of the origin of the lithogenic material in the future.

### 4.3 Impact of MPs Sinking to the Deep Sea

At station KEO, MPs with sizes >100 μm accounted for 75.9% of the mass flux, despite only 9.9% of the abundance flux (Table S11, Figures S3 and S4). This indicates that the magnitude of the MPs mass and carbon fluxes depends primarily on size and volume, rather than on the number of MPs; the high contribution of EVA in the MPs mass and carbon fluxes was also due to the inclusion of large EVAs with sizes >300 μm (Table S8). The mean MP abundance flux at Stn. KEO was 84% of that at station Kiel 276 in the Northeast Atlantic subtropical gyre, which is comparable. On the other hand, MP mass flux and MP carbon flux were much lower than those at Stn. Kiel 276, at 30 and 25%, respectively (Table S7), indicating that the sediment-trap sample at Stn. KEO contained more MPs of smaller size than those collected at Stn. Kiel 276. Usually, particulate organic carbon fluxes in sinking particles are attenuated by chemical, biological, and physical effects during gravity-driven sinking in the ocean; MPs are rarely degraded by microorganisms, but since MPs are transported vertically as part of organic matter aggregates, they can be released into the water column when the particulate organic carbon that makes up the aggregates is degraded. Because the sediment trap at Stn. KEO was deployed at a depth 2900 m deeper than that at Stn. Kiel 276 (4900 m vs 2000 m), it is necessary to normalize the MP fluxes to the same water depth to determine which area ultimately has higher MP fluxes. The rate of attenuation of particulate organic carbon flux is expressed by an empirical equation called the Martin curve, and the difference in the rate of vertical change between ocean regions is expressed by the parameter Martin’s *b* (exponent *b* of Martin curve: POC_(*z*)_ = POC_(100)_ × (*z*/100)^−*b*^, where POC_(*z*)_ and POC_(100)_ represent the POC flux at water depths of *z* m and 100 m, respectively.).^42^ Assuming that MPs in sinking particles are released into the water column at the same rate as the degradation of particulate organic carbon, and applying the subtropical Martin’s *b* value of 0.3 to 0.5,^43^ MP abundance flux at station Kiel 276 attenuates to 269–322 pieces m^–2^ day^–1^ at a water depth of 4900 m. Therefore, MP abundance fluxes in the KERG may be equal to or greater than those in the Northeast Atlantic subtropical gyre.

The MP carbon flux at Stn. KEO accounts for an average of 1.5% of the organic carbon flux of sinking particles (Table S7). When dead carbon depleted of ^14^C, such as plastics, contaminates environmental samples, this can produce an age error during carbon dating. A sample that contains 1% plastic contaminants, for example, will appear to be 80 years older than it actually is, independent of the age of the sample.^44^ Therefore, if seafloor sediments are contaminated by MPs at the same rate as the sinking particles observed at Stn. KEO, a dating error of 120 years would result. This suggests that if the discharge of plastic debris into the environment is not controlled, the accumulation of MPs on the seafloor could affect studies in various fields of Earth science.

The annual MP flux calculated from the mean MP flux during the sampling period at Stn. KEO was 128,480 pieces m^–2^ year^–1^ (352 × 365), accounting for a mass of 20 mg m^–2^ year^–1^ (0.054 × 365, Table S7). The collection efficiency of sediment traps installed at depths greater than 1000 m is almost 100%,^45^ sinking particles collected by sediment traps in deep water (e.g., 4900 m depth) are representative of particles sinking within a radius of several hundred km.^46^ The KERG extends between 28°N and 36°N latitude and 140°E and 160°E longitude and has a total area of about 1.4 million km^2^ (Figure 1).^27^ Assuming that the annual MP flux within the KERG is equal to that of Stn. KEO, 0.18 billion pieces per year of MP are transported into the deep ocean within the KERG, accounting for 0.028 million metric tons (MT) per year in mass. Since estimates of plastic debris entering the global ocean through rivers each year range from 0.41 to 4.0 MT,^23,47^ the mass of MPs accumulated annually in the KERG is equivalent to 0.69 to 6.7% of that amount.

World plastic production, excluding fibers, has increased from 2 MT in 1950 to 381 MT in 2015 (Table S16).^48^ Assuming that MP mass flux at Stn. KEO has increased at the same rate as global plastics production since 1950 and reached 20 mg m^–2^ year^–1^ in 2015, the mass of MPs transported to the deep sea from 1950 to 2015 would be 405 mg m^–2^ or 0.567 MT within the KERG alone (Table S16). The total mass of plastic debris accumulated in the global ocean since the 1960s is estimated to be about 25.3 MT, of which 16.88 MT has sunk into the interior of the open ocean, based on computer simulations.^49^ The estimated amount of sunken MP within the KERG in this study accounts for 3.4% of the plastic debris sunk in the open ocean globally to date. Therefore, even though the KERG covers only 1.8% of the world’s oceans, these data suggest that large amounts of MP are transported to this area by the KC and accumulate at depth.

The mass equivalent of MPs in abyssal sediment in the western North Pacific abyssal plain (including MPs deposited from 1950 to 2019) reported by Tsuchiya,^30^ using the same method as in the present study, is 15–129 mg m^–2^ (Tables S14 and S15), which is 3.7–32% of the estimated MP mass transported to the deep sea by 2015 at Stn. KEO. Fragmentation of sinking particle aggregates in the mesopelagic zone accounts for 49 ± 22% of the sinking particle flux loss.^50^ During descent from 4900 m (the sediment-trap depth) to the seafloor (5900 m), the MP fluxes we observed might be attenuated by the fragmentation of sinking particle aggregates by microbes, predation by zooplankton, and/or horizontal transport before reaching the seafloor. If the aggregates of sinking particles containing MPs are degraded by organisms, individual buoyant polymer MPs would lose their sinking power and become suspended in the water column. In fact, an increase in MPs has been observed at a depth of 2000 m in the North Pacific Subtropical Gyre.^51^ Even after depositing on the seafloor, buoyant polymer MPs can resuspend due to further decomposition of the aggregates.

In the present study, we inferred the transport of MPs by the biological pump from the surface to a depth of 4900 m at station KEO. However, understanding the fate of MPs transported to the deep sea will require a comprehensive investigation of MP distribution in the water column, MP sinking fluxes at multiple depths, and extensive horizontal distribution studies of MPs in abyssal sediment.

## Supporting Information Available

Additional field sampling details; density and chemical formulas of polymer types; list of blanks; recovery rate; hydrography data at Stn. KEO; fluxes of sinking particles and MPs at Stns. KEO and Kiel 276; summary of individual MPs at Stn. KEO; abundance, mass, and carbon fluxes of MPs at Stn. KEO; polymer-type and size composition by abundance, mass, and carbon fluxes of MPs at Stn. KEO; summary and calculated volume and mass of individual MPs in sediment; summary and calculated MP mass in sediment samples; and estimated microplastic mass flux at Stn. KEO (PDF)
