# Supplementary material for: Vertical Flux of Microplastics in the Deep Subtropical Pacific Ocean: Moored Sediment-Trap Observations within the Kuroshio Extension Recirculation Gyre
Source: Environ Sci Technol. 2024 Aug 26;58(36):16121–30. doi: 10.1021/acs.est.4c02212 (PMC11394010; doi:10.1021/acs.est.4c02212)
Supplement: Supplementary file 1 — es4c02212_si_001.pdf [file es4c02212_si_001.pdf]

Supporting Information

for

Vertical flux of microplastics in the deep subtropical Pacific Ocean: moored sediment-trap observations within the Kuroshio Extension recirculation gyre

Takahito Ikenoue[a]*; Ryota Nakajima[a]; Satoshi Osafune[a]; Eko Siswanto[a]; Makio C. Honda[a]

[a] Research Institute for Global Change, Japan Agency for Marine-Earth Science and Technology (JAMSTEC), 2-15 Natsushima-cho, Yokosuka 237-0061, Japan

* Corresponding author: ikenouet@jamstec.go.jp

This PDF file includes:

Note S1 (pages S2–S3), Note S2 (pages S4)
Tables S1 (page S5), S2 (page S6), S3 (page S7), S4 (pages S8–S11), S5 (pages S12–S29), S6 (pages S30–S32), S7 (page S33), S8 (pages S34–S42), S9 (page S43), S10 (page S44), S11 (page S45), S12 (page S46), S13 (page S47), S14 (pages S48–S58), S15 (page S59), and S16 (pages S60–S61)
Figures S1 to S5 (pages S62–S66)
SI References (page S67)

Note S1

Field sampling

A time-series sediment trap (Mark VII-21; McLane, East Falmouth, MA) with 21 sampling bottles was installed and used to collect sinking particles at a depth of approximately 4900 m (1000 m above the seafloor) at Station (Stn.) KEO (32°22′N, 144°25′E; depth 5900 m) located in the KERG (Fig. 1). The Kuroshio Extension Observatory (KEO) time-series station was established in 2004 by Pacific Marine Environmental Laboratory (PMEL) of National Oceanic and Atmospheric Administration (NOAA) with the deployment of a surface mooring (https://www.pmel.noaa.gov/ocs/data/disdel/). The sampling bottles were made of high-density polyethylene and had a capacity of 250 mL. The sediment-trap body consisted of a polyethylene cone and a polycarbonate baffle. The sediment-trap mooring system was first deployed from aboard R/V *Kaiyo* in June 2014, followed by M/V *Bluefin* in September 2015, T/S *Shinsei-maru* in July 2016, and T/S *Hakuho-maru* in November 2017. The openings of the sediment traps were covered until just prior to deployment to prevent contamination by MPs from the atmosphere. Sampling intervals (sampling period per sampling bottle) ranged from 18 to 21 days. Sampling bottles were washed and dried with dilute hydrochloric acid and Milli-Q water. Neutralized formalin deep-sea water (final conc. 10%) was filtered through Gelman membrane filters with a nominal pore size of 0.45 μm and sampling bottles were filled with the pre-filtered neutralized formalin deep-sea water before deployment. The salinity was adjusted to approximately 39 by the addition of NaCl (50 g of NaCl to 10 L of seawater). The initial pH of the solution was about 8.1. After recovery of the sediment traps, the samples were stored in a refrigerator at 4 °C until analysis in a land-based laboratory. In the laboratory, the collected sinking particles were sieved through a 1-mm nylon mesh and any remaining swimmers of size <1 mm were removed with tweezers. The <1-mm fraction of the sediment-trap samples were divided into 10 equal 50-mL polypropylene cylinders for each analysis using a wet sample divider (WSD-10; McLane) and pre-filtered neutralized formalin sea water (the same as that in the sampling bottles). In this study, chemical and MP analyses were performed on the <1-mm fractions of 41 sediment-trap samples collected from 1 July 2014 to 2 October 2016.

Sea surface temperature (SST) (°C) and mixed layer depth (MLD) (m) around KEO (32°N−33°N, 144°E−145°E) were obtained from Copernicus Marine Service (CMEMS) (https://data.marine.copernicus.eu/products, accessed 29 January 2024). Sea surface chlorophyll-*a* concentration (Chl-a) (mg m^{-3}) and contemporaneous SST (°C), photosynthetically active radiation (Einstein m^{-2} d^{-1}), depth of euphotic zone (m), and day lengths were obtained from the Moderate Resolution Imaging Spectroradiometer onboard Satellite Aqua provided by the Ocean Biology Processing Group at the National Aeronautics and Space Administration Goddard Space Flight Center (https://oceancolor.gsfc.nasa.gov, accessed 29 January 2024). Eight-day mean chlorophyll-*a* concentrations were calculated for a 4° × 4° square centered at Stn. KEO. Primary productivity data

(mg-C m^{-2} day^{-1}) estimated using the Vertically Generalized Production Model[1] were obtained from the Ocean Productivity website provided by Oregon State University (http://orca.science.oregonstate.edu/npp_products.php, accessed 29 January 2024). Gridded sea surface height anomaly data (SSHA; m) with a spatial resolution of 0.25° for both longitude and latitude based on multiple satellite altimeters were also obtained from CMEMS to serve as an indicator of the passage of cyclonic eddies around Stn. KEO. Annual mean absolute dynamic topography with the same spatial resolution was derived from sea surface height data obtained from CMEMS to determine the positional relationship between Stn. KEO and the KERG during the sampling period.

Note S2

Recovery test

To test the possibility that MPs were lost during the procedure described in section 2.3 from purification with hydrogen peroxide to dilution with distilled water to a volume of 50 mL for use in MP analysis, the MPs recovery was examined. One hundred pink-colored polyethylene particles of size 150-180 µm (Cospheric Product ID# PNKPMS-1.00) were added to 200 ml of Milli Q water as a standard sample. The recovery test was conducted five times and µFT-IR identification showed that a mean of 94.6% of the MPs were recovered, indicating that the analytical procedure used in this study was valid. The results of the individual recovery tests are summarized in Table S3.

Table S1. Theoretical density and chemical formulas of polymer types

Polymer type	Chemical formula	Carbon portion	Density (g/cm^3)			Polymer category	Reference
			Minimum	Maximum	Mid-range		
Acrylonitrile-butadiene-styrene copolymer (ABS)	$C_8H_8.C_4H_6.C_3H_3N$	85.3	1.02	1.21	1.12	Dense	Omnexus[2]
Epoxy resin (EP)	$C_{21}H_{25}ClO_5$	64.2	1.10	1.50	1.30	Dense	Resin Library[3]
Ethylene-vinylacetate copolymer (EVA)	$C_4H_6O_2.C_2H_4$	63.1	0.92	0.94	0.93	Buoyant	Omnexus[2]
Ethylene-vinylalcohol copolymer (EVOH)	$C_2H_4O.C_2H_4$	66.6	1.10	1.20	1.15	Dense	Omnexus[2]
Polyamide (PA)	$C_{12}H_{22}N_2O_2$	63.7	1.13	1.15	1.14	Dense	Omnexus[2]
Poly (butylene terephthalate) (PBT)	$C_{12}H_{12}O_4$	65.4	1.30	1.40	1.35	Dense	Omnexus[2]
Polycarbonate (PC)	$C_{16}H_{18}O_5$	66.2	1.15	1.52	1.34	Dense	Omnexus[2]
Polyethylene (PE)	C_2H_4	85.6	0.92	0.97	0.94	Buoyant	Omnexus[2]
Ethylene/propylene copolymer (PE/PP)	$C_2H_4.C_3H_6$	85.6	0.86	0.86	0.86	Buoyant	Scientific Polymer Products Inc.[4]
Poly (ethylene terephthalate) (PET)	$C_{10}H_8O_4$	62.5	1.30	1.40	1.35	Dense	Omnexus[2]
Poly (methyl methacrylate) (PMMA)	$C_8H_8.C_4H_6.C_3H_3N$	85.3	1.17	1.20	1.19	Dense	Omnexus[2]
Poly (methyl pentene) (PMP)	C_6H_{12}	85.6	0.84	0.84	0.84	Buoyant	Omnexus[2]
Polyoxymethylene (POM)	CH_2O	40.0	1.41	1.42	1.42	Dense	Omnexus[2]
Polypropylene (PP)	C_3H_6	85.6	0.90	0.91	0.91	Buoyant	Omnexus[2]
Polystyrene (PS)	C_8H_8	92.3	1.04	1.05	1.05	Dense	Omnexus[2]
Polytetrafluoroethylene (PTFE)	C_2F_4	24.0	2.10	2.20	2.15	Dense	Omnexus[2]
Polyurethane (PU)	$C_3H_8N_2O$	40.9	1.11	1.24	1.18	Dense	Ji-Horng Plastic[5]
Poly (vinyl chloride) (PVC)	C_2H_3Cl	38.4	1.15	1.70	1.43	Dense	Omnexus[2]
Vinyl chloride-vinyl acetate copolymers (VC/VA)	$C_2H_3Cl.C_4H_6O_2$	48.5	1.33	1.37	1.35	Dense	Scientific Polymer Products Inc.[4]

Table S2. List of blanks

Blanks	Remarks
Blank A	400 mL of prefiltered neutralized formalin sea water collected at a depth of 2000 m at Stn. K2 (47°N, 160°E) on 3 December 2020 during cruise MR20-E02 and analyzed after 869 days of cold storage in a polyethylene container. This allowed us to reproduce the MPs generated by the sampling bottle during the mooring period and those that might have been present in the seawater used for sample splitting.
Blank B	50 mL of Milli Q water.

Table S3. Recovery rate

Run	Recovered/used particles.
1	92/100
2	94/100
3	96/100
4	91/100
5	100/100
Mean recovery rate (%)	94.6

Table S4. Sea surface temperature (SST) and mixed layer depth (MLD) around stn. KEO (144°E–145°E, 32°N–33°N) obtained from the Coperncius Marine Service

Date	MLD (m)	SST (°C)
2014/7/2	13	23.6
2014/7/9	13	23.2
2014/7/16	12	24.2
2014/7/23	11	25.0
2014/7/30	11	26.0
2014/8/6	11	28.2
2014/8/13	11	27.5
2014/8/20	12	28.5
2014/8/27	12	28.3
2014/9/3	12	27.9
2014/9/10	16	25.4
2014/9/17	31	25.7
2014/9/24	31	25.3
2014/10/1	31	24.4
2014/10/8	35	25.2
2014/10/15	44	24.0
2014/10/22	51	24.1
2014/10/29	44	23.8
2014/11/5	39	23.7
2014/11/12	46	22.9
2014/11/19	50	22.3
2014/11/26	52	22.1
2014/12/3	62	21.5
2014/12/10	70	21.0
2014/12/17	108	20.1
2014/12/24	133	19.8
2015/1/1	151	19.4
2015/1/8	179	19.0
2015/1/15	223	18.6
2015/1/22	218	18.3
2015/1/29	265	17.3
2015/2/5	231	17.3

2015/2/12	174	17.3
2015/2/19	166	17.4
2015/2/26	207	17.3
2015/3/5	160	16.7
2015/3/12	134	16.7
2015/3/19	56	17.3
2015/3/26	61	17.2
2015/4/2	20	17.6
2015/4/9	20	18.9
2015/4/16	16	19.7
2015/4/23	14	19.8
2015/4/30	15	21.2
2015/5/7	26	21.5
2015/5/14	29	21.4
2015/5/21	17	21.4
2015/5/28	16	21.4
2015/6/4	20	21.8
2015/6/11	18	22.4
2015/6/18	19	23.0
2015/6/25	24	23.4
2015/7/2	32	23.4
2015/7/9	30	26.7
2015/7/16	11	26.3
2015/7/23	12	27.9
2015/7/30	12	27.5
2015/8/6	12	28.6
2015/8/13	12	26.5
2015/8/20	14	27.4
2015/8/27	18	25.9
2015/9/3	33	27.0
2015/9/10	32	27.6
2015/9/17	29	27.6
2015/9/24	30	27.3
2015/10/1	32	26.8
2015/10/8	41	25.2
2015/10/15	52	24.9

2015/10/22	53	24.5
2015/10/29	45	24.1
2015/11/5	38	23.6
2015/11/12	49	23.6
2015/11/19	60	23.9
2015/11/26	74	23.2
2015/12/3	37	22.2
2015/12/10	57	21.7
2015/12/17	73	22.1
2015/12/24	52	21.0
2016/1/1	116	20.4
2016/1/8	135	20.6
2016/1/15	134	19.7
2016/1/22	155	18.9
2016/1/29	156	18.5
2016/2/5	138	18.6
2016/2/12	140	18.9
2016/2/19	163	18.7
2016/2/26	138	18.5
2016/3/4	141	18.3
2016/3/11	64	18.6
2016/3/18	172	18.5
2016/3/25	204	18.9
2016/4/1	143	18.2
2016/4/8	274	18.3
2016/4/15	31	19.0
2016/4/22	114	19.3
2016/4/29	25	19.9
2016/5/6	23	20.2
2016/5/13	24	19.6
2016/5/20	15	20.3
2016/5/27	23	21.6
2016/6/3	18	22.5
2016/6/10	23	22.5
2016/6/17	25	23.2
2016/6/24	21	23.5

2016/7/1	16	23.9
2016/7/8	13	25.6
2016/7/15	12	25.6
2016/7/22	11	26.5
2016/7/29	12	26.7
2016/8/5	13	26.9
2016/8/12	16	26.3
2016/8/19	22	26.0
2016/8/26	30	27.1
2016/9/2	24	27.6
2016/9/9	13	27.4
2016/9/16	15	27.9
2016/9/23	23	28.2
2016/9/30	24	27.9

Table S5. Sea surface height anomaly around Stn. KEO

Date	SSHA (m)
2014/7/1	0.05
2014/7/2	0.02
2014/7/3	-0.01
2014/7/4	-0.04
2014/7/5	-0.07
2014/7/6	-0.11
2014/7/7	-0.14
2014/7/8	-0.17
2014/7/9	-0.20
2014/7/10	-0.22
2014/7/11	-0.25
2014/7/12	-0.27
2014/7/13	-0.30
2014/7/14	-0.32
2014/7/15	-0.35
2014/7/16	-0.36
2014/7/17	-0.38
2014/7/18	-0.40
2014/7/19	-0.42
2014/7/20	-0.43
2014/7/21	-0.44
2014/7/22	-0.45
2014/7/23	-0.46
2014/7/24	-0.46
2014/7/25	-0.45
2014/7/26	-0.44
2014/7/27	-0.42
2014/7/28	-0.39
2014/7/29	-0.35
2014/7/30	-0.32
2014/7/31	-0.29
2014/8/1	-0.25
2014/8/2	-0.21
2014/8/3	-0.17
2014/8/4	-0.13
2014/8/5	-0.08
2014/8/6	-0.05
2014/8/7	-0.01
2014/8/8	0.03
2014/8/9	0.06
2014/8/10	0.09
2014/8/11	0.11
2014/8/12	0.13

2014/8/13	0.15
2014/8/14	0.16
2014/8/15	0.17
2014/8/16	0.17
2014/8/17	0.18
2014/8/18	0.18
2014/8/19	0.18
2014/8/20	0.18
2014/8/21	0.18
2014/8/22	0.18
2014/8/23	0.18
2014/8/24	0.18
2014/8/25	0.18
2014/8/26	0.18
2014/8/27	0.17
2014/8/28	0.17
2014/8/29	0.17
2014/8/30	0.17
2014/8/31	0.17
2014/9/1	0.17
2014/9/2	0.18
2014/9/3	0.18
2014/9/4	0.19
2014/9/5	0.20
2014/9/6	0.21
2014/9/7	0.22
2014/9/8	0.22
2014/9/9	0.23
2014/9/10	0.23
2014/9/11	0.23
2014/9/12	0.24
2014/9/13	0.24
2014/9/14	0.24
2014/9/15	0.24
2014/9/16	0.23
2014/9/17	0.23
2014/9/18	0.22
2014/9/19	0.21
2014/9/20	0.20
2014/9/21	0.19
2014/9/22	0.18
2014/9/23	0.17
2014/9/24	0.16
2014/9/25	0.16
2014/9/26	0.15
2014/9/27	0.15

2014/9/28	0.14
2014/9/29	0.13
2014/9/30	0.12
2014/10/1	0.11
2014/10/2	0.11
2014/10/3	0.10
2014/10/4	0.09
2014/10/5	0.08
2014/10/6	0.06
2014/10/7	0.05
2014/10/8	0.03
2014/10/9	0.02
2014/10/10	0.01
2014/10/11	0.01
2014/10/12	0.00
2014/10/13	-0.01
2014/10/14	-0.02
2014/10/15	-0.03
2014/10/16	-0.02
2014/10/17	-0.02
2014/10/18	-0.02
2014/10/19	-0.01
2014/10/20	-0.01
2014/10/21	-0.01
2014/10/22	-0.01
2014/10/23	0.00
2014/10/24	0.01
2014/10/25	0.00
2014/10/26	0.00
2014/10/27	-0.01
2014/10/28	-0.02
2014/10/29	-0.04
2014/10/30	-0.06
2014/10/31	-0.08
2014/11/1	-0.10
2014/11/2	-0.12
2014/11/3	-0.15
2014/11/4	-0.17
2014/11/5	-0.19
2014/11/6	-0.22
2014/11/7	-0.24
2014/11/8	-0.26
2014/11/9	-0.28
2014/11/10	-0.29
2014/11/11	-0.31
2014/11/12	-0.32

2014/11/13	-0.33
2014/11/14	-0.34
2014/11/15	-0.34
2014/11/16	-0.35
2014/11/17	-0.35
2014/11/18	-0.34
2014/11/19	-0.34
2014/11/20	-0.33
2014/11/21	-0.33
2014/11/22	-0.32
2014/11/23	-0.30
2014/11/24	-0.29
2014/11/25	-0.27
2014/11/26	-0.26
2014/11/27	-0.24
2014/11/28	-0.22
2014/11/29	-0.20
2014/11/30	-0.18
2014/12/1	-0.16
2014/12/2	-0.14
2014/12/3	-0.12
2014/12/4	-0.10
2014/12/5	-0.09
2014/12/6	-0.07
2014/12/7	-0.05
2014/12/8	-0.04
2014/12/9	-0.02
2014/12/10	-0.01
2014/12/11	0.00
2014/12/12	0.01
2014/12/13	0.01
2014/12/14	0.02
2014/12/15	0.03
2014/12/16	0.03
2014/12/17	0.03
2014/12/18	0.04
2014/12/19	0.04
2014/12/20	0.04
2014/12/21	0.04
2014/12/22	0.05
2014/12/23	0.05
2014/12/24	0.05
2014/12/25	0.05
2014/12/26	0.06
2014/12/27	0.06
2014/12/28	0.06

2014/12/29	0.07
2014/12/30	0.07
2014/12/31	0.08
2015/1/1	0.08
2015/1/2	0.09
2015/1/3	0.09
2015/1/4	0.09
2015/1/5	0.09
2015/1/6	0.10
2015/1/7	0.10
2015/1/8	0.10
2015/1/9	0.10
2015/1/10	0.10
2015/1/11	0.10
2015/1/12	0.10
2015/1/13	0.10
2015/1/14	0.10
2015/1/15	0.09
2015/1/16	0.09
2015/1/17	0.09
2015/1/18	0.08
2015/1/19	0.08
2015/1/20	0.07
2015/1/21	0.06
2015/1/22	0.06
2015/1/23	0.06
2015/1/24	0.06
2015/1/25	0.05
2015/1/26	0.05
2015/1/27	0.05
2015/1/28	0.04
2015/1/29	0.03
2015/1/30	0.02
2015/1/31	0.01
2015/2/1	0.00
2015/2/2	-0.01
2015/2/3	-0.03
2015/2/4	-0.04
2015/2/5	-0.06
2015/2/6	-0.07
2015/2/7	-0.09
2015/2/8	-0.11
2015/2/9	-0.13
2015/2/10	-0.15
2015/2/11	-0.16
2015/2/12	-0.18

2015/2/13	-0.20
2015/2/14	-0.21
2015/2/15	-0.22
2015/2/16	-0.23
2015/2/17	-0.23
2015/2/18	-0.23
2015/2/19	-0.24
2015/2/20	-0.23
2015/2/21	-0.23
2015/2/22	-0.23
2015/2/23	-0.23
2015/2/24	-0.22
2015/2/25	-0.22
2015/2/26	-0.22
2015/2/27	-0.21
2015/2/28	-0.21
2015/3/1	-0.21
2015/3/2	-0.21
2015/3/3	-0.20
2015/3/4	-0.20
2015/3/5	-0.20
2015/3/6	-0.20
2015/3/7	-0.20
2015/3/8	-0.20
2015/3/9	-0.21
2015/3/10	-0.21
2015/3/11	-0.22
2015/3/12	-0.23
2015/3/13	-0.24
2015/3/14	-0.25
2015/3/15	-0.26
2015/3/16	-0.26
2015/3/17	-0.27
2015/3/18	-0.28
2015/3/19	-0.29
2015/3/20	-0.30
2015/3/21	-0.31
2015/3/22	-0.32
2015/3/23	-0.32
2015/3/24	-0.32
2015/3/25	-0.32
2015/3/26	-0.33
2015/3/27	-0.33
2015/3/28	-0.34
2015/3/29	-0.34
2015/3/30	-0.34

2015/3/31	-0.34
2015/4/1	-0.34
2015/4/2	-0.33
2015/4/3	-0.32
2015/4/4	-0.32
2015/4/5	-0.32
2015/4/6	-0.31
2015/4/7	-0.30
2015/4/8	-0.31
2015/4/9	-0.31
2015/4/10	-0.34
2015/4/11	-0.36
2015/4/12	-0.38
2015/4/13	-0.40
2015/4/14	-0.42
2015/4/15	-0.44
2015/4/16	-0.45
2015/4/17	-0.47
2015/4/18	-0.48
2015/4/19	-0.48
2015/4/20	-0.48
2015/4/21	-0.49
2015/4/22	-0.49
2015/4/23	-0.48
2015/4/24	-0.46
2015/4/25	-0.45
2015/4/26	-0.42
2015/4/27	-0.40
2015/4/28	-0.37
2015/4/29	-0.34
2015/4/30	-0.30
2015/5/1	-0.28
2015/5/2	-0.26
2015/5/3	-0.24
2015/5/4	-0.22
2015/5/5	-0.20
2015/5/6	-0.18
2015/5/7	-0.17
2015/5/8	-0.15
2015/5/9	-0.14
2015/5/10	-0.12
2015/5/11	-0.11
2015/5/12	-0.09
2015/5/13	-0.07
2015/5/14	-0.06
2015/5/15	-0.05

2015/5/16	-0.04
2015/5/17	-0.03
2015/5/18	-0.02
2015/5/19	-0.01
2015/5/20	-0.01
2015/5/21	0.00
2015/5/22	0.00
2015/5/23	0.00
2015/5/24	0.01
2015/5/25	0.01
2015/5/26	0.01
2015/5/27	0.01
2015/5/28	0.02
2015/5/29	0.01
2015/5/30	0.01
2015/5/31	0.02
2015/6/1	0.01
2015/6/2	0.00
2015/6/3	0.00
2015/6/4	0.00
2015/6/5	0.00
2015/6/6	0.00
2015/6/7	0.00
2015/6/8	0.00
2015/6/9	0.00
2015/6/10	-0.01
2015/6/11	-0.01
2015/6/12	0.00
2015/6/13	0.01
2015/6/14	0.01
2015/6/15	0.01
2015/6/16	0.02
2015/6/17	0.02
2015/6/18	0.03
2015/6/19	0.04
2015/6/20	0.04
2015/6/21	0.05
2015/6/22	0.06
2015/6/23	0.07
2015/6/24	0.08
2015/6/25	0.09
2015/6/26	0.10
2015/6/27	0.11
2015/6/28	0.12
2015/6/29	0.13
2015/6/30	0.14

2015/7/1	0.14
2015/7/2	0.15
2015/7/3	0.16
2015/7/4	0.16
2015/7/5	0.17
2015/7/6	0.17
2015/7/7	0.17
2015/7/8	0.16
2015/7/9	0.16
2015/7/10	0.16
2015/7/11	0.15
2015/7/12	0.14
2015/7/13	0.13
2015/7/14	0.12
2015/7/15	0.11
2015/7/16	0.09
2015/7/17	0.08
2015/7/18	0.07
2015/7/19	0.06
2015/7/20	0.06
2015/7/21	0.05
2015/7/22	0.05
2015/7/23	0.05
2015/7/24	0.06
2015/7/25	0.06
2015/7/26	0.07
2015/7/27	0.07
2015/7/28	0.08
2015/7/29	0.09
2015/7/30	0.09
2015/7/31	0.10
2015/8/1	0.10
2015/8/2	0.11
2015/8/3	0.11
2015/8/4	0.12
2015/8/5	0.12
2015/8/6	0.12
2015/8/7	0.12
2015/8/8	0.13
2015/8/9	0.13
2015/8/10	0.13
2015/8/11	0.13
2015/8/12	0.13
2015/8/13	0.13
2015/8/14	0.13
2015/8/15	0.12

2015/8/16	0.12
2015/8/17	0.12
2015/8/18	0.13
2015/8/19	0.13
2015/8/20	0.13
2015/8/21	0.13
2015/8/22	0.13
2015/8/23	0.14
2015/8/24	0.15
2015/8/25	0.15
2015/8/26	0.16
2015/8/27	0.17
2015/8/28	0.19
2015/8/29	0.20
2015/8/30	0.21
2015/8/31	0.22
2015/9/1	0.23
2015/9/2	0.24
2015/9/3	0.25
2015/9/4	0.25
2015/9/5	0.26
2015/9/6	0.26
2015/9/7	0.27
2015/9/8	0.27
2015/9/9	0.28
2015/9/10	0.28
2015/9/11	0.28
2015/9/12	0.29
2015/9/13	0.29
2015/9/14	0.30
2015/9/15	0.30
2015/9/16	0.31
2015/9/17	0.31
2015/9/18	0.32
2015/9/19	0.33
2015/9/20	0.34
2015/9/21	0.35
2015/9/22	0.36
2015/9/23	0.37
2015/9/24	0.37
2015/9/25	0.38
2015/9/26	0.38
2015/9/27	0.38
2015/9/28	0.38
2015/9/29	0.37
2015/9/30	0.37

2015/10/1	0.36
2015/10/2	0.35
2015/10/3	0.34
2015/10/4	0.33
2015/10/5	0.31
2015/10/6	0.30
2015/10/7	0.29
2015/10/8	0.29
2015/10/9	0.28
2015/10/10	0.27
2015/10/11	0.26
2015/10/12	0.25
2015/10/13	0.24
2015/10/14	0.24
2015/10/15	0.23
2015/10/16	0.23
2015/10/17	0.23
2015/10/18	0.23
2015/10/19	0.22
2015/10/20	0.22
2015/10/21	0.22
2015/10/22	0.22
2015/10/23	0.22
2015/10/24	0.22
2015/10/25	0.22
2015/10/26	0.22
2015/10/27	0.23
2015/10/28	0.24
2015/10/29	0.24
2015/10/30	0.25
2015/10/31	0.26
2015/11/1	0.26
2015/11/2	0.27
2015/11/3	0.28
2015/11/4	0.28
2015/11/5	0.29
2015/11/6	0.29
2015/11/7	0.29
2015/11/8	0.29
2015/11/9	0.29
2015/11/10	0.29
2015/11/11	0.29
2015/11/12	0.29
2015/11/13	0.30
2015/11/14	0.30
2015/11/15	0.31

2015/11/16	0.31
2015/11/17	0.31
2015/11/18	0.32
2015/11/19	0.32
2015/11/20	0.33
2015/11/21	0.33
2015/11/22	0.34
2015/11/23	0.34
2015/11/24	0.35
2015/11/25	0.36
2015/11/26	0.36
2015/11/27	0.37
2015/11/28	0.37
2015/11/29	0.38
2015/11/30	0.38
2015/12/1	0.39
2015/12/2	0.39
2015/12/3	0.39
2015/12/4	0.39
2015/12/5	0.39
2015/12/6	0.39
2015/12/7	0.39
2015/12/8	0.40
2015/12/9	0.40
2015/12/10	0.40
2015/12/11	0.40
2015/12/12	0.40
2015/12/13	0.40
2015/12/14	0.40
2015/12/15	0.40
2015/12/16	0.40
2015/12/17	0.40
2015/12/18	0.40
2015/12/19	0.40
2015/12/20	0.40
2015/12/21	0.39
2015/12/22	0.38
2015/12/23	0.37
2015/12/24	0.36
2015/12/25	0.35
2015/12/26	0.33
2015/12/27	0.31
2015/12/28	0.29
2015/12/29	0.27
2015/12/30	0.25
2015/12/31	0.23

2016/1/1	0.22
2016/1/2	0.20
2016/1/3	0.18
2016/1/4	0.17
2016/1/5	0.17
2016/1/6	0.16
2016/1/7	0.15
2016/1/8	0.15
2016/1/9	0.15
2016/1/10	0.15
2016/1/11	0.15
2016/1/12	0.16
2016/1/13	0.17
2016/1/14	0.16
2016/1/15	0.16
2016/1/16	0.16
2016/1/17	0.16
2016/1/18	0.15
2016/1/19	0.15
2016/1/20	0.14
2016/1/21	0.13
2016/1/22	0.11
2016/1/23	0.09
2016/1/24	0.08
2016/1/25	0.06
2016/1/26	0.04
2016/1/27	0.02
2016/1/28	0.00
2016/1/29	-0.01
2016/1/30	-0.03
2016/1/31	-0.04
2016/2/1	-0.06
2016/2/2	-0.07
2016/2/3	-0.07
2016/2/4	-0.07
2016/2/5	-0.07
2016/2/6	-0.08
2016/2/7	-0.08
2016/2/8	-0.08
2016/2/9	-0.09
2016/2/10	-0.09
2016/2/11	-0.09
2016/2/12	-0.08
2016/2/13	-0.09
2016/2/14	-0.09
2016/2/15	-0.09

2016/2/16	-0.09
2016/2/17	-0.10
2016/2/18	-0.10
2016/2/19	-0.11
2016/2/20	-0.11
2016/2/21	-0.11
2016/2/22	-0.11
2016/2/23	-0.11
2016/2/24	-0.11
2016/2/25	-0.11
2016/2/26	-0.10
2016/2/27	-0.10
2016/2/28	-0.09
2016/2/29	-0.07
2016/3/1	-0.06
2016/3/2	-0.04
2016/3/3	-0.01
2016/3/4	0.00
2016/3/5	0.02
2016/3/6	0.04
2016/3/7	0.06
2016/3/8	0.08
2016/3/9	0.10
2016/3/10	0.12
2016/3/11	0.14
2016/3/12	0.15
2016/3/13	0.17
2016/3/14	0.18
2016/3/15	0.19
2016/3/16	0.20
2016/3/17	0.21
2016/3/18	0.21
2016/3/19	0.21
2016/3/20	0.22
2016/3/21	0.22
2016/3/22	0.22
2016/3/23	0.22
2016/3/24	0.22
2016/3/25	0.22
2016/3/26	0.22
2016/3/27	0.22
2016/3/28	0.22
2016/3/29	0.22
2016/3/30	0.23
2016/3/31	0.23
2016/4/1	0.23

2016/4/2	0.23
2016/4/3	0.24
2016/4/4	0.24
2016/4/5	0.25
2016/4/6	0.25
2016/4/7	0.25
2016/4/8	0.26
2016/4/9	0.26
2016/4/10	0.27
2016/4/11	0.27
2016/4/12	0.27
2016/4/13	0.27
2016/4/14	0.27
2016/4/15	0.27
2016/4/16	0.26
2016/4/17	0.26
2016/4/18	0.25
2016/4/19	0.25
2016/4/20	0.24
2016/4/21	0.23
2016/4/22	0.22
2016/4/23	0.21
2016/4/24	0.20
2016/4/25	0.19
2016/4/26	0.17
2016/4/27	0.16
2016/4/28	0.15
2016/4/29	0.14
2016/4/30	0.13
2016/5/1	0.12
2016/5/2	0.11
2016/5/3	0.11
2016/5/4	0.10
2016/5/5	0.09
2016/5/6	0.09
2016/5/7	0.08
2016/5/8	0.08
2016/5/9	0.08
2016/5/10	0.08
2016/5/11	0.09
2016/5/12	0.09
2016/5/13	0.08
2016/5/14	0.08
2016/5/15	0.08
2016/5/16	0.07
2016/5/17	0.07

2016/5/18	0.07
2016/5/19	0.07
2016/5/20	0.07
2016/5/21	0.08
2016/5/22	0.08
2016/5/23	0.09
2016/5/24	0.09
2016/5/25	0.10
2016/5/26	0.11
2016/5/27	0.11
2016/5/28	0.12
2016/5/29	0.13
2016/5/30	0.14
2016/5/31	0.15
2016/6/1	0.16
2016/6/2	0.17
2016/6/3	0.18
2016/6/4	0.19
2016/6/5	0.19
2016/6/6	0.20
2016/6/7	0.21
2016/6/8	0.22
2016/6/9	0.22
2016/6/10	0.23
2016/6/11	0.24
2016/6/12	0.24
2016/6/13	0.24
2016/6/14	0.24
2016/6/15	0.25
2016/6/16	0.25
2016/6/17	0.25
2016/6/18	0.26
2016/6/19	0.26
2016/6/20	0.26
2016/6/21	0.26
2016/6/22	0.26
2016/6/23	0.26
2016/6/24	0.26
2016/6/25	0.26
2016/6/26	0.26
2016/6/27	0.26
2016/6/28	0.26
2016/6/29	0.26
2016/6/30	0.26
2016/7/1	0.26
2016/7/2	0.25

2016/7/3	0.25
2016/7/4	0.25
2016/7/5	0.25
2016/7/6	0.24
2016/7/7	0.24
2016/7/8	0.23
2016/7/9	0.23
2016/7/10	0.22
2016/7/11	0.22
2016/7/12	0.21
2016/7/13	0.20
2016/7/14	0.19
2016/7/15	0.18
2016/7/16	0.17
2016/7/17	0.16
2016/7/18	0.15
2016/7/19	0.15
2016/7/20	0.14
2016/7/21	0.14
2016/7/22	0.14
2016/7/23	0.14
2016/7/24	0.14
2016/7/25	0.15
2016/7/26	0.15
2016/7/27	0.15
2016/7/28	0.15
2016/7/29	0.15
2016/7/30	0.15
2016/7/31	0.15
2016/8/1	0.16
2016/8/2	0.15
2016/8/3	0.15
2016/8/4	0.15
2016/8/5	0.14
2016/8/6	0.13
2016/8/7	0.13
2016/8/8	0.12
2016/8/9	0.12
2016/8/10	0.12
2016/8/11	0.12
2016/8/12	0.12
2016/8/13	0.12
2016/8/14	0.12
2016/8/15	0.12
2016/8/16	0.12
2016/8/17	0.12

2016/8/18	0.12
2016/8/19	0.12
2016/8/20	0.12
2016/8/21	0.12
2016/8/22	0.11
2016/8/23	0.11
2016/8/24	0.11
2016/8/25	0.10
2016/8/26	0.10
2016/8/27	0.10
2016/8/28	0.09
2016/8/29	0.09
2016/8/30	0.09
2016/8/31	0.09
2016/9/1	0.09
2016/9/2	0.10
2016/9/3	0.10
2016/9/4	0.10
2016/9/5	0.11
2016/9/6	0.12
2016/9/7	0.13
2016/9/8	0.14
2016/9/9	0.16
2016/9/10	0.18
2016/9/11	0.19
2016/9/12	0.20
2016/9/13	0.22
2016/9/14	0.23
2016/9/15	0.24
2016/9/16	0.24
2016/9/17	0.24
2016/9/18	0.24
2016/9/19	0.25
2016/9/20	0.24
2016/9/21	0.24
2016/9/22	0.23
2016/9/23	0.21
2016/9/24	0.20
2016/9/25	0.18
2016/9/26	0.16
2016/9/27	0.14
2016/9/28	0.10
2016/9/29	0.07
2016/9/30	0.04
2016/10/1	0.01
2016/10/2	-0.02

Table S6. Primary productivity calculated from satellite data acquired from MODIS Aqua and the Vertically Generalized Production Model

Date	Surface chlorophyll-*a* (mg m^{-3})	SST (°C)	Photosynthetically active radiation (Einstein m^{-2} day^{-1})	Depth of euphotic zone (m)	Daylength (h)	Primary productivity (mg-C m^{-2} day^{-1})
2014/7/4		22.9665	31.3088		14.0290	
2014/7/12	0.1092	23.9982	49.7863	82.7509	13.9398	447
2014/7/20	0.1095	25.6277	47.2261	85.6996	13.8140	411
2014/7/28	0.0900	26.6654	57.1218	99.4364	13.6542	362
2014/8/5	0.0666	27.7590	53.7774	106.7248	13.4669	256
2014/8/13	0.0637	28.0648	51.2213	118.5069	13.2546	258
2014/8/21	0.0561	28.8647	47.0369	117.4228	13.0246	201
2014/8/29	0.0644	28.0016	48.9501	115.5686	12.7787	246
2014/9/6	0.0725	26.6466	33.9011	109.2414	12.5226	281
2014/9/14	0.1035	25.5145	31.0310	84.1284	12.2579	327
2014/9/22	0.0840	24.7126	35.3057	102.0506	11.9897	338
2014/9/30	0.1264	24.6112	32.3690	87.6992	11.7192	426
2014/10/8	0.1336	24.3917	29.9988	82.1391	11.4513	415
2014/10/1	0.1213	23.7114	31.0925	80.2169	11.1876	375
2014/10/2	0.1199	23.5579	28.8488	89.7095	10.9338	405
2014/11/1	0.1289	22.9729	16.5409	81.6067	10.6929	365
2014/11/9	0.1297	22.8248	23.2388	88.4779	10.4719	417
2014/11/1	0.1458	22.0084	21.0901	73.3908	10.2751	388
2014/11/2	0.1282	21.6710	18.0352	76.6002	10.1101	345
2014/12/3	0.1487	20.9984	15.9741	66.0245	9.9822	338
2014/12/1	0.1516	20.3309	16.8632	55.6634	9.8974	294
2014/12/1	0.1346	19.2302	11.9906	58.3901	9.8592	253
2014/12/2	0.1779	18.9862	16.1203	60.6805	9.8699	371
2015/1/1	0.1135	18.6117	15.1693	78.3164	9.9011	301
2015/1/9	0.1414	18.4067	17.6327	63.5687	9.9889	315
2015/1/17	0.1358	18.0230	16.5358	63.0250	10.1199	298
2015/1/25	0.1557	17.3831	18.4982	73.8857	10.2885	408
2015/2/2	0.1493	17.2870	22.1911	68.2302	10.4888	379
2015/2/10	0.1468	17.0317	24.3422	72.6979	10.7140	407
2015/2/18	0.1648	16.4342	28.5795	65.9703	10.9589	423
2015/2/26	0.2205	16.7536	31.9528	62.6337	11.2170	565
2015/3/6		16.3687	27.9827		11.4847	
2015/3/14	0.2360	16.8531	24.4482	56.0979	11.7570	551
2015/3/22	0.3377	16.6460	39.2209	45.2988	12.0311	683
2015/3/30	0.4984	16.7658	45.5848	39.9979	12.3029	926
2015/4/7		16.3847	28.9062		12.5701	
2015/4/15		18.8669	46.9835		12.8281	
2015/4/23	0.1912	19.9619	42.4806	59.9733	13.0751	599
2015/5/1	0.1586	20.4948	39.2411	64.4270	13.3040	537
2015/5/9	0.1365	20.5813	54.9118	78.1519	13.5136	584

2015/5/17		19.9441	31.3495		13.6962	
2015/5/25	0.1405	21.4349	42.6082	88.5961	13.8495	674
2015/6/2		21.4110	35.9370		13.9668	
2015/6/10		22.5165	34.5681		14.0458	
2015/6/18	0.0881	22.9435	28.5603	90.9406	14.0828	400
2015/6/26	0.0870	23.4467	41.8269	87.9447	14.0771	388
2015/7/4	0.0828	25.3063	58.7085	91.5979	14.0291	351
2015/7/12	0.0820	25.9442	59.3919	97.5112	13.9400	351
2015/7/20	0.0661	27.5834	57.0432	109.1374	13.8143	272
2015/7/28	0.0583	28.2140	56.1047	128.8124	13.6545	262
2015/8/5	0.0559	28.9651	45.8391	119.3129	13.4673	207
2015/8/13	0.0583	27.4468	55.4253	126.7583	13.2550	270
2015/8/21	0.0561	27.2449	39.8782	179.7504	13.0252	359
2015/8/29	0.0713	26.6831	51.3688	106.6493	12.7788	285
2015/9/6	0.0591	27.0873	50.0467	129.9128	12.5231	272
2015/9/14	0.0608	27.2092	38.7963	110.3864	12.2587	225
2015/9/22	0.0695	27.1005	29.8253	118.7458	11.9903	266
2015/9/30	0.0846	26.2592	36.7609	103.9527	11.7200	304
2015/10/8	0.1057	24.4616	33.5619	100.5500	11.4518	405
2015/10/1	0.1126	23.8219	19.8895	94.0940	11.1883	381
2015/10/2	0.1147	23.9537	28.7194	87.0824	10.9344	368
2015/11/1	0.1334	23.4004	22.6584	79.9305	10.6936	383
2015/11/9	0.1617	23.0777	20.3892	70.0889	10.4723	398
2015/11/1	0.1447	22.7867	16.4017	76.0288	10.2756	370
2015/11/2	0.1841	21.8699	20.9131	59.5737	10.1105	392
2015/12/3	0.1825	21.6755	16.7390	58.0563	9.9825	362
2015/12/1	0.2088	21.3085	12.6437	55.1558	9.8975	370
2015/12/1	0.1817	20.8394	19.4156	56.7971	9.8593	365
2015/12/2	0.1986	20.2737	18.3470	62.6322	9.8698	439
2016/1/1	0.1719	19.6349	13.6239	65.0149	9.9011	373
2016/1/9	0.1471	19.6489	15.5225	68.3365	9.9892	348
2016/1/17	0.1471	18.9162	13.9115	65.1828	10.1200	327
2016/1/25	0.1537	18.6625	20.2061	62.4747	10.2889	357
2016/2/2	0.1880	18.7361	19.5922	55.1145	10.4889	391
2016/2/10	0.2432	18.4231	21.7101	53.9221	10.7147	512
2016/2/18	0.3670	17.6239	20.0653	48.4852	10.9590	687
2016/2/26	0.1918	18.1184	27.8325	59.8080	11.2177	482
2016/3/5	0.2064	18.2141	30.4664	59.6894	11.4849	538
2016/3/13	0.1847	18.6164	35.6825	60.1872	11.7577	509
2016/3/21	0.2515	18.7146	32.3236	54.9371	12.0312	642
2016/3/29	0.2518	18.3794	41.7777	57.5544	12.3036	702
2016/4/6		18.3486	32.1435		12.5701	
2016/4/14	0.2584	18.8814	37.8122	52.6019	12.8293	686
2016/4/22		19.1281	28.3299		13.0746	
2016/4/30	0.1557	19.3125	47.8662	77.4861	13.3051	647
2016/5/8	0.1000	19.2212	43.4228	71.1292	13.5132	384

2016/5/16	0.0927	20.6586	48.6331	76.4482	13.6970	390
2016/5/24	0.1123	20.4906	45.8259	94.7112	13.8492	590
2016/6/1	0.1113	22.1173	35.7396	88.7401	13.9672	519
2016/6/9	0.0813	18.8917	45.6618	85.6187	14.0457	391
2016/6/17	0.0969	22.6028	49.6736	90.8369	14.0829	472
2016/6/25	0.0918	23.8921	56.6444	89.9327	14.0772	419
2016/7/3	0.0808	25.1487	49.8230	104.6276	14.0288	391
2016/7/11	0.0669	25.2946	50.3034	102.2830	13.9403	311
2016/7/19	0.0595	25.7475	52.8249	120.8177	13.8137	315
2016/7/27	0.0687	26.6472	51.7082	104.7827	13.6548	289
2016/8/4	0.0671	26.5795	47.4864	113.7825	13.4663	302
2016/8/12	0.0712	26.0024	39.0748	106.8711	13.2555	305
2016/8/20	0.0662	26.4999	48.4140	121.1632	13.0240	310
2016/8/28	0.1159	27.0130	48.7043	82.6516	12.7793	348
2016/9/5	0.0860	27.6137	47.8575	92.6599	12.5225	268
2016/9/13	0.0889	27.9903	43.9599	93.4037	12.2586	262
2016/9/21	0.0850	27.7764	40.1395	100.1941	11.9896	266
2016/9/29	0.0877	27.3481	36.4608	90.7321	11.7199	250

Table S7. Fluxes of sinking particles and microplastics (MPs) at Stns. KEO and Kiel 276

	Stn. KEO	Stn. Kiel 276
Latitude, longitude	32°22′N, 144°25'E	33°N, 22°W
Water depth (m)<br>(Mooring depth)	5900<br>(4900)	5300<br>(2000)
Sampling interval	1 July 2014<br>-2 October 2016	20 April 2003<br>-1 July 2015
Total mass flux (mg m^{-2} day^{-1})<br>(Mean ± S.D.)	11.5–144.8<br>(52.0 ± 26.8)	0.2–246.9<br>(50.4 ± 50.3)
Organic carbon flux (mg m^{-2} day^{-1})<br>(Mean ± S.D.)	0.51–6.7<br>(2.3 ± 1.2)	-
MP particle size (μm)<br>(Mean ± S.D.)	20–480<br>(66 ± 53)	(88 ± 113)
MP aspect ratio<br>Mean ± S.D.	0.75 ± 0.20	-
MP Shannon diversity (H′)	3.0	-
MP abundance flux (pieces m^{-2} day^{-1})<br>(Mean ± S.D)	111–889<br>(352 ± 194)	1–3147<br>(421 ± 715)
MP mass flux (mg $m^{-2}day^{-1}$)<br>(Mean ± S.D)	4.5×10^{-3}–0.38<br>(0.054 ± 0.075)	9.6×10^{-5}–4.8<br>(0.18 ± 0.55)
MP carbon flux (mg m^{-2} day^{-1})<br>(Mean ± S.D)	3.2×10^{-3}–0.24<br>(0.035 ± 0.048)	8.8×10^{-5}–4.1<br>(0.14 ± 0.46)
Reference	This study	Reineccius and Waniek[6]

Table S8. Summary of individual microplastic particles observed in sediment trap samples at Stn. KEO. Polymer-type abbreviations are as shown in Table S1

Sample number	Microplastic serial number	Major axis (μm)	Minor axis (μm)	Polymer type	Shape type	Aspect ratio	Volume (cm^3)	Mass (g)
KEOST2014-2015 #1	1	30	30	ABS	fragment	1.00	1.1E-08	1.2E-08
KEOST2014-2015 #1	2	40	30	PA	fragment	0.75	1.4E-08	1.6E-08
KEOST2014-2015 #1	3	40	30	PA	fragment	0.75	1.4E-08	1.6E-08
KEOST2014-2015 #1	4	70	50	PBT	fragment	0.71	6.9E-08	9.3E-08
KEOST2014-2015 #1	5	100	90	PE	fragment	0.90	3.2E-07	3.0E-07
KEOST2014-2015 #1	6	60	40	PE	fragment	0.67	3.8E-08	3.6E-08
KEOST2014-2015 #1	7	30	20	PE	fragment	0.67	4.7E-09	4.4E-09
KEOST2014-2015 #1	8	70	30	PE	fragment	0.43	2.5E-08	2.3E-08
KEOST2014-2015 #1	9	40	30	PE	fragment	0.75	1.4E-08	1.3E-08
KEOST2014-2015 #1	10	90	50	PET	fragment	0.56	8.8E-08	1.2E-07
KEOST2014-2015 #1	11	120	30	PET	fiber	-	3.4E-08	4.6E-08
KEOST2014-2015 #1	12	150	30	PET	fragment	0.20	5.3E-08	7.2E-08
KEOST2014-2015 #1	13	80	30	PET	fiber	-	2.3E-08	3.1E-08
KEOST2014-2015 #1	14	870	50	PET	fiber	-	6.8E-07	9.2E-07
KEOST2014-2015 #1	15	320	40	PET	fiber	-	1.6E-07	2.2E-07
KEOST2014-2015 #1	16	100	60	PET	fragment	0.60	1.4E-07	1.9E-07
KEOST2014-2015 #1	17	30	30	PP	fragment	1.00	1.1E-08	9.6E-09
KEOST2014-2015 #1	18	50	30	PP	fragment	0.60	1.8E-08	1.6E-08
KEOST2014-2015 #1	19	400	60	PU	fiber	-	4.5E-07	5.3E-07
KEOST2014-2015 #2	20	80	50	EP	fragment	0.63	7.9E-08	1.0E-07
KEOST2014-2015 #2	21	190	130	EVA	fragment	0.68	1.3E-06	1.2E-06
KEOST2014-2015 #2	22	80	50	EVA	fragment	0.63	7.9E-08	7.3E-08
KEOST2014-2015 #2	23	70	60	EVOH	fragment	0.86	9.9E-08	1.1E-07
KEOST2014-2015 #2	24	60	50	EVOH	fragment	0.83	5.9E-08	6.8E-08
KEOST2014-2015 #2	25	30	30	PA	fragment	1.00	1.1E-08	1.2E-08
KEOST2014-2015 #2	26	70	50	PE	fragment	0.71	6.9E-08	6.5E-08
KEOST2014-2015 #2	27	60	40	PE	fragment	0.67	3.8E-08	3.6E-08
KEOST2014-2015 #2	28	50	50	PET	fragment	1.00	4.9E-08	6.6E-08
KEOST2014-2015 #2	29	30	20	PP	fragment	0.67	4.7E-09	4.3E-09
KEOST2014-2015 #3	30	60	60	EVA	fragment	1.00	8.5E-08	7.9E-08
KEOST2014-2015 #3	31	30	20	PE	fragment	0.67	4.7E-09	4.4E-09
KEOST2014-2015 #3	32	50	40	PE	fragment	0.80	3.1E-08	3.0E-08
KEOST2014-2015 #3	33	30	30	PE	fragment	1.00	1.1E-08	1.0E-08
KEOST2014-2015 #3	34	50	40	PE	fragment	0.80	3.1E-08	3.0E-08
KEOST2014-2015 #3	35	60	50	PE	fragment	0.83	5.9E-08	5.6E-08
KEOST2014-2015 #3	36	90	40	PET	fragment	0.44	5.7E-08	7.6E-08
KEOST2014-2015 #3	37	70	50	PET	fragment	0.71	6.9E-08	9.3E-08
KEOST2014-2015 #3	38	90	50	PP	fragment	0.56	8.8E-08	8.0E-08
KEOST2014-2015 #4	39	200	160	EVA	fragment	0.80	2.0E-06	1.9E-06
KEOST2014-2015 #4	40	50	20	EVA	fragment	0.40	7.9E-09	7.3E-09

KEOST2014-2015 #4	41	50	50	EVA	fragment	1.00	4.9E-08	4.6E-08
KEOST2014-2015 #4	42	60	50	EVA	fragment	0.83	5.9E-08	5.5E-08
KEOST2014-2015 #4	43	60	50	PA	fragment	0.83	5.9E-08	6.7E-08
KEOST2014-2015 #4	44	30	20	PE	fragment	0.67	4.7E-09	4.4E-09
KEOST2014-2015 #4	45	100	80	PE	fragment	0.80	2.5E-07	2.4E-07
KEOST2014-2015 #4	46	70	50	PE	fragment	0.71	6.9E-08	6.5E-08
KEOST2014-2015 #4	47	60	30	PE	fragment	0.50	2.1E-08	2.0E-08
KEOST2014-2015 #4	48	40	30	PE	fragment	0.75	1.4E-08	1.3E-08
KEOST2014-2015 #4	49	40	40	PE	fragment	1.00	2.5E-08	2.4E-08
KEOST2014-2015 #4	50	30	30	PE	fragment	1.00	1.1E-08	1.0E-08
KEOST2014-2015 #4	51	40	30	PE	fragment	0.75	1.4E-08	1.3E-08
KEOST2014-2015 #4	52	40	30	PE	fragment	0.75	1.4E-08	1.3E-08
KEOST2014-2015 #4	53	50	40	PE	fragment	0.80	3.1E-08	3.0E-08
KEOST2014-2015 #4	54	90	70	PE	fragment	0.78	1.7E-07	1.6E-07
KEOST2014-2015 #4	55	200	60	PET	fiber	-	2.3E-07	3.1E-07
KEOST2014-2015 #4	56	140	30	PET	fiber	-	4.0E-08	5.3E-08
KEOST2014-2015 #4	57	50	40	PP	fragment	0.80	3.1E-08	2.8E-08
KEOST2014-2015 #4	58	40	20	PS	fragment	0.50	6.3E-09	6.6E-09
KEOST2014-2015 #5	59	30	30	EP	fragment	1.00	1.1E-08	1.4E-08
KEOST2014-2015 #5	60	50	50	EVOH	fragment	1.00	4.9E-08	5.6E-08
KEOST2014-2015 #5	61	60	40	EVOH	fragment	0.67	3.8E-08	4.3E-08
KEOST2014-2015 #5	62	30	20	PA	fragment	0.67	4.7E-09	5.4E-09
KEOST2014-2015 #5	63	60	40	PE	fragment	0.67	3.8E-08	3.6E-08
KEOST2014-2015 #5	64	30	30	PE	fragment	1.00	1.1E-08	1.0E-08
KEOST2014-2015 #5	65	40	30	PE	fragment	0.75	1.4E-08	1.3E-08
KEOST2014-2015 #5	66	100	60	PE	fragment	0.60	1.4E-07	1.3E-07
KEOST2014-2015 #5	67	30	30	PE	fragment	1.00	1.1E-08	1.0E-08
KEOST2014-2015 #5	68	120	70	PE	fragment	0.58	2.3E-07	2.2E-07
KEOST2014-2015 #5	69	50	40	PE	fragment	0.80	3.1E-08	3.0E-08
KEOST2014-2015 #5	70	40	30	PP	fragment	0.75	1.4E-08	1.3E-08
KEOST2014-2015 #6	71	40	20	EVA	fragment	0.50	6.3E-09	5.8E-09
KEOST2014-2015 #6	72	40	30	EVA	fragment	0.75	1.4E-08	1.3E-08
KEOST2014-2015 #6	73	50	50	EVOH	fragment	1.00	4.9E-08	5.6E-08
KEOST2014-2015 #6	74	50	50	EVOH	fragment	1.00	4.9E-08	5.6E-08
KEOST2014-2015 #6	75	60	40	PA	fragment	0.67	3.8E-08	4.3E-08
KEOST2014-2015 #6	76	210	110	PA	fragment	0.52	1.0E-06	1.1E-06
KEOST2014-2015 #6	77	80	70	PA	fragment	0.88	1.5E-07	1.8E-07
KEOST2014-2015 #6	78	30	20	PE	fragment	0.67	4.7E-09	4.4E-09
KEOST2014-2015 #6	79	40	30	PE	fragment	0.75	1.4E-08	1.3E-08
KEOST2014-2015 #6	80	30	30	PE	fragment	1.00	1.1E-08	1.0E-08
KEOST2014-2015 #6	81	40	30	PE	fragment	0.75	1.4E-08	1.3E-08
KEOST2014-2015 #6	82	40	40	PE	fragment	1.00	2.5E-08	2.4E-08
KEOST2014-2015 #6	83	40	20	PE	fragment	0.50	6.3E-09	5.9E-09
KEOST2014-2015 #6	84	30	20	PE	fragment	0.67	4.7E-09	4.4E-09
KEOST2014-2015 #6	85	30	20	PE	fragment	0.67	4.7E-09	4.4E-09
KEOST2014-2015 #6	86	210	30	PET	fiber	-	5.9E-08	8.0E-08

KEOST2014-2015 #6	87	610	40	PP	fiber	-	3.1E-07	2.8E-07
KEOST2014-2015 #6	88	100	60	PVC	fragment	0.60	1.4E-07	2.0E-07
KEOST2014-2015 #7	89	20	20	EVA	fragment	1.00	3.1E-09	2.9E-09
KEOST2014-2015 #7	90	40	40	EVA	fragment	1.00	2.5E-08	2.3E-08
KEOST2014-2015 #7	91	20	20	EVA	fragment	1.00	3.1E-09	2.9E-09
KEOST2014-2015 #7	92	40	40	EVOH	fragment	1.00	2.5E-08	2.9E-08
KEOST2014-2015 #7	93	70	60	EVOH	fragment	0.86	9.9E-08	1.1E-07
KEOST2014-2015 #7	94	50	40	EVOH	fragment	0.80	3.1E-08	3.6E-08
KEOST2014-2015 #7	95	40	40	EVOH	fragment	1.00	2.5E-08	2.9E-08
KEOST2014-2015 #7	96	40	30	PE	fragment	0.75	1.4E-08	1.3E-08
KEOST2014-2015 #7	97	190	100	PE	fragment	0.53	7.5E-07	7.0E-07
KEOST2014-2015 #7	98	50	40	PE	fragment	0.80	3.1E-08	3.0E-08
KEOST2014-2015 #7	99	60	30	PP	fragment	0.50	2.1E-08	1.9E-08
KEOST2014-2015 #8	100	40	40	EVOH	fragment	1.00	2.5E-08	2.9E-08
KEOST2014-2015 #8	101	60	50	EVOH	fragment	0.83	5.9E-08	6.8E-08
KEOST2014-2015 #8	102	80	30	EVOH	fragment	0.38	2.8E-08	3.3E-08
KEOST2014-2015 #8	103	50	30	EVOH	fragment	0.60	1.8E-08	2.0E-08
KEOST2014-2015 #8	104	50	40	EVOH	fragment	0.80	3.1E-08	3.6E-08
KEOST2014-2015 #8	105	30	20	PA	fragment	0.67	4.7E-09	5.4E-09
KEOST2014-2015 #8	106	50	30	PA	fragment	0.60	1.8E-08	2.0E-08
KEOST2014-2015 #8	107	30	30	PE	fragment	1.00	1.1E-08	1.0E-08
KEOST2014-2015 #8	108	50	50	PE	fragment	1.00	4.9E-08	4.6E-08
KEOST2014-2015 #8	109	40	40	PE	fragment	1.00	2.5E-08	2.4E-08
KEOST2014-2015 #8	110	30	20	PP	fragment	0.67	4.7E-09	4.3E-09
KEOST2014-2015 #8	111	60	60	PU	fragment	1.00	8.5E-08	1.0E-07
KEOST2014-2015 #9	112	40	20	EVA	fragment	0.50	6.3E-09	5.8E-09
KEOST2014-2015 #9	113	70	50	PE	fragment	0.71	6.9E-08	6.5E-08
KEOST2014-2015 #9	114	50	50	PE	fragment	1.00	4.9E-08	4.6E-08
KEOST2014-2015 #9	115	40	30	PE	fragment	0.75	1.4E-08	1.3E-08
KEOST2014-2015 #9	116	40	40	PE	fragment	1.00	2.5E-08	2.4E-08
KEOST2014-2015 #9	117	90	60	PE	fragment	0.67	1.3E-07	1.2E-07
KEOST2014-2015 #9	118	40	40	PE	fragment	1.00	2.5E-08	2.4E-08
KEOST2014-2015 #9	119	40	20	PE	fragment	0.50	6.3E-09	5.9E-09
KEOST2014-2015 #9	120	30	30	PE	fragment	1.00	1.1E-08	1.0E-08
KEOST2014-2015 #9	121	80	30	PE	fragment	0.38	2.8E-08	2.7E-08
KEOST2014-2015 #9	122	30	30	PE	fragment	1.00	1.1E-08	1.0E-08
KEOST2014-2015 #9	123	40	30	PP	fragment	0.75	1.4E-08	1.3E-08
KEOST2014-2015 #9	124	40	40	PVC	fragment	1.00	2.5E-08	3.6E-08
KEOST2014-2015 #10	125	60	40	EVA	fragment	0.67	3.8E-08	3.5E-08
KEOST2014-2015 #10	126	60	50	EVOH	fragment	0.83	5.9E-08	6.8E-08
KEOST2014-2015 #10	127	60	50	EVOH	fragment	0.83	5.9E-08	6.8E-08
KEOST2014-2015 #10	128	50	40	EVOH	fragment	0.80	3.1E-08	3.6E-08
KEOST2014-2015 #10	129	50	30	PA	fragment	0.60	1.8E-08	2.0E-08
KEOST2014-2015 #10	130	30	20	PA	fragment	0.67	4.7E-09	5.4E-09
KEOST2014-2015 #10	131	80	50	PC	fragment	0.63	7.9E-08	1.0E-07
KEOST2014-2015 #10	132	50	30	PE	fragment	0.60	1.8E-08	1.7E-08

KEOST2014-2015 #10	133	30	20	PE	fragment	0.67	4.7E-09	4.4E-09
KEOST2014-2015 #10	134	40	40	PE	fragment	1.00	2.5E-08	2.4E-08
KEOST2014-2015 #10	135	100	40	PE	fragment	0.40	6.3E-08	5.9E-08
KEOST2014-2015 #10	136	140	40	PET	fragment	0.29	8.8E-08	1.2E-07
KEOST2014-2015 #10	137	50	40	PP	fragment	0.80	3.1E-08	2.8E-08
KEOST2014-2015 #10	138	30	30	PU	fragment	1.00	1.1E-08	1.2E-08
KEOST2014-2015 #11	139	50	40	EVA	fragment	0.80	3.1E-08	2.9E-08
KEOST2014-2015 #11	140	70	50	EVOH	fragment	0.71	6.9E-08	7.9E-08
KEOST2014-2015 #11	141	50	40	PA	fragment	0.80	3.1E-08	3.6E-08
KEOST2014-2015 #11	142	30	30	PE	fragment	1.00	1.1E-08	1.0E-08
KEOST2014-2015 #11	143	240	40	PE	fiber	-	1.2E-07	1.1E-07
KEOST2014-2015 #11	144	30	30	PE	fragment	1.00	1.1E-08	1.0E-08
KEOST2014-2015 #11	145	130	80	PMMA	fragment	0.62	3.3E-07	3.9E-07
KEOST2014-2015 #11	146	40	40	PMP	fragment	1.00	2.5E-08	2.1E-08
KEOST2014-2015 #11	147	200	60	PVC	fragment	0.30	2.8E-07	4.0E-07
KEOST2014-2015 #11	148	60	50	PVC	fragment	0.83	5.9E-08	8.4E-08
KEOST2014-2015 #12	149	30	30	ABS	fragment	1.00	1.1E-08	1.2E-08
KEOST2014-2015 #12	150	190	190	EP	fragment	1.00	2.7E-06	3.5E-06
KEOST2014-2015 #12	151	470	240	EVA	fragment	0.51	1.1E-05	9.9E-06
KEOST2014-2015 #12	152	80	50	EVOH	fragment	0.63	7.9E-08	9.0E-08
KEOST2014-2015 #12	153	40	30	EVOH	fragment	0.75	1.4E-08	1.6E-08
KEOST2014-2015 #12	154	160	50	PE	fragment	0.31	1.6E-07	1.5E-07
KEOST2014-2015 #12	155	760	30	PET	fiber	-	2.1E-07	2.9E-07
KEOST2014-2015 #12	156	40	30	PU	fragment	0.75	1.4E-08	1.7E-08
KEOST2014-2015 #13	157	300	290	EVA	fragment	0.97	9.9E-06	9.2E-06
KEOST2014-2015 #13	158	50	30	PA	fragment	0.60	1.8E-08	2.0E-08
KEOST2014-2015 #13	159	20	20	PE	fragment	1.00	3.1E-09	3.0E-09
KEOST2014-2015 #13	160	100	70	PE	fragment	0.70	1.9E-07	1.8E-07
KEOST2014-2015 #13	161	50	40	PP	fragment	0.80	3.1E-08	2.8E-08
KEOST2014-2015 #13	162	30	30	PP	fragment	1.00	1.1E-08	9.6E-09
KEOST2014-2015 #13	163	200	30	PP	fiber	-	5.7E-08	5.1E-08
KEOST2014-2015 #13	164	40	30	PP	fragment	0.75	1.4E-08	1.3E-08
KEOST2014-2015 #13	165	90	30	PP	fragment	0.33	3.2E-08	2.9E-08
KEOST2014-2015 #13	166	70	50	PVC	fragment	0.71	6.9E-08	9.8E-08
KEOST2014-2015 #14	167	270	20	EP	fiber	-	3.4E-08	4.4E-08
KEOST2014-2015 #14	168	50	30	EVA	fragment	0.60	1.8E-08	1.6E-08
KEOST2014-2015 #14	169	80	50	EVA	fragment	0.63	7.9E-08	7.3E-08
KEOST2014-2015 #14	170	50	50	EVOH	fragment	1.00	4.9E-08	5.6E-08
KEOST2014-2015 #14	171	50	30	PA	fragment	0.60	1.8E-08	2.0E-08
KEOST2014-2015 #14	172	20	20	PA	fragment	1.00	3.1E-09	3.6E-09
KEOST2014-2015 #14	173	40	40	PE	fragment	1.00	2.5E-08	2.4E-08
KEOST2014-2015 #14	174	80	60	PE	fragment	0.75	1.1E-07	1.1E-07
KEOST2014-2015 #14	175	250	30	PET	fiber	-	7.1E-08	9.5E-08
KEOST2014-2015 #14	176	120	50	PET	fiber	-	9.4E-08	1.3E-07
KEOST2014-2015 #14	177	140	40	PET	fragment	0.29	8.8E-08	1.2E-07
KEOST2014-2015 #14	178	40	30	POM	fragment	0.75	1.4E-08	2.0E-08

KEOST2014-2015 #14	179	100	60	PP	fragment	0.60	1.4E-07	1.3E-07
KEOST2014-2015 #14	180	70	50	PP	fragment	0.71	6.9E-08	6.2E-08
KEOST2014-2015 #14	181	70	50	PU	fragment	0.71	6.9E-08	8.1E-08
KEOST2014-2015 #15	182	80	50	EP	fragment	0.63	7.9E-08	1.0E-07
KEOST2014-2015 #15	183	70	40	EP	fragment	0.57	4.4E-08	5.7E-08
KEOST2014-2015 #15	184	60	60	EVA	fragment	1.00	8.5E-08	7.9E-08
KEOST2014-2015 #15	185	50	50	EVOH	fragment	1.00	4.9E-08	5.6E-08
KEOST2014-2015 #15	186	50	30	PE	fragment	0.60	1.8E-08	1.7E-08
KEOST2014-2015 #15	187	30	30	PE	fragment	1.00	1.1E-08	1.0E-08
KEOST2014-2015 #15	188	40	20	PET	fragment	0.50	6.3E-09	8.5E-09
KEOST2014-2015 #15	189	40	40	PP	fragment	1.00	2.5E-08	2.3E-08
KEOST2014-2015 #16	190	510	30	EP	fiber	-	1.4E-07	1.9E-07
KEOST2014-2015 #16	191	480	180	PA	fragment	0.38	6.1E-06	7.0E-06
KEOST2014-2015 #16	192	50	30	PE	fragment	0.60	1.8E-08	1.7E-08
KEOST2014-2015 #16	193	80	20	POM	fragment	0.25	1.3E-08	1.8E-08
KEOST2014-2015 #16	194	70	30	PP	fragment	0.43	2.5E-08	2.2E-08
KEOST2014-2015 #16	195	60	40	PU	fragment	0.67	3.8E-08	4.4E-08
KEOST2014-2015 #17	196	40	30	EVA	fragment	0.75	1.4E-08	1.3E-08
KEOST2014-2015 #17	197	50	50	EVOH	fragment	1.00	4.9E-08	5.6E-08
KEOST2014-2015 #17	198	40	20	PE	fragment	0.50	6.3E-09	5.9E-09
KEOST2014-2015 #17	199	40	30	PE	fragment	0.75	1.4E-08	1.3E-08
KEOST2014-2015 #17	200	40	30	PE	fragment	0.75	1.4E-08	1.3E-08
KEOST2014-2015 #17	201	450	30	PET	fiber	-	1.3E-07	1.7E-07
KEOST2014-2015 #17	202	800	40	PET	fiber	-	4.0E-07	5.4E-07
KEOST2014-2015 #17	203	350	40	PET	fiber	-	1.8E-07	2.4E-07
KEOST2014-2015 #17	204	100	30	PET	fiber	-	2.8E-08	3.8E-08
KEOST2014-2015 #17	205	150	130	PVC	fragment	0.87	1.0E-06	1.4E-06
KEOST2014-2015 #18	206	50	50	EVOH	fragment	1.00	4.9E-08	5.6E-08
KEOST2014-2015 #18	207	50	40	EVOH	fragment	0.80	3.1E-08	3.6E-08
KEOST2014-2015 #18	208	50	40	PA	fragment	0.80	3.1E-08	3.6E-08
KEOST2014-2015 #18	209	30	30	PE	fragment	1.00	1.1E-08	1.0E-08
KEOST2014-2015 #18	210	30	20	PE	fragment	0.67	4.7E-09	4.4E-09
KEOST2014-2015 #18	211	50	40	PE	fragment	0.80	3.1E-08	3.0E-08
KEOST2014-2015 #18	212	140	30	PE	fiber	-	4.0E-08	3.7E-08
KEOST2014-2015 #19	213	30	20	EP	fragment	0.67	4.7E-09	6.1E-09
KEOST2014-2015 #19	214	200	90	EVA	fragment	0.45	6.4E-07	5.9E-07
KEOST2014-2015 #19	215	60	40	EVOH	fragment	0.67	3.8E-08	4.3E-08
KEOST2014-2015 #19	216	30	30	PA	fragment	1.00	1.1E-08	1.2E-08
KEOST2014-2015 #19	217	40	30	PE	fragment	0.75	1.4E-08	1.3E-08
KEOST2014-2015 #19	218	30	20	PE	fragment	0.67	4.7E-09	4.4E-09
KEOST2014-2015 #19	219	40	40	PE	fragment	1.00	2.5E-08	2.4E-08
KEOST2014-2015 #19	220	30	30	PMP	fragment	1.00	1.1E-08	8.9E-09
KEOST2014-2015 #20	221	60	60	EP	fragment	1.00	8.5E-08	1.1E-07
KEOST2014-2015 #20	222	50	50	EVA	fragment	1.00	4.9E-08	4.6E-08
KEOST2014-2015 #20	223	30	20	PE	fragment	0.67	4.7E-09	4.4E-09
KEOST2014-2015 #20	224	50	40	PE	fragment	0.80	3.1E-08	3.0E-08

KEOST2014-2015 #20	225	40	30	PP	fragment	0.75	1.4E-08	1.3E-08
KEOST2014-2015 #20	226	50	40	PS	fragment	0.80	3.1E-08	3.3E-08
KEOST2014-2015 #20	227	40	30	PU	fragment	0.75	1.4E-08	1.7E-08
KEOST2014-2015 #20	228	40	40	PU	fragment	1.00	2.5E-08	3.0E-08
KEOST2014-2015 #21	229	50	40	ABS	fragment	0.80	3.1E-08	3.5E-08
KEOST2014-2015 #21	230	40	30	EP	fragment	0.75	1.4E-08	1.8E-08
KEOST2014-2015 #21	231	60	50	EVOH	fragment	0.83	5.9E-08	6.8E-08
KEOST2014-2015 #21	232	30	30	EVOH	fragment	1.00	1.1E-08	1.2E-08
KEOST2014-2015 #21	233	80	60	PA	fragment	0.75	1.1E-07	1.3E-07
KEOST2014-2015 #21	234	50	30	PA	fragment	0.60	1.8E-08	2.0E-08
KEOST2014-2015 #21	235	90	70	PC	fragment	0.78	1.7E-07	2.3E-07
KEOST2014-2015 #21	236	60	60	PE	fragment	1.00	8.5E-08	8.0E-08
KEOST2014-2015 #21	237	50	30	PMP	fragment	0.60	1.8E-08	1.5E-08
KEOST2014-2015 #21	238	50	50	PVC	fragment	1.00	4.9E-08	7.0E-08
KEOST2015-2016 #1	239	50	40	ABS	fragment	0.80	3.1E-08	3.5E-08
KEOST2015-2016 #1	240	50	40	EVA	fragment	0.80	3.1E-08	2.9E-08
KEOST2015-2016 #1	241	120	50	EVA	fragment	0.42	1.2E-07	1.1E-07
KEOST2015-2016 #1	242	30	30	PBT	fragment	1.00	1.1E-08	1.4E-08
KEOST2015-2016 #1	243	60	50	PE	fragment	0.83	5.9E-08	5.6E-08
KEOST2015-2016 #1	244	110	100	PE	fragment	0.91	4.3E-07	4.1E-07
KEOST2015-2016 #1	245	80	30	PET	fragment	0.38	2.8E-08	3.8E-08
KEOST2015-2016 #1	246	340	20	PP	fiber	-	4.3E-08	3.9E-08
KEOST2015-2016 #1	247	180	20	PTFE	fiber	-	2.3E-08	4.9E-08
KEOST2015-2016 #1	248	40	40	PVC	fragment	1.00	2.5E-08	3.6E-08
KEOST2015-2016 #2	249	50	40	EP	fragment	0.80	3.1E-08	4.1E-08
KEOST2015-2016 #2	250	50	40	EVA	fragment	0.80	3.1E-08	2.9E-08
KEOST2015-2016 #2	251	170	110	EVA	fragment	0.65	8.1E-07	7.5E-07
KEOST2015-2016 #2	252	60	50	EVA	fragment	0.83	5.9E-08	5.5E-08
KEOST2015-2016 #2	253	180	110	EVA	fragment	0.61	8.6E-07	8.0E-07
KEOST2015-2016 #2	254	50	40	EVOH	fragment	0.80	3.1E-08	3.6E-08
KEOST2015-2016 #2	255	60	30	PE	fragment	0.50	2.1E-08	2.0E-08
KEOST2015-2016 #2	256	60	50	PE	fragment	0.83	5.9E-08	5.6E-08
KEOST2015-2016 #2	257	50	40	PE	fragment	0.80	3.1E-08	3.0E-08
KEOST2015-2016 #2	258	40	30	PET	fragment	0.75	1.4E-08	1.9E-08
KEOST2015-2016 #2	259	170	120	PVC	fragment	0.71	9.6E-07	1.4E-06
KEOST2015-2016 #3	260	50	30	EVOH	fragment	0.60	1.8E-08	2.0E-08
KEOST2015-2016 #3	261	40	30	EVOH	fragment	0.75	1.4E-08	1.6E-08
KEOST2015-2016 #3	262	60	40	PA	fragment	0.67	3.8E-08	4.3E-08
KEOST2015-2016 #3	263	50	40	PBT	fragment	0.80	3.1E-08	4.2E-08
KEOST2015-2016 #3	264	590	40	PET	fiber	-	3.0E-07	4.0E-07
KEOST2015-2016 #3	265	100	40	PET	fragment	0.40	6.3E-08	8.5E-08
KEOST2015-2016 #3	266	250	100	PET	fragment	0.40	9.8E-07	1.3E-06
KEOST2015-2016 #4	267	40	30	PA	fragment	0.75	1.4E-08	1.6E-08
KEOST2015-2016 #4	268	30	20	PA	fragment	0.67	4.7E-09	5.4E-09
KEOST2015-2016 #4	269	70	50	PA	fragment	0.71	6.9E-08	7.8E-08
KEOST2015-2016 #4	270	350	90	PE	fragment	0.26	1.1E-06	1.1E-06

KEOST2015-2016 #4	271	30	20	PE	fragment	0.67	4.7E-09	4.4E-09
KEOST2015-2016 #4	272	40	30	PP	fragment	0.75	1.4E-08	1.3E-08
KEOST2015-2016 #4	273	60	60	PP	fragment	1.00	8.5E-08	7.7E-08
KEOST2015-2016 #4	274	50	40	PP	fragment	0.80	3.1E-08	2.8E-08
KEOST2015-2016 #5	275	80	50	EVA	fragment	0.63	7.9E-08	7.3E-08
KEOST2015-2016 #5	276	50	40	EVOH	fragment	0.80	3.1E-08	3.6E-08
KEOST2015-2016 #5	277	60	50	EVOH	fragment	0.83	5.9E-08	6.8E-08
KEOST2015-2016 #5	278	120	70	PE	fragment	0.58	2.3E-07	2.2E-07
KEOST2015-2016 #5	279	100	40	PE	fragment	0.40	6.3E-08	5.9E-08
KEOST2015-2016 #5	280	60	30	PE	fragment	0.50	2.1E-08	2.0E-08
KEOST2015-2016 #5	281	40	30	PE	fragment	0.75	1.4E-08	1.3E-08
KEOST2015-2016 #5	282	70	40	PE	fragment	0.57	4.4E-08	4.1E-08
KEOST2015-2016 #5	283	50	40	PP	fragment	0.80	3.1E-08	2.8E-08
KEOST2015-2016 #5	284	60	40	PP	fragment	0.67	3.8E-08	3.4E-08
KEOST2015-2016 #6	285	100	80	EP	fragment	0.80	2.5E-07	3.3E-07
KEOST2015-2016 #6	286	30	30	PA	fragment	1.00	1.1E-08	1.2E-08
KEOST2015-2016 #6	287	50	40	PE	fragment	0.80	3.1E-08	3.0E-08
KEOST2015-2016 #6	288	90	50	PE	fragment	0.56	8.8E-08	8.3E-08
KEOST2015-2016 #6	289	60	50	PE	fragment	0.83	5.9E-08	5.6E-08
KEOST2015-2016 #6	290	180	20	PE	fiber	-	2.3E-08	2.1E-08
KEOST2015-2016 #6	291	170	70	PVC	fragment	0.41	3.3E-07	4.7E-07
KEOST2015-2016 #7	292	150	80	EP	fragment	0.53	3.8E-07	4.9E-07
KEOST2015-2016 #7	293	50	40	EVOH	fragment	0.80	3.1E-08	3.6E-08
KEOST2015-2016 #7	294	60	50	EVOH	fragment	0.83	5.9E-08	6.8E-08
KEOST2015-2016 #7	295	50	50	EVOH	fragment	1.00	4.9E-08	5.6E-08
KEOST2015-2016 #7	296	80	50	PA	fragment	0.63	7.9E-08	9.0E-08
KEOST2015-2016 #7	297	30	20	PA	fragment	0.67	4.7E-09	5.4E-09
KEOST2015-2016 #7	298	80	40	PE	fragment	0.50	5.0E-08	4.7E-08
KEOST2015-2016 #7	299	80	60	PE	fragment	0.75	1.1E-07	1.1E-07
KEOST2015-2016 #7	300	220	80	PET	fragment	0.36	5.5E-07	7.5E-07
KEOST2015-2016 #7	301	110	30	PP	fragment	0.27	3.9E-08	3.5E-08
KEOST2015-2016 #7	302	600	20	PVC	fiber	-	7.5E-08	1.1E-07
KEOST2015-2016 #8	303	80	40	EVA	fragment	0.50	5.0E-08	4.7E-08
KEOST2015-2016 #8	304	60	50	PA	fragment	0.83	5.9E-08	6.7E-08
KEOST2015-2016 #8	305	60	40	PE	fragment	0.67	3.8E-08	3.6E-08
KEOST2015-2016 #8	306	260	20	PET	fiber	-	3.3E-08	4.4E-08
KEOST2015-2016 #8	307	390	30	PET	fiber	-	1.1E-07	1.5E-07
KEOST2015-2016 #8	308	40	30	PP	fragment	0.75	1.4E-08	1.3E-08
KEOST2015-2016 #9	309	50	40	EVOH	fragment	0.80	3.1E-08	3.6E-08
KEOST2015-2016 #9	310	40	40	EVOH	fragment	1.00	2.5E-08	2.9E-08
KEOST2015-2016 #9	311	40	20	PA	fragment	0.50	6.3E-09	7.2E-09
KEOST2015-2016 #9	312	50	40	PA	fragment	0.80	3.1E-08	3.6E-08
KEOST2015-2016 #9	313	50	50	PBT	fragment	1.00	4.9E-08	6.6E-08
KEOST2015-2016 #9	314	40	30	PE	fragment	0.75	1.4E-08	1.3E-08
KEOST2015-2016 #9	315	60	50	PE	fragment	0.83	5.9E-08	5.6E-08
KEOST2015-2016 #9	316	40	20	PE	fragment	0.50	6.3E-09	5.9E-09

KEOST2015-2016 #9	317	40	40	PE	fragment	1.00	2.5E-08	2.4E-08
KEOST2015-2016 #9	318	60	50	PE	fragment	0.83	5.9E-08	5.6E-08
KEOST2015-2016 #9	319	250	40	PET	fiber	-	1.3E-07	1.7E-07
KEOST2015-2016 #9	320	50	40	PP	fragment	0.80	3.1E-08	2.8E-08
KEOST2015-2016 #10	321	60	30	EVOH	fragment	0.50	2.1E-08	2.4E-08
KEOST2015-2016 #10	322	50	40	PA	fragment	0.80	3.1E-08	3.6E-08
KEOST2015-2016 #10	323	30	30	PE	fragment	1.00	1.1E-08	1.0E-08
KEOST2015-2016 #10	324	80	60	PE	fragment	0.75	1.1E-07	1.1E-07
KEOST2015-2016 #10	325	50	40	PE	fragment	0.80	3.1E-08	3.0E-08
KEOST2015-2016 #10	326	70	50	PE	fragment	0.71	6.9E-08	6.5E-08
KEOST2015-2016 #10	327	30	30	PET	fragment	1.00	1.1E-08	1.4E-08
KEOST2015-2016 #10	328	40	30	PET	fragment	0.75	1.4E-08	1.9E-08
KEOST2015-2016 #10	329	310	20	PU	fiber	-	3.9E-08	4.6E-08
KEOST2015-2016 #11	330	30	20	PA	fragment	0.67	4.7E-09	5.4E-09
KEOST2015-2016 #11	331	60	50	PA	fragment	0.83	5.9E-08	6.7E-08
KEOST2015-2016 #11	332	40	30	PA	fragment	0.75	1.4E-08	1.6E-08
KEOST2015-2016 #11	333	50	40	PA	fragment	0.80	3.1E-08	3.6E-08
KEOST2015-2016 #11	334	30	30	PA	fragment	1.00	1.1E-08	1.2E-08
KEOST2015-2016 #11	335	40	30	PE	fragment	0.75	1.4E-08	1.3E-08
KEOST2015-2016 #11	336	40	20	PE	fragment	0.50	6.3E-09	5.9E-09
KEOST2015-2016 #11	337	60	60	PE	fragment	1.00	8.5E-08	8.0E-08
KEOST2015-2016 #11	338	50	30	PE	fragment	0.60	1.8E-08	1.7E-08
KEOST2015-2016 #11	339	400	30	PET	fiber	-	1.1E-07	1.5E-07
KEOST2015-2016 #11	340	240	100	PET	fragment	0.42	9.4E-07	1.3E-06
KEOST2015-2016 #11	341	110	40	PP	fragment	0.36	6.9E-08	6.3E-08
KEOST2015-2016 #12	342	140	30	ABS	fragment	0.21	4.9E-08	5.5E-08
KEOST2015-2016 #12	343	70	50	EVA	fragment	0.71	6.9E-08	6.4E-08
KEOST2015-2016 #12	344	40	30	PE	fragment	0.75	1.4E-08	1.3E-08
KEOST2015-2016 #12	345	160	20	PE	fiber	-	2.0E-08	1.9E-08
KEOST2015-2016 #12	346	60	60	PE	fragment	1.00	8.5E-08	8.0E-08
KEOST2015-2016 #12	347	50	30	PE	fragment	0.60	1.8E-08	1.7E-08
KEOST2015-2016 #12	348	60	50	PET	fragment	0.83	5.9E-08	8.0E-08
KEOST2015-2016 #12	349	80	30	PET	fiber	-	2.3E-08	3.1E-08
KEOST2015-2016 #13	350	110	70	EVA	fragment	0.64	2.1E-07	2.0E-07
KEOST2015-2016 #13	351	120	60	PA	fragment	0.50	1.7E-07	1.9E-07
KEOST2015-2016 #13	352	110	20	PE	fiber	-	1.4E-08	1.3E-08
KEOST2015-2016 #13	353	50	40	PE	fragment	0.80	3.1E-08	3.0E-08
KEOST2015-2016 #13	354	100	20	PET	fiber	-	1.3E-08	1.7E-08
KEOST2015-2016 #13	355	190	40	PET	fragment	0.21	1.2E-07	1.6E-07
KEOST2015-2016 #13	356	50	30	PP	fragment	0.60	1.8E-08	1.6E-08
KEOST2015-2016 #14	357	40	30	ABS	fragment	0.75	1.4E-08	1.6E-08
KEOST2015-2016 #14	358	160	90	EVA	fragment	0.56	5.1E-07	4.7E-07
KEOST2015-2016 #14	359	50	40	PE	fragment	0.80	3.1E-08	3.0E-08
KEOST2015-2016 #14	360	400	30	PET	fiber	-	1.1E-07	1.5E-07
KEOST2015-2016 #14	361	30	30	PP	fragment	1.00	1.1E-08	9.6E-09
KEOST2015-2016 #15	362	30	30	PA	fragment	1.00	1.1E-08	1.2E-08

KEOST2015-2016 #15	363	30	30	PA	fragment	1.00	1.1E-08	1.2E-08
KEOST2015-2016 #15	364	50	50	PE	fragment	1.00	4.9E-08	4.6E-08
KEOST2015-2016 #15	365	50	50	PE	fragment	1.00	4.9E-08	4.6E-08
KEOST2015-2016 #15	366	40	40	PVC	fragment	1.00	2.5E-08	3.6E-08
KEOST2015-2016 #16	367	50	30	EVOH	fragment	0.60	1.8E-08	2.0E-08
KEOST2015-2016 #16	368	100	40	PA	fragment	0.40	6.3E-08	7.2E-08
KEOST2015-2016 #16	369	90	40	PBT	fragment	0.44	5.7E-08	7.6E-08
KEOST2015-2016 #16	370	240	30	PET	fiber	-	6.8E-08	9.2E-08
KEOST2015-2016 #16	371	420	40	PET	fiber	-	2.1E-07	2.9E-07
KEOST2015-2016 #16	372	40	30	PP	fragment	0.75	1.4E-08	1.3E-08
KEOST2015-2016 #16	373	130	40	PTFE	fragment	0.31	8.2E-08	1.8E-07
KEOST2016-2017 #1	381	50	40	EVA	fragment	0.80	3.1E-08	2.9E-08
KEOST2016-2017 #1	382	50	20	PBT	fragment	0.40	7.9E-09	1.1E-08
KEOST2016-2017 #1	383	60	50	PE	fragment	0.83	5.9E-08	5.6E-08
KEOST2016-2017 #1	384	70	50	PE	fragment	0.71	6.9E-08	6.5E-08
KEOST2016-2017 #1	385	50	40	PE	fragment	0.80	3.1E-08	3.0E-08
KEOST2016-2017 #1	386	60	50	PE	fragment	0.83	5.9E-08	5.6E-08
KEOST2016-2017 #1	387	30	30	PET	fragment	1.00	1.1E-08	1.4E-08
KEOST2016-2017 #1	388	30	30	PET	fragment	1.00	1.1E-08	1.4E-08
KEOST2016-2017 #1	389	150	20	PMP	fiber	-	1.9E-08	1.6E-08
KEOST2016-2017 #2	390	100	10	EP	fiber	-	3.1E-09	4.1E-09
KEOST2016-2017 #2	391	70	60	EVOH	fragment	0.86	9.9E-08	1.1E-07
KEOST2016-2017 #2	392	200	200	PE	fragment	1.00	3.1E-06	3.0E-06
KEOST2016-2017 #2	393	120	30	PE	fiber	-	3.4E-08	3.2E-08
KEOST2016-2017 #2	394	30	30	PP	fragment	1.00	1.1E-08	9.6E-09
KEOST2016-2017 #2	395	120	20	PVC	fiber	-	1.5E-08	2.1E-08
KEOST2016-2017 #2	396	90	50	PVC	fragment	0.56	8.8E-08	1.3E-07
KEOST2016-2017 #3	397	50	20	EP	fiber	-	6.3E-09	8.2E-09
KEOST2016-2017 #3	398	60	50	EVOH	fragment	0.83	5.9E-08	6.8E-08
KEOST2016-2017 #3	399	50	30	PE	fragment	0.60	1.8E-08	1.7E-08
KEOST2016-2017 #3	400	50	50	PE	fragment	1.00	4.9E-08	4.6E-08
KEOST2016-2017 #3	401	40	30	PE	fragment	0.75	1.4E-08	1.3E-08
KEOST2016-2017 #3	402	60	40	PE	fragment	0.67	3.8E-08	3.6E-08
KEOST2016-2017 #3	403	70	60	PE	fragment	0.86	9.9E-08	9.3E-08
KEOST2016-2017 #3	404	60	50	PET	fragment	0.83	5.9E-08	8.0E-08
KEOST2016-2017 #3	405	40	40	PVC	fragment	1.00	2.5E-08	3.6E-08
KEOST2016-2017 #4	406	40	30	EVOH	fragment	0.75	1.4E-08	1.6E-08
KEOST2016-2017 #4	407	70	50	EVOH	fragment	0.71	6.9E-08	7.9E-08
KEOST2016-2017 #4	408	50	50	PE	fragment	1.00	4.9E-08	4.6E-08
KEOST2016-2017 #4	409	30	30	PE	fragment	1.00	1.1E-08	1.0E-08
KEOST2016-2017 #4	410	30	30	PE	fragment	1.00	1.1E-08	1.0E-08
KEOST2016-2017 #4	411	40	30	PP	fragment	0.75	1.4E-08	1.3E-08
KEOST2016-2017 #4	412	80	50	PP	fragment	0.63	7.9E-08	7.1E-08
Blank A	413	40	30	PET	fragment	0.75	1.4E-08	1.9E-08
Blank A	414	150	40	PET	fragment	0.27	9.4E-08	1.3E-07

Table S9. Microplastic abundance fluxes at Stn. KEO. Polymer-type abbreviations are as shown in Table S1

Sample No.		Open date	Close date	Sampling interval (days)	Aliquot size	Fixed volume (mL)	Analyzed sample volume (mL)	Dilution rate	Abundance flux (pieces m^{-2} day^{-1})																	
									ABS	EP	EVA	EVOH	PA	PBT	PC	PE	PET	PMMA	PMP	POM	PP	PS	PTFE	PU	PVC	Total MPs
KEOST2014-2015	1	2014/7/1	2014/7/19	18	1/10	50	5.0	10	11	0	0	0	22	11	0	56	33	0	0	0	22	0	0	0	0	156
KEOST2014-2015	2	2014/7/19	2014/8/6	18	1/10	50	2.0	25	0	28	56	56	28	0	0	56	28	0	0	0	28	0	0	0	0	278
KEOST2014-2015	3	2014/8/6	2014/8/24	18	1/10	50	2.0	25	0	0	28	0	0	0	0	139	56	0	0	0	28	0	0	0	0	250
KEOST2014-2015	4	2014/8/24	2014/9/11	18	1/10	50	2.0	25	0	0	111	0	28	0	0	306	0	0	0	0	28	28	0	0	0	500
KEOST2014-2015	5	2014/9/11	2014/9/29	18	1/10	50	2.0	25	0	28	0	56	28	0	0	194	0	0	0	0	28	0	0	0	0	333
KEOST2014-2015	6	2014/9/29	2014/10/17	18	1/10	50	2.0	25	0	0	56	56	83	0	0	222	0	0	0	0	0	0	0	0	28	444
KEOST2014-2015	7	2014/10/17	2014/11/4	18	1/10	50	2.0	25	0	0	83	111	0	0	0	83	0	0	0	0	28	0	0	0	0	306
KEOST2014-2015	8	2014/11/4	2014/11/22	18	1/10	50	2.0	25	0	0	0	139	56	0	0	83	0	0	0	0	28	0	0	28	0	333
KEOST2014-2015	9	2014/11/22	2014/12/10	18	1/10	50	2.0	25	0	0	28	0	0	0	0	278	0	0	0	0	28	0	0	0	28	361
KEOST2014-2015	10	2014/12/10	2014/12/28	18	1/10	50	2.0	25	0	0	28	83	56	0	28	111	28	0	0	0	28	0	0	28	0	389
KEOST2014-2015	11	2014/12/28	2015/1/15	18	1/10	50	2.0	25	0	0	28	28	28	0	0	56	0	28	28	0	0	0	0	0	56	250
KEOST2014-2015	12	2015/1/15	2015/2/2	18	1/10	50	2.0	25	28	28	28	56	0	0	0	28	0	0	0	0	0	0	0	28	0	194
KEOST2014-2015	13	2015/2/2	2015/2/20	18	1/10	50	2.0	25	0	0	28	0	28	0	0	56	0	0	0	0	111	0	0	0	28	250
KEOST2014-2015	14	2015/2/20	2015/3/10	18	1/10	50	2.0	25	0	0	56	28	56	0	0	56	28	0	0	28	56	0	0	28	0	333
KEOST2014-2015	15	2015/3/10	2015/3/28	18	1/10	50	2.0	25	0	56	28	28	0	0	0	56	28	0	0	0	28	0	0	0	0	222
KEOST2014-2015	16	2015/3/28	2015/4/15	18	1/10	50	2.0	25	0	0	0	0	28	0	0	28	0	0	0	28	28	0	0	28	0	139
KEOST2014-2015	17	2015/4/15	2015/5/3	18	1/10	50	0.5	100	0	0	111	111	0	0	0	333	0	0	0	0	0	0	0	0	111	667
KEOST2014-2015	18	2015/5/3	2015/5/21	18	1/10	50	0.5	100	0	0	0	222	111	0	0	333	0	0	0	0	0	0	0	0	0	667
KEOST2014-2015	19	2015/5/21	2015/6/8	18	1/10	50	0.5	100	0	111	111	111	111	0	0	333	0	0	111	0	0	0	0	0	0	889
KEOST2014-2015	20	2015/6/8	2015/6/26	18	1/10	50	0.5	100	0	111	111	0	0	0	0	222	0	0	0	0	111	111	0	222	0	889
KEOST2014-2015	21	2015/6/26	2015/7/14	18	1/10	50	1.0	50	56	56	0	111	111	0	56	56	0	0	56	0	0	0	0	0	56	556
KEOST2015-2016	1	2015/9/10	2015/9/28	18	1/10	50	2.0	25	28	0	56	0	0	28	0	56	28	0	0	0	0	0	0	0	28	222
KEOST2015-2016	2	2015/9/28	2015/10/16	18	1/10	50	2.0	25	0	28	111	28	0	0	0	83	28	0	0	0	0	0	0	0	28	306
KEOST2015-2016	3	2015/10/16	2015/11/3	18	1/10	50	2.0	25	0	0	0	56	28	28	0	0	56	0	0	0	0	0	0	0	0	167
KEOST2015-2016	4	2015/11/3	2015/11/21	18	1/10	50	2.0	25	0	0	0	0	83	0	0	56	0	0	0	0	83	0	0	0	0	222
KEOST2015-2016	5	2015/11/21	2015/12/9	18	1/10	50	2.0	25	0	0	28	56	0	0	0	139	0	0	0	0	56	0	0	0	0	278
KEOST2015-2016	6	2015/12/9	2015/12/27	18	1/10	50	1.0	50	0	56	0	0	56	0	0	167	0	0	0	0	0	0	0	0	56	333
KEOST2015-2016	7	2015/12/27	2016/1/14	18	1/10	50	2.0	25	0	28	0	83	56	0	0	56	28	0	0	0	28	0	0	0	0	278
KEOST2015-2016	8	2016/1/14	2016/2/1	18	1/10	50	2.0	25	0	0	28	0	28	0	0	28	0	0	0	0	28	0	0	0	0	111
KEOST2015-2016	9	2016/2/1	2016/2/19	18	1/10	50	2.0	25	0	0	0	56	56	28	0	139	0	0	0	0	28	0	0	0	0	306
KEOST2015-2016	10	2016/2/19	2016/3/8	18	1/10	50	2.0	25	0	0	0	28	28	0	0	111	56	0	0	0	0	0	0	0	0	222
KEOST2015-2016	11	2016/3/8	2016/3/26	18	1/10	50	2.0	25	0	0	0	0	139	0	0	111	28	0	0	0	28	0	0	0	0	306
KEOST2015-2016	12	2016/3/26	2016/4/13	18	1/10	50	0.5	100	111	0	111	0	0	0	0	333	111	0	0	0	0	0	0	0	0	667
KEOST2015-2016	13	2016/4/13	2016/5/1	18	1/10	50	1.0	50	0	0	56	0	56	0	0	56	56	0	0	0	56	0	0	0	0	278
KEOST2015-2016	14	2016/5/1	2016/5/19	18	1/10	50	2.0	25	28	0	28	0	0	0	0	28	0	0	0	0	28	0	0	0	0	111
KEOST2015-2016	15	2016/5/19	2016/6/6	18	1/10	50	1.0	50	0	0	0	0	111	0	0	111	0	0	0	0	0	0	0	0	56	278
KEOST2015-2016	16	2016/6/6	2016/6/24	18	1/10	50	1.0	50	0	0	0	56	56	56	0	0	0	0	0	0	56	0	56	0	0	278
KEOST2016-2017	1	2016/7/10	2016/7/31	21	1/10	50	0.5	100	0	0	95	0	0	95	0	381	190	0	0	0	0	0	0	0	0	762
KEOST2016-2017	2	2016/7/31	2016/8/21	21	1/10	50	1.0	50	0	0	0	48	0	0	0	48	0	0	0	0	48	0	0	0	48	190
KEOST2016-2017	3	2016/8/21	2016/9/11	21	1/10	50	1.0	50	0	0	0	48	0	0	0	238	48	0	0	0	0	0	0	0	48	381
KEOST2016-2017	4	2016/9/11	2016/10/2	21	1/10	50	1.0	50	0	0	0	95	0	0	0	143	0	0	0	0	95	0	0	0	0	333

Table S10. Mean polymer-type composition by abundance, mass, and carbon fluxes of microplastics at Stn. KEO. Polymer-type abbreviations are as shown in Table S1

Abundance flux		Mass flux		Carbon flux	
Polymer-type	%	Polymer-type	%	Polymer-type	%
PE	37.4	EVA	35.3	EVA	34.2
EVOH	12.1	PE	18.7	PE	24.6
PA	10.0	PA	12.6	PA	12.3
EVA	9.6	PVC	11.6	PVC	6.9
PP	7.7	EP	6.8	EP	6.7
PET	5.9	PET	6.3	PET	6.0
PVC	4.0	EVOH	4.0	EVOH	4.1
EP	3.6	PP	1.4	PP	1.9
PU	2.5	PC	0.7	PC	0.7
ABS	1.8	PU	0.5	PMMA	0.6
PBT	1.8	PMMA	0.5	ABS	0.6
PMP	1.3	ABS	0.4	PBT	0.4
PS	0.9	PBT	0.4	PU	0.3
PC	0.6	PTFE	0.4	PS	0.2
POM	0.4	PS	0.2	PTFE	0.2
PTFE	0.4	PMP	0.1	PMP	0.1
PMMA	0.2	POM	0.05	POM	0.03

Table S11. Mean size composition by abundance and mass fluxes of microplastics at Stn. KEO during the sampling period

Size range	Abundance flux (%)	Mass flux (%)
20-50	61.7	8.6
50-100	28.4	15.5
100-150	4.6	15.9
150-200	3.8	21.1
200-300	0.9	16.9
>300	0.6	22.1

Table S12. Microplastic mass fluxes at Stn. KEO. Polymer-type abbreviations are as shown in Table S1

Sample No.		Open date	Close date	Mass flux (mg m^{-2} day^{-1})																	
				ABS	EP	EVA	EVOH	PA	PBT	PC	PE	PET	PMMA	PMP	POM	PP	PS	PTFE	PU	PVC	Total
KEOST2014-2015	1	2014/7/1	2014/7/19	1.3E-04	0.0E+00	0.0E+00	0.0E+00	3.6E-04	1.0E-03	0.0E+00	4.2E-03	4.2E-03	0.0E+00	0.0E+00	0.0E+00	2.8E-04	0.0E+00	0.0E+00	5.9E-15	0.0E+00	1.0E-02
KEOST2014-2015	2	2014/7/19	2014/8/6	0.0E+00	2.8E-03	3.5E-02	5.0E-03	3.4E-04	0.0E+00	0.0E+00	2.8E-03	1.8E-03	0.0E+00	0.0E+00	0.0E+00	1.2E-04	0.0E+00	0.0E+00	0.0E+00	0.0E+00	4.8E-02
KEOST2014-2015	3	2014/8/6	2014/8/24	0.0E+00	0.0E+00	2.2E-03	0.0E+00	0.0E+00	0.0E+00	0.0E+00	3.6E-03	4.7E-03	0.0E+00	0.0E+00	0.0E+00	2.2E-03	0.0E+00	0.0E+00	0.0E+00	0.0E+00	1.3E-02
KEOST2014-2015	4	2014/8/24	2014/9/11	0.0E+00	0.0E+00	5.5E-02	0.0E+00	1.9E-03	0.0E+00	0.0E+00	1.6E-02	1.0E-14	0.0E+00	0.0E+00	0.0E+00	7.9E-04	1.8E-04	0.0E+00	0.0E+00	0.0E+00	7.4E-02
KEOST2014-2015	5	2014/9/11	2014/9/29	0.0E+00	3.8E-04	0.0E+00	2.8E-03	1.5E-04	0.0E+00	0.0E+00	1.2E-02	0.0E+00	0.0E+00	0.0E+00	0.0E+00	3.6E-04	0.0E+00	0.0E+00	0.0E+00	0.0E+00	1.6E-02
KEOST2014-2015	6	2014/9/29	2014/10/17	0.0E+00	0.0E+00	5.3E-04	3.1E-03	3.8E-02	0.0E+00	0.0E+00	2.2E-03	2.2E-15	0.0E+00	0.0E+00	0.0E+00	7.7E-15	0.0E+00	0.0E+00	0.0E+00	5.6E-03	4.9E-02
KEOST2014-2015	7	2014/10/17	2014/11/4	0.0E+00	0.0E+00	8.1E-04	5.8E-03	0.0E+00	0.0E+00	0.0E+00	2.1E-02	0.0E+00	0.0E+00	0.0E+00	0.0E+00	5.3E-04	0.0E+00	0.0E+00	0.0E+00	0.0E+00	2.8E-02
KEOST2014-2015	8	2014/11/4	2014/11/22	0.0E+00	0.0E+00	0.0E+00	5.2E-03	7.1E-04	0.0E+00	0.0E+00	2.2E-03	0.0E+00	0.0E+00	0.0E+00	0.0E+00	1.2E-04	0.0E+00	0.0E+00	2.8E-03	0.0E+00	1.1E-02
KEOST2014-2015	9	2014/11/22	2014/12/10	0.0E+00	0.0E+00	1.6E-04	0.0E+00	0.0E+00	0.0E+00	0.0E+00	9.6E-03	0.0E+00	0.0E+00	0.0E+00	0.0E+00	3.6E-04	0.0E+00	0.0E+00	0.0E+00	9.9E-04	1.1E-02
KEOST2014-2015	10	2014/12/10	2014/12/28	0.0E+00	0.0E+00	9.7E-04	4.8E-03	7.1E-04	0.0E+00	2.9E-03	2.9E-03	3.3E-03	0.0E+00	0.0E+00	0.0E+00	7.9E-04	0.0E+00	0.0E+00	3.5E-04	0.0E+00	1.7E-02
KEOST2014-2015	11	2014/12/28	2015/1/15	0.0E+00	0.0E+00	8.1E-04	2.2E-03	9.9E-04	0.0E+00	0.0E+00	5.6E-04	0.0E+00	1.1E-02	5.8E-04	0.0E+00	0.0E+00	0.0E+00	0.0E+00	0.0E+00	1.4E-02	2.9E-02
KEOST2014-2015	12	2015/1/15	2015/2/2	3.3E-04	9.7E-02	2.7E-01	3.0E-03	0.0E+00	0.0E+00	0.0E+00	4.1E-03	8.1E-15	0.0E+00	0.0E+00	0.0E+00	0.0E+00	0.0E+00	0.0E+00	4.6E-04	0.0E+00	3.8E-01
KEOST2014-2015	13	2015/2/2	2015/2/20	0.0E+00	0.0E+00	2.6E-01	0.0E+00	5.6E-04	0.0E+00	0.0E+00	5.1E-03	0.0E+00	0.0E+00	0.0E+00	0.0E+00	2.2E-03	0.0E+00	0.0E+00	0.0E+00	2.7E-03	2.7E-01
KEOST2014-2015	14	2015/2/20	2015/3/10	0.0E+00	1.2E-15	2.5E-03	1.6E-03	6.6E-04	0.0E+00	0.0E+00	3.6E-03	3.3E-03	0.0E+00	0.0E+00	5.6E-04	5.3E-03	0.0E+00	0.0E+00	2.2E-03	0.0E+00	2.0E-02
KEOST2014-2015	15	2015/3/10	2015/3/28	0.0E+00	4.4E-03	2.2E-03	1.6E-03	0.0E+00	0.0E+00	0.0E+00	7.4E-04	2.4E-04	0.0E+00	0.0E+00	0.0E+00	6.3E-04	0.0E+00	0.0E+00	0.0E+00	0.0E+00	9.8E-03
KEOST2014-2015	16	2015/3/28	2015/4/15	0.0E+00	5.2E-15	0.0E+00	0.0E+00	1.9E-01	0.0E+00	0.0E+00	4.6E-04	0.0E+00	0.0E+00	0.0E+00	4.9E-04	6.2E-04	0.0E+00	0.0E+00	1.2E-03	0.0E+00	2.0E-01
KEOST2014-2015	17	2015/4/15	2015/5/3	0.0E+00	0.0E+00	1.5E-03	6.3E-03	0.0E+00	0.0E+00	0.0E+00	3.6E-03	1.1E-13	0.0E+00	0.0E+00	0.0E+00	0.0E+00	0.0E+00	0.0E+00	0.0E+00	1.6E-01	1.7E-01
KEOST2014-2015	18	2015/5/3	2015/5/21	0.0E+00	0.0E+00	0.0E+00	1.0E-02	4.0E-03	0.0E+00	0.0E+00	4.9E-03	0.0E+00	0.0E+00	0.0E+00	0.0E+00	0.0E+00	0.0E+00	0.0E+00	0.0E+00	0.0E+00	1.9E-02
KEOST2014-2015	19	2015/5/21	2015/6/8	0.0E+00	6.8E-04	6.6E-02	4.8E-03	1.3E-03	0.0E+00	0.0E+00	4.6E-03	0.0E+00	0.0E+00	9.9E-04	0.0E+00	0.0E+00	0.0E+00	0.0E+00	0.0E+00	0.0E+00	7.8E-02
KEOST2014-2015	20	2015/6/8	2015/6/26	0.0E+00	1.2E-02	5.1E-03	0.0E+00	0.0E+00	0.0E+00	0.0E+00	3.8E-03	0.0E+00	0.0E+00	0.0E+00	0.0E+00	1.4E-03	3.6E-03	0.0E+00	5.1E-03	0.0E+00	3.1E-02
KEOST2014-2015	21	2015/6/26	2015/7/14	1.9E-03	1.0E-03	0.0E+00	4.4E-03	8.3E-03	0.0E+00	1.3E-02	4.4E-03	0.0E+00	0.0E+00	8.2E-04	0.0E+00	0.0E+00	0.0E+00	0.0E+00	0.0E+00	3.9E-03	3.8E-02
KEOST2015-2016	1	2015/9/10	2015/9/28	9.7E-04	0.0E+00	3.9E-03	0.0E+00	0.0E+00	4.0E-04	0.0E+00	1.3E-02	1.1E-03	0.0E+00	0.0E+00	0.0E+00	1.1E-15	0.0E+00	1.4E-15	0.0E+00	9.9E-04	2.0E-02
KEOST2015-2016	2	2015/9/28	2015/10/16	0.0E+00	1.1E-03	4.5E-02	1.0E-03	0.0E+00	0.0E+00	0.0E+00	2.9E-03	5.3E-04	0.0E+00	0.0E+00	0.0E+00	0.0E+00	0.0E+00	0.0E+00	0.0E+00	3.8E-02	8.9E-02
KEOST2015-2016	3	2015/10/16	2015/11/3	0.0E+00	0.0E+00	0.0E+00	1.0E-03	1.2E-03	1.2E-03	0.0E+00	0.0E+00	3.9E-02	0.0E+00	0.0E+00	0.0E+00	0.0E+00	0.0E+00	0.0E+00	0.0E+00	0.0E+00	4.3E-02
KEOST2015-2016	4	2015/11/3	2015/11/21	0.0E+00	0.0E+00	0.0E+00	0.0E+00	2.8E-03	0.0E+00	0.0E+00	2.9E-02	0.0E+00	0.0E+00	0.0E+00	0.0E+00	3.3E-03	0.0E+00	0.0E+00	0.0E+00	0.0E+00	3.5E-02
KEOST2015-2016	5	2015/11/21	2015/12/9	0.0E+00	0.0E+00	2.0E-03	2.9E-03	0.0E+00	0.0E+00	0.0E+00	9.8E-03	0.0E+00	0.0E+00	0.0E+00	0.0E+00	1.7E-03	0.0E+00	0.0E+00	0.0E+00	0.0E+00	1.6E-02
KEOST2015-2016	6	2015/12/9	2015/12/27	0.0E+00	1.8E-02	0.0E+00	0.0E+00	6.7E-04	0.0E+00	0.0E+00	9.4E-03	0.0E+00	0.0E+00	0.0E+00	0.0E+00	0.0E+00	0.0E+00	0.0E+00	0.0E+00	2.6E-02	5.4E-02
KEOST2015-2016	7	2015/12/27	2016/1/14	0.0E+00	1.4E-02	0.0E+00	4.5E-03	2.6E-03	0.0E+00	0.0E+00	4.3E-03	2.1E-02	0.0E+00	0.0E+00	0.0E+00	9.8E-04	0.0E+00	0.0E+00	0.0E+00	3.0E-15	4.7E-02
KEOST2015-2016	8	2016/1/14	2016/2/1	0.0E+00	0.0E+00	1.3E-03	0.0E+00	1.9E-03	0.0E+00	0.0E+00	9.9E-04	5.4E-15	0.0E+00	0.0E+00	0.0E+00	3.6E-04	0.0E+00	0.0E+00	0.0E+00	0.0E+00	4.5E-03
KEOST2015-2016	9	2016/2/1	2016/2/19	0.0E+00	0.0E+00	0.0E+00	1.8E-03	1.2E-03	1.8E-03	0.0E+00	4.3E-03	4.7E-15	0.0E+00	0.0E+00	0.0E+00	7.9E-04	0.0E+00	0.0E+00	0.0E+00	0.0E+00	9.9E-03
KEOST2015-2016	10	2016/2/19	2016/3/8	0.0E+00	0.0E+00	0.0E+00	6.8E-04	9.9E-04	0.0E+00	0.0E+00	5.9E-03	9.3E-04	0.0E+00	0.0E+00	0.0E+00	0.0E+00	0.0E+00	0.0E+00	1.3E-15	0.0E+00	8.5E-03
KEOST2015-2016	11	2016/3/8	2016/3/26	0.0E+00	0.0E+00	0.0E+00	0.0E+00	3.8E-03	0.0E+00	0.0E+00	3.2E-03	3.5E-02	0.0E+00	0.0E+00	0.0E+00	1.7E-03	0.0E+00	0.0E+00	0.0E+00	0.0E+00	4.4E-02
KEOST2015-2016	12	2016/3/26	2016/4/13	6.1E-03	0.0E+00	7.1E-03	0.0E+00	0.0E+00	0.0E+00	0.0E+00	1.2E-02	8.8E-03	0.0E+00	0.0E+00	0.0E+00	0.0E+00	0.0E+00	0.0E+00	0.0E+00	0.0E+00	3.4E-02
KEOST2015-2016	13	2016/4/13	2016/5/1	0.0E+00	0.0E+00	1.1E-02	0.0E+00	1.1E-02	0.0E+00	0.0E+00	1.6E-03	9.0E-03	0.0E+00	0.0E+00	0.0E+00	8.9E-04	0.0E+00	0.0E+00	0.0E+00	0.0E+00	3.3E-02
KEOST2015-2016	14	2016/5/1	2016/5/19	4.4E-04	0.0E+00	1.3E-02	0.0E+00	0.0E+00	0.0E+00	0.0E+00	8.2E-04	4.2E-15	0.0E+00	0.0E+00	0.0E+00	2.7E-04	0.0E+00	0.0E+00	0.0E+00	0.0E+00	1.5E-02
KEOST2015-2016	15	2016/5/19	2016/6/6	0.0E+00	0.0E+00	0.0E+00	0.0E+00	1.3E-03	0.0E+00	0.0E+00	5.1E-03	0.0E+00	0.0E+00	0.0E+00	0.0E+00	0.0E+00	0.0E+00	0.0E+00	0.0E+00	2.0E-03	8.5E-03
KEOST2015-2016	16	2016/6/6	2016/6/24	0.0E+00	0.0E+00	0.0E+00	1.1E-03	4.0E-03	4.2E-03	0.0E+00	0.0E+00	2.1E-14	0.0E+00	0.0E+00	0.0E+00	7.1E-04	0.0E+00	9.8E-03	0.0E+00	0.0E+00	2.0E-02
KEOST2016-2017	1	2016/7/10	2016/7/31	0.0E+00	0.0E+00	2.8E-03	0.0E+00	0.0E+00	1.0E-03	0.0E+00	2.0E-02	2.7E-03	0.0E+00	1.5E-15	0.0E+00	0.0E+00	0.0E+00	0.0E+00	0.0E+00	0.0E+00	2.6E-02
KEOST2016-2017	2	2016/7/31	2016/8/21	0.0E+00	1.9E-16	0.0E+00	5.4E-03	0.0E+00	0.0E+00	0.0E+00	1.4E-01	0.0E+00	0.0E+00	0.0E+00	0.0E+00	4.6E-04	0.0E+00	0.0E+00	0.0E+00	6.0E-03	1.5E-01
KEOST2016-2017	3	2016/8/21	2016/9/11	0.0E+00	3.9E-16	0.0E+00	3.2E-03	0.0E+00	0.0E+00	0.0E+00	9.8E-03	3.8E-03	0.0E+00	0.0E+00	0.0E+00	0.0E+00	0.0E+00	0.0E+00	0.0E+00	1.7E-03	1.8E-02
KEOST2016-2017	4	2016/9/11	2016/10/2	0.0E+00	0.0E+00	0.0E+00	4.5E-03	0.0E+00	0.0E+00	0.0E+00	3.2E-03	0.0E+00	0.0E+00	0.0E+00	0.0E+00	4.0E-03	0.0E+00	0.0E+00	0.0E+00	0.0E+00	1.2E-02

Table S13. Microplastic carbon fluxes at Stn. KEO. Polymer-type abbreviations are as shown in Table S1

Sample No.		Open date	Close date	Carbon flux (mg m^{-2} day^{-1})																	
				ABS	EP	EVA	EVOH	PA	PBT	PC	PE	PET	PMMA	PMP	POM	PP	PS	PTFE	PU	PVC	Total
KEOST2014-2015	1	2014/7/1	2014/7/19	1.1E-04	0.0E+00	0.0E+00	0.0E+00	2.3E-04	6.7E-04	0.0E+00	3.6E-03	2.7E-03	0.0E+00	0.0E+00	0.0E+00	2.4E-04	0.0E+00	0.0E+00	2.4E-15	0.0E+00	7.5E-03
KEOST2014-2015	2	2014/7/19	2014/8/6	0.0E+00	1.8E-03	2.2E-02	3.4E-03	2.1E-04	0.0E+00	0.0E+00	2.4E-03	1.2E-03	0.0E+00	0.0E+00	0.0E+00	1.0E-04	0.0E+00	0.0E+00	0.0E+00	0.0E+00	3.1E-02
KEOST2014-2015	3	2014/8/6	2014/8/24	0.0E+00	0.0E+00	1.4E-03	0.0E+00	0.0E+00	0.0E+00	0.0E+00	3.1E-03	2.9E-03	0.0E+00	0.0E+00	0.0E+00	1.9E-03	0.0E+00	0.0E+00	0.0E+00	0.0E+00	9.3E-03
KEOST2014-2015	4	2014/8/24	2014/9/11	0.0E+00	0.0E+00	3.5E-02	0.0E+00	1.2E-03	0.0E+00	0.0E+00	1.4E-02	6.2E-15	0.0E+00	0.0E+00	0.0E+00	6.8E-04	1.7E-04	0.0E+00	0.0E+00	0.0E+00	5.1E-02
KEOST2014-2015	5	2014/9/11	2014/9/29	0.0E+00	2.5E-04	0.0E+00	1.8E-03	9.5E-05	0.0E+00	0.0E+00	1.1E-02	0.0E+00	0.0E+00	0.0E+00	0.0E+00	3.0E-04	0.0E+00	0.0E+00	0.0E+00	0.0E+00	1.3E-02
KEOST2014-2015	6	2014/9/29	2014/10/17	0.0E+00	0.0E+00	3.3E-04	2.1E-03	2.4E-02	0.0E+00	0.0E+00	1.9E-03	1.4E-15	0.0E+00	0.0E+00	0.0E+00	6.6E-15	0.0E+00	0.0E+00	0.0E+00	2.2E-03	3.0E-02
KEOST2014-2015	7	2014/10/17	2014/11/4	0.0E+00	0.0E+00	5.1E-04	3.8E-03	0.0E+00	0.0E+00	0.0E+00	1.8E-02	0.0E+00	0.0E+00	0.0E+00	0.0E+00	4.6E-04	0.0E+00	0.0E+00	0.0E+00	0.0E+00	2.3E-02
KEOST2014-2015	8	2014/11/4	2014/11/22	0.0E+00	0.0E+00	0.0E+00	3.4E-03	4.5E-04	0.0E+00	0.0E+00	1.9E-03	0.0E+00	0.0E+00	0.0E+00	0.0E+00	1.0E-04	0.0E+00	0.0E+00	1.1E-03	0.0E+00	7.0E-03
KEOST2014-2015	9	2014/11/22	2014/12/10	0.0E+00	0.0E+00	1.0E-04	0.0E+00	0.0E+00	0.0E+00	0.0E+00	8.2E-03	0.0E+00	0.0E+00	0.0E+00	0.0E+00	3.0E-04	0.0E+00	0.0E+00	0.0E+00	3.8E-04	9.0E-03
KEOST2014-2015	10	2014/12/10	2014/12/28	0.0E+00	0.0E+00	6.1E-04	3.2E-03	4.5E-04	0.0E+00	1.9E-03	2.5E-03	2.1E-03	0.0E+00	0.0E+00	0.0E+00	6.8E-04	0.0E+00	0.0E+00	1.4E-04	0.0E+00	1.2E-02
KEOST2014-2015	11	2014/12/28	2015/1/15	0.0E+00	0.0E+00	5.1E-04	1.5E-03	6.3E-04	0.0E+00	0.0E+00	4.8E-04	0.0E+00	9.2E-03	5.0E-04	0.0E+00	0.0E+00	0.0E+00	0.0E+00	0.0E+00	5.2E-03	1.8E-02
KEOST2014-2015	12	2015/1/15	2015/2/2	2.8E-04	6.2E-02	1.7E-01	2.0E-03	0.0E+00	0.0E+00	0.0E+00	3.5E-03	5.0E-15	0.0E+00	0.0E+00	0.0E+00	0.0E+00	0.0E+00	0.0E+00	1.9E-04	0.0E+00	2.4E-01
KEOST2014-2015	13	2015/2/2	2015/2/20	0.0E+00	0.0E+00	1.6E-01	0.0E+00	3.6E-04	0.0E+00	0.0E+00	4.4E-03	0.0E+00	0.0E+00	0.0E+00	0.0E+00	1.9E-03	0.0E+00	0.0E+00	0.0E+00	1.0E-03	1.7E-01
KEOST2014-2015	14	2015/2/20	2015/3/10	0.0E+00	7.9E-16	1.6E-03	1.0E-03	4.2E-04	0.0E+00	0.0E+00	3.1E-03	2.1E-03	0.0E+00	0.0E+00	2.2E-04	4.5E-03	0.0E+00	0.0E+00	9.2E-04	0.0E+00	1.4E-02
KEOST2014-2015	15	2015/3/10	2015/3/28	0.0E+00	2.8E-03	1.4E-03	1.0E-03	0.0E+00	0.0E+00	0.0E+00	6.3E-04	1.5E-04	0.0E+00	0.0E+00	0.0E+00	5.4E-04	0.0E+00	0.0E+00	0.0E+00	0.0E+00	6.6E-03
KEOST2014-2015	16	2015/3/28	2015/4/15	0.0E+00	3.3E-15	0.0E+00	0.0E+00	1.2E-01	0.0E+00	0.0E+00	4.0E-04	0.0E+00	0.0E+00	0.0E+00	2.0E-04	5.3E-04	0.0E+00	0.0E+00	5.0E-04	0.0E+00	1.2E-01
KEOST2014-2015	17	2015/4/15	2015/5/3	0.0E+00	0.0E+00	9.2E-04	4.2E-03	0.0E+00	0.0E+00	0.0E+00	3.1E-03	6.9E-14	0.0E+00	0.0E+00	0.0E+00	0.0E+00	0.0E+00	0.0E+00	0.0E+00	6.1E-02	6.9E-02
KEOST2014-2015	18	2015/5/3	2015/5/21	0.0E+00	0.0E+00	0.0E+00	6.9E-03	2.5E-03	0.0E+00	0.0E+00	4.2E-03	0.0E+00	0.0E+00	0.0E+00	0.0E+00	0.0E+00	0.0E+00	0.0E+00	0.0E+00	0.0E+00	1.4E-02
KEOST2014-2015	19	2015/5/21	2015/6/8	0.0E+00	4.4E-04	4.2E-02	3.2E-03	8.6E-04	0.0E+00	0.0E+00	3.9E-03	0.0E+00	0.0E+00	8.4E-04	0.0E+00	0.0E+00	0.0E+00	0.0E+00	0.0E+00	0.0E+00	5.1E-02
KEOST2014-2015	20	2015/6/8	2015/6/26	0.0E+00	7.9E-03	3.2E-03	0.0E+00	0.0E+00	0.0E+00	0.0E+00	3.2E-03	0.0E+00	0.0E+00	0.0E+00	0.0E+00	1.2E-03	3.4E-03	0.0E+00	2.1E-03	0.0E+00	2.1E-02
KEOST2014-2015	21	2015/6/26	2015/7/14	1.7E-03	6.6E-04	0.0E+00	3.0E-03	5.3E-03	0.0E+00	8.5E-03	3.8E-03	0.0E+00	0.0E+00	7.0E-04	0.0E+00	0.0E+00	0.0E+00	0.0E+00	0.0E+00	1.5E-03	2.5E-02
KEOST2015-2016	1	2015/9/10	2015/9/28	8.3E-04	0.0E+00	2.4E-03	0.0E+00	0.0E+00	2.6E-04	0.0E+00	1.1E-02	6.6E-04	0.0E+00	0.0E+00	0.0E+00	9.2E-16	0.0E+00	3.2E-16	0.0E+00	3.8E-04	1.6E-02
KEOST2015-2016	2	2015/9/28	2015/10/16	0.0E+00	7.3E-04	2.9E-02	6.7E-04	0.0E+00	0.0E+00	0.0E+00	2.5E-03	3.3E-04	0.0E+00	0.0E+00	0.0E+00	0.0E+00	0.0E+00	0.0E+00	0.0E+00	1.5E-02	4.7E-02
KEOST2015-2016	3	2015/10/16	2015/11/3	0.0E+00	0.0E+00	0.0E+00	6.8E-04	7.6E-04	7.7E-04	0.0E+00	0.0E+00	2.4E-02	0.0E+00	0.0E+00	0.0E+00	0.0E+00	0.0E+00	0.0E+00	0.0E+00	0.0E+00	2.7E-02
KEOST2015-2016	4	2015/11/3	2015/11/21	0.0E+00	0.0E+00	0.0E+00	0.0E+00	1.8E-03	0.0E+00	0.0E+00	2.5E-02	0.0E+00	0.0E+00	0.0E+00	0.0E+00	2.8E-03	0.0E+00	0.0E+00	0.0E+00	0.0E+00	3.0E-02
KEOST2015-2016	5	2015/11/21	2015/12/9	0.0E+00	0.0E+00	1.3E-03	1.9E-03	0.0E+00	0.0E+00	0.0E+00	8.4E-03	0.0E+00	0.0E+00	0.0E+00	0.0E+00	1.5E-03	0.0E+00	0.0E+00	0.0E+00	0.0E+00	1.3E-02
KEOST2015-2016	6	2015/12/9	2015/12/27	0.0E+00	1.2E-02	0.0E+00	0.0E+00	4.3E-04	0.0E+00	0.0E+00	8.0E-03	0.0E+00	0.0E+00	0.0E+00	0.0E+00	0.0E+00	0.0E+00	0.0E+00	0.0E+00	1.0E-02	3.0E-02
KEOST2015-2016	7	2015/12/27	2016/1/14	0.0E+00	8.7E-03	0.0E+00	3.0E-03	1.7E-03	0.0E+00	0.0E+00	3.7E-03	1.3E-02	0.0E+00	0.0E+00	0.0E+00	8.4E-04	0.0E+00	0.0E+00	0.0E+00	1.1E-15	3.1E-02
KEOST2015-2016	8	2016/1/14	2016/2/1	0.0E+00	0.0E+00	8.2E-04	0.0E+00	1.2E-03	0.0E+00	0.0E+00	8.5E-04	3.4E-15	0.0E+00	0.0E+00	0.0E+00	3.0E-04	0.0E+00	0.0E+00	0.0E+00	0.0E+00	3.2E-03
KEOST2015-2016	9	2016/2/1	2016/2/19	0.0E+00	0.0E+00	0.0E+00	1.2E-03	7.6E-04	1.2E-03	0.0E+00	3.7E-03	2.9E-15	0.0E+00	0.0E+00	0.0E+00	6.8E-04	0.0E+00	0.0E+00	0.0E+00	0.0E+00	7.5E-03
KEOST2015-2016	10	2016/2/19	2016/3/8	0.0E+00	0.0E+00	0.0E+00	4.5E-04	6.3E-04	0.0E+00	0.0E+00	5.0E-03	5.8E-04	0.0E+00	0.0E+00	0.0E+00	0.0E+00	0.0E+00	0.0E+00	1.3E-15	0.0E+00	6.7E-03
KEOST2015-2016	11	2016/3/8	2016/3/26	0.0E+00	0.0E+00	0.0E+00	0.0E+00	2.4E-03	0.0E+00	0.0E+00	2.8E-03	2.2E-02	0.0E+00	0.0E+00	0.0E+00	1.5E-03	0.0E+00	0.0E+00	0.0E+00	0.0E+00	2.9E-02
KEOST2015-2016	12	2016/3/26	2016/4/13	5.2E-03	0.0E+00	4.5E-03	0.0E+00	0.0E+00	0.0E+00	0.0E+00	1.0E-02	5.5E-03	0.0E+00	0.0E+00	0.0E+00	0.0E+00	0.0E+00	0.0E+00	0.0E+00	0.0E+00	2.6E-02
KEOST2015-2016	13	2016/4/13	2016/5/1	0.0E+00	0.0E+00	6.9E-03	0.0E+00	6.8E-03	0.0E+00	0.0E+00	1.4E-03	5.6E-03	0.0E+00	0.0E+00	0.0E+00	7.6E-04	0.0E+00	0.0E+00	0.0E+00	0.0E+00	2.2E-02
KEOST2015-2016	14	2016/5/1	2016/5/19	3.7E-04	0.0E+00	8.3E-03	0.0E+00	0.0E+00	0.0E+00	0.0E+00	7.1E-04	2.7E-15	0.0E+00	0.0E+00	0.0E+00	2.3E-04	0.0E+00	0.0E+00	0.0E+00	0.0E+00	9.6E-03
KEOST2015-2016	15	2016/5/19	2016/6/6	0.0E+00	0.0E+00	0.0E+00	0.0E+00	8.6E-04	0.0E+00	0.0E+00	4.4E-03	0.0E+00	0.0E+00	0.0E+00	0.0E+00	0.0E+00	0.0E+00	0.0E+00	0.0E+00	7.6E-04	6.0E-03
KEOST2015-2016	16	2016/6/6	2016/6/24	0.0E+00	0.0E+00	0.0E+00	7.5E-04	2.5E-03	2.8E-03	0.0E+00	0.0E+00	1.3E-14	0.0E+00	0.0E+00	0.0E+00	6.1E-04	0.0E+00	2.3E-03	0.0E+00	0.0E+00	9.0E-03
KEOST2016-2017	1	2016/7/10	2016/7/31	0.0E+00	0.0E+00	1.8E-03	0.0E+00	0.0E+00	6.6E-04	0.0E+00	1.7E-02	1.7E-03	0.0E+00	1.3E-15	0.0E+00	0.0E+00	0.0E+00	0.0E+00	0.0E+00	0.0E+00	2.1E-02
KEOST2016-2017	2	2016/7/31	2016/8/21	0.0E+00	1.2E-16	0.0E+00	3.6E-03	0.0E+00	0.0E+00	0.0E+00	1.2E-01	0.0E+00	0.0E+00	0.0E+00	0.0E+00	3.9E-04	0.0E+00	0.0E+00	0.0E+00	2.3E-03	1.3E-01
KEOST2016-2017	3	2016/8/21	2016/9/11	0.0E+00	2.5E-16	0.0E+00	2.1E-03	0.0E+00	0.0E+00	0.0E+00	8.4E-03	2.4E-03	0.0E+00	0.0E+00	0.0E+00	0.0E+00	0.0E+00	0.0E+00	0.0E+00	6.6E-04	1.4E-02
KEOST2016-2017	4	2016/9/11	2016/10/2	0.0E+00	0.0E+00	0.0E+00	3.0E-03	0.0E+00	0.0E+00	0.0E+00	2.7E-03	0.0E+00	0.0E+00	0.0E+00	0.0E+00	3.4E-03	0.0E+00	0.0E+00	0.0E+00	0.0E+00	9.1E-03

Table S14. Summary and calculated volume and mass of individual microplastic particles in sediment in the western North Pacific as reported by Tsuchiya[7]. Polymer-type abbreviations are as shown in Table S1

Station	Core	Sediment layer (cm)	Oceanographic setting	Area	Particle number in Tsuchiya et al. (2024)	Major axis (μm)	Minor axis (μm)	Polymer type	Shape	Aspect ratio	Volume (cm^3)	Mass (g)
St. 1	a	0–1	Bathyal (slope)	Sagami Bay	1	234.9	110.5	EVA	grain	0.47	7.7E-07	7.2E-07
St. 1	a	0–1	Bathyal (slope)	Sagami Bay	2	128.0	65.9	PE	grain	0.51	1.5E-07	1.4E-07
St. 1	a	0–1	Bathyal (slope)	Sagami Bay	3	106.1	55.3	PE	grain	0.52	8.7E-08	8.3E-08
St. 1	a	0–1	Bathyal (slope)	Sagami Bay	6	71.4	41.4	PP	grain	0.58	3.3E-08	3.0E-08
St. 1	a	0–1	Bathyal (slope)	Sagami Bay	7	38.8	14.5	PP	grain	0.37	2.2E-09	2.0E-09
St. 1	b	0–1	Bathyal (slope)	Sagami Bay	2	126.4	75.2	PP	grain	0.59	1.9E-07	1.7E-07
St. 1	b	0–1	Bathyal (slope)	Sagami Bay	3	42.9	28.0	PP	grain	0.65	9.1E-09	8.2E-09
St. 1	b	0–1	Bathyal (slope)	Sagami Bay	4	183.0	58.8	PP	grain	0.32	1.7E-07	1.5E-07
St. 1	b	0–1	Bathyal (slope)	Sagami Bay	5	93.6	28.5	PS	grain	0.30	2.0E-08	2.1E-08
St. 1	b	0–1	Bathyal (slope)	Sagami Bay	6	66.7	45.0	PE	grain	0.67	3.6E-08	3.4E-08
St. 1	b	0–1	Bathyal (slope)	Sagami Bay	7	89.8	51.8	PE	grain	0.58	6.5E-08	6.1E-08
St. 1	b	0–1	Bathyal (slope)	Sagami Bay	8	59.1	19.4	PE	grain	0.33	6.0E-09	5.7E-09
St. 1	b	0–1	Bathyal (slope)	Sagami Bay	9	79.7	49.5	PE	grain	0.62	5.3E-08	5.0E-08
St. 1	b	0–1	Bathyal (slope)	Sagami Bay	10	174.9	74.4	PE	grain	0.43	2.6E-07	2.5E-07
St. 1	b	0–1	Bathyal (slope)	Sagami Bay	12	144.2	20.7	PET	grain	0.14	1.7E-08	2.2E-08
St. 1	b	0–1	Bathyal (slope)	Sagami Bay	13	46.1	31.8	PP	grain	0.69	1.3E-08	1.1E-08
St. 1	c	0–1	Bathyal (slope)	Sagami Bay	1	67.9	33.6	PE	grain	0.49	2.1E-08	1.9E-08
St. 1	c	0–1	Bathyal (slope)	Sagami Bay	2	152.6	40.4	PET	grain	0.26	6.7E-08	9.1E-08
St. 1	c	0–1	Bathyal (slope)	Sagami Bay	4	109.7	39.4	PP	grain	0.36	4.6E-08	4.2E-08
St. 1	c	0–1	Bathyal (slope)	Sagami Bay	5	43.4	19.3	PP	grain	0.44	4.4E-09	3.9E-09
St. 1	c	0–1	Bathyal (slope)	Sagami Bay	6	75.6	42.9	PP	grain	0.57	3.8E-08	3.4E-08
St. 1	c	0–1	Bathyal (slope)	Sagami Bay	7	159.5	123.7	PP	grain	0.78	6.6E-07	6.0E-07
St. 1	c	0–1	Bathyal (slope)	Sagami Bay	8	31.3	20.2	PP	grain	0.65	3.4E-09	3.1E-09

St. 2	a	0–1	Bathyal (central basin)	Sagami Bay	1	47.5	33.6	EVOH	grain	0.71	1.7E-08	1.9E-08
St. 2	a	0–1	Bathyal (central basin)	Sagami Bay	2	105.2	31.4	PET	grain	0.30	3.2E-08	4.4E-08
St. 2	a	0–1	Bathyal (central basin)	Sagami Bay	3	51.2	27.5	PET	grain	0.54	1.2E-08	1.6E-08
St. 2	a	0–1	Bathyal (central basin)	Sagami Bay	4	33.2	32.7	PMP	beads	0.98	1.1E-08	9.3E-09
St. 2	a	0–1	Bathyal (central basin)	Sagami Bay	5	44.3	20.8	PP	grain	0.47	6.0E-09	5.4E-09
St. 2	a	0–1	Bathyal (central basin)	Sagami Bay	6	50.0	16.5	PP	grain	0.33	4.2E-09	3.8E-09
St. 2	a	0–1	Bathyal (central basin)	Sagami Bay	7	56.9	28.5	PP	grain	0.50	1.4E-08	1.3E-08
St. 2	a	0–1	Bathyal (central basin)	Sagami Bay	8	158.7	42.3	PP	grain	0.27	8.8E-08	8.0E-08
St. 2	a	0–1	Bathyal (central basin)	Sagami Bay	9	54.9	26.3	PS	grain	0.48	1.2E-08	1.2E-08
St. 2	a	0–1	Bathyal (central basin)	Sagami Bay	10	334.6	246.6	VC/VA	grain	0.74	6.3E-06	8.6E-06
St. 2	a	0–1	Bathyal (central basin)	Sagami Bay	11	194.0	151.1	PE	grain	0.78	1.4E-06	1.3E-06
St. 2	a	0–1	Bathyal (central basin)	Sagami Bay	12	60.4	36.4	PE	grain	0.60	2.5E-08	2.4E-08
St. 2	a	0–1	Bathyal (central basin)	Sagami Bay	13	86.5	39.3	PE	grain	0.45	4.2E-08	3.9E-08
St. 2	a	0–1	Bathyal (central basin)	Sagami Bay	14	97.9	48.2	PE	grain	0.49	7.1E-08	6.7E-08
St. 2	a	0–1	Bathyal (central basin)	Sagami Bay	15	61.3	13.2	PE	grain	0.22	3.3E-09	3.1E-09
St. 2	a	0–1	Bathyal (central basin)	Sagami Bay	16	51.9	18.2	PET	grain	0.35	5.4E-09	7.2E-09
St. 2	b	0–1	Bathyal (central basin)	Sagami Bay	1	34.4	23.6	EVA	grain	0.69	6.0E-09	5.5E-09
St. 2	b	0–1	Bathyal (central basin)	Sagami Bay	2	39.9	28.1	PE	grain	0.70	9.8E-09	9.3E-09
St. 2	b	0–1	Bathyal (central basin)	Sagami Bay	3	90.7	38.7	PE	grain	0.43	4.2E-08	4.0E-08
St. 2	b	0–1	Bathyal (central basin)	Sagami Bay	4	47.5	40.0	PET	grain	0.84	2.4E-08	3.2E-08
St. 2	b	0–1	Bathyal (central basin)	Sagami Bay	5	63.6	41.2	PMMA	grain	0.65	3.4E-08	4.0E-08
St. 2	b	0–1	Bathyal (central basin)	Sagami Bay	6	301.5	117.9	PMMA	grain	0.39	1.3E-06	1.5E-06
St. 2	b	0–1	Bathyal (central basin)	Sagami Bay	7	46.6	22.9	PMMA	grain	0.49	7.6E-09	9.0E-09
St. 2	b	0–1	Bathyal (central basin)	Sagami Bay	8	98.5	74.6	PP	grain	0.76	1.7E-07	1.5E-07
St. 2	b	0–1	Bathyal (central basin)	Sagami Bay	9	63.6	44.4	PP	grain	0.70	3.9E-08	3.5E-08
St. 2	b	0–1	Bathyal (central basin)	Sagami Bay	10	43.8	19.3	PP	grain	0.44	5.1E-09	4.6E-09
St. 2	b	0–1	Bathyal (central basin)	Sagami Bay	11	49.5	32.1	PP	grain	0.65	1.6E-08	1.4E-08
St. 2	b	0–1	Bathyal (central basin)	Sagami Bay	12	52.1	39.3	EVOH	grain	0.75	2.5E-08	2.9E-08
St. 2	b	0–1	Bathyal (central basin)	Sagami Bay	13	29.5	28.0	PP	grain	0.95	7.2E-09	6.5E-09

St. 2	b	0–1	Bathyal (central basin)	Sagami Bay	14	53.6	34.7	PP	grain	0.65	2.0E-08	1.8E-08
St. 2	b	0–1	Bathyal (central basin)	Sagami Bay	15	54.8	36.8	PP	grain	0.67	2.3E-08	2.1E-08
St. 2	b	0–1	Bathyal (central basin)	Sagami Bay	16	83.2	74.2	PP	grain	0.89	1.4E-07	1.3E-07
St. 2	b	0–1	Bathyal (central basin)	Sagami Bay	17	36.8	30.6	PS	grain	0.83	1.1E-08	1.1E-08
St. 2	b	0–1	Bathyal (central basin)	Sagami Bay	18	75.3	32.2	PS	grain	0.43	2.4E-08	2.5E-08
St. 2	b	0–1	Bathyal (central basin)	Sagami Bay	19	57.0	38.0	EVOH	grain	0.67	2.6E-08	2.9E-08
St. 2	b	0–1	Bathyal (central basin)	Sagami Bay	20	50.8	38.0	EVOH	grain	0.75	2.3E-08	2.6E-08
St. 2	b	0–1	Bathyal (central basin)	Sagami Bay	21	72.9	32.3	PE	grain	0.44	2.4E-08	2.2E-08
St. 2	b	0–1	Bathyal (central basin)	Sagami Bay	22	79.5	41.2	PE	grain	0.52	4.2E-08	4.0E-08
St. 2	b	0–1	Bathyal (central basin)	Sagami Bay	23	19.9	14.3	PE	grain	0.72	1.3E-09	1.2E-09
St. 2	b	0–1	Bathyal (central basin)	Sagami Bay	24	24.6	13.6	PE	grain	0.55	1.4E-09	1.3E-09
St. 2	b	0–1	Bathyal (central basin)	Sagami Bay	25	52.2	32.6	PE	grain	0.62	1.7E-08	1.6E-08
St. 2	c	0–1	Bathyal (basin central)	Sagami Bay	1	35.4	33.4	EVOH	grain	0.94	1.2E-08	1.4E-08
St. 2	c	0–1	Bathyal (basin central)	Sagami Bay	2	44.9	20.3	PE	grain	0.45	5.8E-09	5.4E-09
St. 2	c	0–1	Bathyal (basin central)	Sagami Bay	3	99.5	26.4	PET	grain	0.27	2.2E-08	2.9E-08
St. 2	c	0–1	Bathyal (basin central)	Sagami Bay	4	33.6	21.9	PET	grain	0.65	5.0E-09	6.8E-09
St. 2	c	0–1	Bathyal (basin central)	Sagami Bay	5	40.8	29.0	PET	grain	0.71	1.1E-08	1.4E-08
St. 2	c	0–1	Bathyal (basin central)	Sagami Bay	6	30.9	12.4	PET	grain	0.40	1.5E-09	2.0E-09
St. 2	c	0–1	Bathyal (basin central)	Sagami Bay	7	33.5	27.0	PET	grain	0.81	7.6E-09	1.0E-08
St. 2	c	0–1	Bathyal (basin central)	Sagami Bay	8	151.4	149.0	PMMA	grain	0.98	1.0E-06	1.2E-06
St. 2	c	0–1	Bathyal (basin central)	Sagami Bay	9	30.2	24.2	PP	grain	0.80	5.5E-09	5.0E-09
St. 2	c	0–1	Bathyal (basin central)	Sagami Bay	10	38.6	16.1	PP	grain	0.42	3.1E-09	2.8E-09
St. 2	c	0–1	Bathyal (basin central)	Sagami Bay	11	77.2	31.4	PP	grain	0.41	2.4E-08	2.1E-08
St. 2	c	0–1	Bathyal (basin central)	Sagami Bay	12	42.7	32.0	EVOH	grain	0.75	1.4E-08	1.6E-08
St. 2	c	0–1	Bathyal (basin central)	Sagami Bay	13	38.1	25.4	PP	grain	0.67	7.7E-09	6.9E-09
St. 2	c	0–1	Bathyal (basin central)	Sagami Bay	14	50.0	26.8	PP	grain	0.54	1.1E-08	1.0E-08
St. 2	c	0–1	Bathyal (basin central)	Sagami Bay	15	82.1	41.9	PP	grain	0.51	4.5E-08	4.1E-08
St. 2	c	0–1	Bathyal (basin central)	Sagami Bay	16	29.5	19.0	PP	grain	0.64	3.3E-09	3.0E-09
St. 2	c	0–1	Bathyal (basin central)	Sagami Bay	17	22.5	21.5	PP	grain	0.96	3.2E-09	2.9E-09

St. 2	c	0–1	Bathyal (basin central)	Sagami Bay	18	49.1	27.6	PP	grain	0.56	1.2E-08	1.1E-08
St. 2	c	0–1	Bathyal (basin central)	Sagami Bay	19	32.0	15.9	PP	grain	0.50	2.5E-09	2.3E-09
St. 2	c	0–1	Bathyal (basin central)	Sagami Bay	20	46.7	27.4	PP	grain	0.59	1.1E-08	9.9E-09
St. 2	c	0–1	Bathyal (basin central)	Sagami Bay	21	170.6	149.1	PP	grain	0.87	1.2E-06	1.1E-06
St. 2	c	0–1	Bathyal (basin central)	Sagami Bay	22	55.3	36.7	PP	grain	0.66	2.3E-08	2.1E-08
St. 2	c	0–1	Bathyal (basin central)	Sagami Bay	23	52.2	38.1	EVOH	grain	0.73	2.4E-08	2.7E-08
St. 2	c	0–1	Bathyal (basin central)	Sagami Bay	24	63.2	30.3	PP	grain	0.48	1.8E-08	1.6E-08
St. 2	c	0–1	Bathyal (basin central)	Sagami Bay	25	46.9	37.1	PP	grain	0.79	2.0E-08	1.8E-08
St. 2	c	0–1	Bathyal (basin central)	Sagami Bay	26	29.7	17.3	PP	grain	0.58	2.8E-09	2.5E-09
St. 2	c	0–1	Bathyal (basin central)	Sagami Bay	27	96.3	31.0	PP	grain	0.32	2.9E-08	2.6E-08
St. 2	c	0–1	Bathyal (basin central)	Sagami Bay	28	90.3	30.1	PP	grain	0.33	2.5E-08	2.3E-08
St. 2	c	0–1	Bathyal (basin central)	Sagami Bay	29	100.8	26.4	PS	grain	0.26	2.2E-08	2.3E-08
St. 2	c	0–1	Bathyal (basin central)	Sagami Bay	30	143.9	41.4	PS	grain	0.29	7.7E-08	8.0E-08
St. 2	c	0–1	Bathyal (basin central)	Sagami Bay	31	90.6	47.7	PS	grain	0.53	6.4E-08	6.7E-08
St. 2	c	0–1	Bathyal (basin central)	Sagami Bay	32	48.4	34.2	EVOH	grain	0.71	1.8E-08	2.0E-08
St. 2	c	0–1	Bathyal (basin central)	Sagami Bay	33	47.9	34.4	PE	grain	0.72	1.8E-08	1.7E-08
St. 2	c	0–1	Bathyal (basin central)	Sagami Bay	34	138.6	49.9	PE	grain	0.36	1.1E-07	1.0E-07
St. 2	c	0–1	Bathyal (basin central)	Sagami Bay	35	47.4	23.7	PE	grain	0.50	8.3E-09	7.8E-09
St. 2	c	0–1	Bathyal (basin central)	Sagami Bay	36	20.1	17.7	PE	grain	0.88	2.0E-09	1.8E-09
St. 2	c	0–1	Bathyal (basin central)	Sagami Bay	37	120.4	35.4	PE	grain	0.29	4.7E-08	4.4E-08
St. 4	a	0–1	Hadal	Trench (triple junction)	1	99.6	71.8	EVOH	grain	0.72	1.4E-07	1.6E-07
St. 4	a	0–1	Hadal	Trench (triple junction)	2	86.8	39.2	PET	grain	0.45	3.6E-08	4.9E-08
St. 4	a	0–1	Hadal	Trench (triple junction)	3	113.3	21.9	PET	grain	0.19	1.5E-08	2.0E-08
St. 4	a	0–1	Hadal	Trench (triple junction)	4	54.9	28.3	PET	grain	0.52	1.2E-08	1.6E-08
St. 4	a	0–1	Hadal	Trench (triple junction)	5	41.3	16.7	PET	grain	0.40	3.1E-09	4.2E-09
St. 4	a	0–1	Hadal	Trench (triple junction)	6	137.3	73.5	PP	grain	0.54	2.0E-07	1.8E-07
St. 4	a	0–1	Hadal	Trench (triple junction)	7	223.6	127.8	PP	grain	0.57	9.9E-07	8.9E-07
St. 4	a	0–1	Hadal	Trench (triple junction)	8	59.3	27.1	PP	grain	0.46	1.2E-08	1.1E-08
St. 4	a	0–1	Hadal	Trench (triple junction)	9	43.1	34.7	PP	grain	0.81	1.4E-08	1.3E-08

St. 4	a	0–1	Hadal	Trench (triple junction)	10	37.5	25.0	EVOH	grain	0.67	6.3E-09	7.3E-09
St. 4	a	0–1	Hadal	Trench (triple junction)	11	40.3	35.1	EVOH	grain	0.87	1.3E-08	1.5E-08
St. 4	a	0–1	Hadal	Trench (triple junction)	12	41.1	36.8	EVOH	grain	0.90	1.5E-08	1.7E-08
St. 4	a	0–1	Hadal	Trench (triple junction)	13	34.9	22.0	PE	grain	0.63	4.6E-09	4.3E-09
St. 4	a	0–1	Hadal	Trench (triple junction)	14	50.8	32.7	PE	grain	0.64	1.5E-08	1.4E-08
St. 4	a	0–1	Hadal	Trench (triple junction)	15	60.1	31.1	PE	grain	0.52	1.6E-08	1.5E-08
St. 4	a	0–1	Hadal	Trench (triple junction)	16	160.2	71.3	PE	grain	0.45	2.2E-07	2.1E-07
St. 4	a	0–1	Hadal	Trench (triple junction)	17	34.4	28.9	PE	grain	0.84	7.8E-09	7.3E-09
St. 4	b	0–1	Hadal	Trench (triple junction)	1	96.7	36.4	EVA	grain	0.38	3.5E-08	3.2E-08
St. 4	b	0–1	Hadal	Trench (triple junction)	2	115.9	33.1	PP	grain	0.29	3.4E-08	3.1E-08
St. 4	b	0–1	Hadal	Trench (triple junction)	3	60.1	29.7	PP	grain	0.49	1.4E-08	1.3E-08
St. 4	b	0–1	Hadal	Trench (triple junction)	4	45.8	21.9	PP	grain	0.48	5.9E-09	5.4E-09
St. 4	b	0–1	Hadal	Trench (triple junction)	5	42.5	29.8	PP	grain	0.70	1.0E-08	9.2E-09
St. 4	b	0–1	Hadal	Trench (triple junction)	6	55.7	26.3	PVC	grain	0.47	1.0E-08	1.5E-08
St. 4	b	0–1	Hadal	Trench (triple junction)	7	43.6	20.5	EVA	grain	0.47	5.0E-09	4.6E-09
St. 4	b	0–1	Hadal	Trench (triple junction)	8	45.3	34.3	PC	grain	0.76	1.4E-08	1.9E-08
St. 4	b	0–1	Hadal	Trench (triple junction)	9	35.2	13.2	PE	grain	0.38	1.7E-09	1.6E-09
St. 4	b	0–1	Hadal	Trench (triple junction)	10	54.9	37.2	PE	grain	0.68	2.1E-08	1.9E-08
St. 4	b	0–1	Hadal	Trench (triple junction)	11	76.4	55.7	PE	grain	0.73	6.4E-08	6.0E-08
St. 4	b	0–1	Hadal	Trench (triple junction)	12	98.6	10.0	PET	grain	0.10	2.7E-09	3.6E-09
St. 4	b	0–1	Hadal	Trench (triple junction)	14	88.8	30.9	PMMA	grain	0.35	2.3E-08	2.7E-08
St. 5	a	0–1	Hadal	Trench (triple junction)	1	110.3	59.8	EVA	grain	0.54	1.0E-07	9.3E-08
St. 5	a	0–1	Hadal	Trench (triple junction)	2	301.3	143.1	PP	grain	0.47	1.6E-06	1.4E-06
St. 5	a	0–1	Hadal	Trench (triple junction)	3	53.8	18.6	PP	grain	0.35	4.7E-09	4.3E-09
St. 5	a	0–1	Hadal	Trench (triple junction)	4	66.4	29.0	PS	grain	0.44	1.4E-08	1.5E-08
St. 5	a	0–1	Hadal	Trench (triple junction)	5	34.1	24.0	EVA	grain	0.70	5.0E-09	4.6E-09
St. 5	a	0–1	Hadal	Trench (triple junction)	6	31.4	18.7	PE	grain	0.60	2.8E-09	2.6E-09
St. 5	a	0–1	Hadal	Trench (triple junction)	7	89.2	37.6	PE	grain	0.42	3.2E-08	3.0E-08
St. 5	a	0–1	Hadal	Trench (triple junction)	8	98.4	27.2	PET	grain	0.28	1.8E-08	2.5E-08

St. 5	a	0–1	Hadal	Trench (triple junction)	9	47.3	37.3	PP	grain	0.79	1.7E-08	1.5E-08
St. 5	a	0–1	Hadal	Trench (triple junction)	11	55.0	21.1	PP	grain	0.38	6.2E-09	5.6E-09
St. 5	a	0–1	Hadal	Trench (triple junction)	12	59.4	40.3	PP	grain	0.68	2.4E-08	2.2E-08
St. 5	b	0–1	Hadal	Trench (triple junction)	1	47.5	37.5	EVOH	grain	0.79	1.7E-08	1.9E-08
St. 5	b	0–1	Hadal	Trench (triple junction)	2	95.0	80.1	PE	grain	0.84	1.5E-07	1.5E-07
St. 5	b	0–1	Hadal	Trench (triple junction)	3	91.1	90.0	PE	grain	0.99	1.9E-07	1.8E-07
St. 5	b	0–1	Hadal	Trench (triple junction)	4	88.5	41.5	PE	grain	0.47	3.9E-08	3.6E-08
St. 5	b	0–1	Hadal	Trench (triple junction)	5	66.0	23.5	PE	grain	0.36	9.2E-09	8.7E-09
St. 5	b	0–1	Hadal	Trench (triple junction)	6	132.9	19.7	PE	grain	0.15	1.3E-08	1.2E-08
St. 5	b	0–1	Hadal	Trench (triple junction)	7	56.7	21.9	PE	grain	0.39	6.9E-09	6.5E-09
St. 5	b	0–1	Hadal	Trench (triple junction)	8	125.8	60.9	PE	grain	0.48	1.2E-07	1.1E-07
St. 5	b	0–1	Hadal	Trench (triple junction)	9	83.2	35.7	PE	grain	0.43	2.7E-08	2.5E-08
St. 5	b	0–1	Hadal	Trench (triple junction)	10	51.4	30.3	PE	grain	0.59	1.2E-08	1.1E-08
St. 5	b	0–1	Hadal	Trench (triple junction)	11	82.9	40.0	PE	grain	0.48	3.4E-08	3.2E-08
St. 5	b	0–1	Hadal	Trench (triple junction)	12	211.2	75.6	EVOH	grain	0.36	3.1E-07	3.5E-07
St. 5	b	0–1	Hadal	Trench (triple junction)	13	254.4	51.6	PET	grain	0.20	1.7E-07	2.3E-07
St. 5	b	0–1	Hadal	Trench (triple junction)	14	48.9	46.1	PET	grain	0.94	2.6E-08	3.6E-08
St. 5	b	0–1	Hadal	Trench (triple junction)	15	202.0	68.7	PP	grain	0.34	2.4E-07	2.2E-07
St. 5	b	0–1	Hadal	Trench (triple junction)	16	21.9	15.0	PP	grain	0.68	1.2E-09	1.1E-09
St. 5	b	0–1	Hadal	Trench (triple junction)	17	71.2	49.2	PP	grain	0.69	4.4E-08	4.0E-08
St. 5	b	0–1	Hadal	Trench (triple junction)	18	53.4	40.8	EVOH	grain	0.76	2.3E-08	2.6E-08
St. 5	b	0–1	Hadal	Trench (triple junction)	19	158.7	76.4	PE	grain	0.48	2.3E-07	2.2E-07
St. 5	b	0–1	Hadal	Trench (triple junction)	20	307.8	199.2	PE	grain	0.65	3.1E-06	2.9E-06
St. 5	b	0–1	Hadal	Trench (triple junction)	21	53.7	41.0	PE	grain	0.76	2.3E-08	2.2E-08
St. 5	b	0–1	Hadal	Trench (triple junction)	22	44.7	37.5	PE	grain	0.84	1.6E-08	1.5E-08
St. 5	b	0–1	Hadal	Trench (triple junction)	23	88.0	35.8	PE	grain	0.41	2.9E-08	2.7E-08
St. 5	b	0–1	Hadal	Trench (triple junction)	24	160.4	154.4	PE	grain	0.96	9.7E-07	9.1E-07
St. 8	a	0–1	Abyssal	Abyssal plain	1	47.3	39.8	PE	grain	0.84	2.8E-08	2.6E-08
St. 8	a	0–1	Abyssal	Abyssal plain	2	45.3	32.5	PP	grain	0.72	1.8E-08	1.6E-08

St. 8	a	0–1	Abyssal	Abyssal plain	3	65.6	33.9	PE	grain	0.52	2.8E-08	2.6E-08
St. 8	a	0–1	Abyssal	Abyssal plain	4	50.0	22.4	PE	grain	0.45	9.3E-09	8.7E-09
St. 8	a	0–1	Abyssal	Abyssal plain	5	48.1	25.5	PE	grain	0.53	1.2E-08	1.1E-08
St. 8	a	0–1	Abyssal	Abyssal plain	6	37.3	26.4	PE	grain	0.71	9.6E-09	9.1E-09
St. 8	a	0–1	Abyssal	Abyssal plain	7	45.1	32.0	PE	grain	0.71	1.7E-08	1.6E-08
St. 8	a	0–1	Abyssal	Abyssal plain	8	22.9	10.6	PE	grain	0.46	9.5E-10	9.0E-10
St. 8	a	0–1	Abyssal	Abyssal plain	9	36.3	33.3	PE	grain	0.92	1.5E-08	1.4E-08
St. 8	a	0–1	Abyssal	Abyssal plain	10	57.6	35.9	PE	grain	0.62	2.7E-08	2.6E-08
St. 8	a	0–1	Abyssal	Abyssal plain	11	1038.7	501.7	PS	grain	0.48	9.7E-05	1.0E-04
St. 8	b	0–1	Abyssal	Abyssal plain	1	42.6	35.0	PA	grain	0.82	1.9E-08	2.2E-08
St. 8	b	0–1	Abyssal	Abyssal plain	2	36.1	29.8	PE	grain	0.83	1.2E-08	1.1E-08
St. 8	b	0–1	Abyssal	Abyssal plain	3	64.8	26.8	PE	grain	0.41	1.7E-08	1.6E-08
St. 8	b	0–1	Abyssal	Abyssal plain	4	15.2	11.7	PE	grain	0.77	7.7E-10	7.2E-10
St. 8	b	0–1	Abyssal	Abyssal plain	5	24.9	16.2	PE	grain	0.65	2.4E-09	2.3E-09
St. 8	b	0–1	Abyssal	Abyssal plain	6	36.3	30.2	PE	grain	0.83	1.2E-08	1.2E-08
St. 8	b	0–1	Abyssal	Abyssal plain	7	49.5	21.2	PE	grain	0.43	8.2E-09	7.8E-09
St. 8	b	0–1	Abyssal	Abyssal plain	8	58.0	34.5	PE	grain	0.59	2.5E-08	2.4E-08
St. 8	c	0–1	Abyssal	Abyssal plain	1	34.5	15.0	PA	grain	0.43	2.9E-09	3.3E-09
St. 8	c	0–1	Abyssal	Abyssal plain	2	19.8	19.6	PE	grain	0.99	2.8E-09	2.6E-09
St. 8	c	0–1	Abyssal	Abyssal plain	3	27.3	27.1	PE	grain	0.99	7.4E-09	7.0E-09
St. 8	c	0–1	Abyssal	Abyssal plain	4	42.4	29.9	PMMA	grain	0.71	1.4E-08	1.7E-08
St. 9	a	0–1	Abyssal	Abyssal plain	1	50.9	46.6	PE	grain	0.92	3.9E-08	3.7E-08
St. 9	a	0–1	Abyssal	Abyssal plain	2	32.8	26.1	PE	grain	0.80	7.9E-09	7.4E-09
St. 9	a	0–1	Abyssal	Abyssal plain	3	26.8	22.2	PE	grain	0.83	4.6E-09	4.4E-09
St. 9	a	0–1	Abyssal	Abyssal plain	4	21.8	18.6	PE	grain	0.85	2.7E-09	2.5E-09
St. 9	a	0–1	Abyssal	Abyssal plain	5	106.1	61.9	PE	grain	0.58	1.4E-07	1.3E-07
St. 9	a	0–1	Abyssal	Abyssal plain	6	44.4	27.0	PE	grain	0.61	1.1E-08	1.1E-08
St. 9	a	0–1	Abyssal	Abyssal plain	7	41.3	17.1	PE	grain	0.41	4.2E-09	4.0E-09
St. 9	a	0–1	Abyssal	Abyssal plain	8	32.6	24.9	PE	grain	0.76	7.1E-09	6.7E-09

St. 9	a	0–1	Abyssal	Abyssal plain	9	37.9	31.3	PE	grain	0.83	1.3E-08	1.2E-08
St. 9	a	0–1	Abyssal	Abyssal plain	10	49.8	33.1	PE	grain	0.66	1.9E-08	1.8E-08
St. 9	a	0–1	Abyssal	Abyssal plain	11	64.8	40.9	PE	grain	0.63	3.8E-08	3.6E-08
St. 9	a	0–1	Abyssal	Abyssal plain	12	23.2	19.1	PE	grain	0.82	3.0E-09	2.8E-09
St. 9	a	0–1	Abyssal	Abyssal plain	13	32.7	20.7	PE	grain	0.63	4.9E-09	4.6E-09
St. 9	a	0–1	Abyssal	Abyssal plain	14	46.7	31.9	PE	grain	0.68	1.7E-08	1.6E-08
St. 9	a	0–1	Abyssal	Abyssal plain	15	28.3	12.2	PE	grain	0.43	1.5E-09	1.4E-09
St. 9	a	0–1	Abyssal	Abyssal plain	16	46.3	32.6	PE	grain	0.70	1.7E-08	1.6E-08
St. 9	a	0–1	Abyssal	Abyssal plain	17	113.7	62.3	PE	grain	0.55	1.6E-07	1.5E-07
St. 9	a	0–1	Abyssal	Abyssal plain	18	34.8	21.4	PE	grain	0.61	5.6E-09	5.3E-09
St. 9	a	0–1	Abyssal	Abyssal plain	19	48.9	33.6	PE	grain	0.69	1.9E-08	1.8E-08
St. 9	a	0–1	Abyssal	Abyssal plain	20	38.7	29.0	PE	grain	0.75	1.1E-08	1.1E-08
St. 9	b	0–1	Abyssal	Abyssal plain	1	42.3	23.3	PE	grain	0.55	8.1E-09	7.6E-09
St. 9	b	0–1	Abyssal	Abyssal plain	2	48.4	43.9	PE	grain	0.91	3.3E-08	3.1E-08
St. 9	b	0–1	Abyssal	Abyssal plain	3	57.1	37.9	PE	grain	0.66	2.9E-08	2.7E-08
St. 9	b	0–1	Abyssal	Abyssal plain	4	38.7	29.3	PE	grain	0.76	1.2E-08	1.1E-08
St. 9	b	0–1	Abyssal	Abyssal plain	5	41.5	26.0	PE	grain	0.63	9.9E-09	9.3E-09
St. 9	b	0–1	Abyssal	Abyssal plain	6	32.1	22.5	PE	grain	0.70	5.7E-09	5.4E-09
St. 9	b	0–1	Abyssal	Abyssal plain	7	93.5	41.4	PE	grain	0.44	5.6E-08	5.3E-08
St. 9	b	0–1	Abyssal	Abyssal plain	8	30.8	17.7	PE	grain	0.57	3.4E-09	3.2E-09
St. 9	b	0–1	Abyssal	Abyssal plain	9	30.5	21.5	PE	grain	0.70	5.0E-09	4.7E-09
St. 9	b	0–1	Abyssal	Abyssal plain	10	48.7	32.1	PE	grain	0.66	1.8E-08	1.7E-08
St. 9	b	0–1	Abyssal	Abyssal plain	11	45.3	20.3	PE	grain	0.45	6.6E-09	6.2E-09
St. 9	b	0–1	Abyssal	Abyssal plain	12	56.1	37.8	PE	grain	0.67	2.8E-08	2.7E-08
St. 9	b	0–1	Abyssal	Abyssal plain	13	38.7	25.9	PE	grain	0.67	9.1E-09	8.6E-09
St. 9	b	0–1	Abyssal	Abyssal plain	14	52.0	36.7	PE	grain	0.71	2.5E-08	2.3E-08
St. 9	b	0–1	Abyssal	Abyssal plain	15	83.7	48.3	PE	grain	0.58	6.9E-08	6.5E-08
St. 9	b	0–1	Abyssal	Abyssal plain	16	813.7	753.3	PE	grain	0.93	1.6E-04	1.5E-04
St. 9	c	0–1	Abyssal	Abyssal plain	1	57.6	43.7	PA	grain	0.76	3.9E-08	4.4E-08

St. 9	c	0–1	Abyssal	Abyssal plain	2	74.9	52.8	PE	grain	0.70	7.3E-08	6.9E-08
St. 9	c	0–1	Abyssal	Abyssal plain	3	63.5	38.9	PE	grain	0.61	3.4E-08	3.2E-08
St. 9	c	0–1	Abyssal	Abyssal plain	5	59.0	26.6	PET	grain	0.45	1.5E-08	2.0E-08
St. 10	a	0–1	Abyssal	Abyssal plain	1	98.1	34.5	PA	grain	0.35	4.2E-08	4.8E-08
St. 10	a	0–1	Abyssal	Abyssal plain	2	60.1	25.2	PE	grain	0.42	1.4E-08	1.3E-08
St. 10	a	0–1	Abyssal	Abyssal plain	3	52.2	44.6	PE	grain	0.85	3.8E-08	3.6E-08
St. 10	a	0–1	Abyssal	Abyssal plain	4	48.2	43.7	PE	grain	0.91	3.3E-08	3.2E-08
St. 10	a	0–1	Abyssal	Abyssal plain	5	46.2	37.7	PE	grain	0.82	2.4E-08	2.2E-08
St. 10	a	0–1	Abyssal	Abyssal plain	6	26.7	24.6	PE	grain	0.92	5.9E-09	5.5E-09
St. 10	a	0–1	Abyssal	Abyssal plain	7	33.4	26.8	PE	grain	0.80	8.7E-09	8.2E-09
St. 10	a	0–1	Abyssal	Abyssal plain	8	18.3	17.2	PE	grain	0.94	2.0E-09	1.9E-09
St. 10	a	0–1	Abyssal	Abyssal plain	9	31.6	19.8	PE	grain	0.63	4.5E-09	4.2E-09
St. 10	a	0–1	Abyssal	Abyssal plain	10	72.1	34.0	PE	grain	0.47	3.0E-08	2.9E-08
St. 10	a	0–1	Abyssal	Abyssal plain	11	50.4	26.4	PE	grain	0.52	1.3E-08	1.2E-08
St. 10	a	0–1	Abyssal	Abyssal plain	12	49.9	40.3	PA	grain	0.81	2.9E-08	3.4E-08
St. 10	a	0–1	Abyssal	Abyssal plain	13	51.0	33.3	PE	grain	0.65	2.1E-08	1.9E-08
St. 10	a	0–1	Abyssal	Abyssal plain	14	46.9	26.7	PP	grain	0.57	1.2E-08	1.1E-08
St. 10	a	0–1	Abyssal	Abyssal plain	15	24.9	20.8	PE	grain	0.84	3.9E-09	3.7E-09
St. 10	a	0–1	Abyssal	Abyssal plain	16	46.5	22.0	PE	grain	0.47	8.2E-09	7.7E-09
St. 10	a	0–1	Abyssal	Abyssal plain	17	29.6	28.8	PE	grain	0.97	8.9E-09	8.4E-09
St. 10	a	0–1	Abyssal	Abyssal plain	18	63.4	52.7	PE	grain	0.83	6.4E-08	6.0E-08
St. 10	a	0–1	Abyssal	Abyssal plain	19	52.4	41.2	PE	grain	0.79	3.2E-08	3.0E-08
St. 10	a	0–1	Abyssal	Abyssal plain	20	37.1	28.6	PE	grain	0.77	1.1E-08	1.0E-08
St. 10	a	0–1	Abyssal	Abyssal plain	21	29.7	25.6	PE	grain	0.86	7.1E-09	6.7E-09
St. 10	b	0–1	Abyssal	Abyssal plain	1	52.0	37.6	PA	grain	0.72	2.7E-08	3.0E-08
St. 10	b	0–1	Abyssal	Abyssal plain	2	38.7	28.9	PE	grain	0.75	1.2E-08	1.1E-08
St. 10	b	0–1	Abyssal	Abyssal plain	3	38.8	26.2	PE	grain	0.68	9.7E-09	9.1E-09
St. 10	b	0–1	Abyssal	Abyssal plain	4	44.1	16.8	PE	grain	0.38	4.5E-09	4.3E-09
St. 10	b	0–1	Abyssal	Abyssal plain	5	37.9	25.5	PE	grain	0.67	8.9E-09	8.4E-09

St. 10	b	0–1	Abyssal	Abyssal plain	6	57.2	39.8	PE	grain	0.70	3.3E-08	3.1E-08
St. 10	b	0–1	Abyssal	Abyssal plain	7	51.7	26.4	PE	grain	0.51	1.3E-08	1.2E-08
St. 10	b	0–1	Abyssal	Abyssal plain	8	140.4	69.4	PE	grain	0.49	2.5E-07	2.3E-07
St. 10	b	0–1	Abyssal	Abyssal plain	9	82.2	56.8	PE	grain	0.69	9.6E-08	9.1E-08
St. 10	b	0–1	Abyssal	Abyssal plain	10	54.8	28.9	PE	grain	0.53	1.7E-08	1.6E-08
St. 10	b	0–1	Abyssal	Abyssal plain	11	51.8	30.0	PE	grain	0.58	1.7E-08	1.6E-08
St. 10	b	0–1	Abyssal	Abyssal plain	12	60.5	32.9	PA	grain	0.54	2.4E-08	2.7E-08
St. 10	b	0–1	Abyssal	Abyssal plain	13	71.4	28.0	PE	grain	0.39	2.0E-08	1.9E-08
St. 10	b	0–1	Abyssal	Abyssal plain	14	36.4	29.2	PE	grain	0.80	1.1E-08	1.1E-08
St. 10	b	0–1	Abyssal	Abyssal plain	15	44.7	40.3	PE	grain	0.90	2.6E-08	2.5E-08
St. 10	b	0–1	Abyssal	Abyssal plain	16	71.1	39.2	PE	grain	0.55	4.0E-08	3.7E-08
St. 10	b	0–1	Abyssal	Abyssal plain	17	28.0	25.4	PE	grain	0.91	6.6E-09	6.2E-09
St. 10	b	0–1	Abyssal	Abyssal plain	18	71.2	38.7	PE	grain	0.54	3.9E-08	3.7E-08
St. 10	b	0–1	Abyssal	Abyssal plain	19	62.9	47.6	PE	grain	0.76	5.2E-08	4.9E-08
St. 10	b	0–1	Abyssal	Abyssal plain	20	83.9	38.8	PE	grain	0.46	4.6E-08	4.3E-08
St. 10	b	0–1	Abyssal	Abyssal plain	21	45.6	26.0	PE	grain	0.57	1.1E-08	1.1E-08
St. 10	b	0–1	Abyssal	Abyssal plain	22	62.4	22.7	PE	grain	0.36	1.2E-08	1.1E-08
St. 10	b	0–1	Abyssal	Abyssal plain	23	109.2	66.7	PA	grain	0.61	1.8E-07	2.0E-07
St. 10	b	0–1	Abyssal	Abyssal plain	24	45.0	36.9	PE	grain	0.82	2.2E-08	2.1E-08
St. 10	b	0–1	Abyssal	Abyssal plain	25	21.5	15.7	PE	grain	0.73	1.9E-09	1.8E-09
St. 10	b	0–1	Abyssal	Abyssal plain	26	15.5	13.8	PE	grain	0.89	1.1E-09	1.0E-09
St. 10	b	0–1	Abyssal	Abyssal plain	27	27.0	19.8	PE	grain	0.73	3.8E-09	3.6E-09
St. 10	b	0–1	Abyssal	Abyssal plain	28	49.1	36.1	PE	grain	0.74	2.3E-08	2.2E-08
St. 10	b	0–1	Abyssal	Abyssal plain	29	47.0	35.5	PE	grain	0.76	2.2E-08	2.0E-08
St. 10	b	0–1	Abyssal	Abyssal plain	30	35.2	20.0	PE	grain	0.57	5.1E-09	4.8E-09
St. 10	b	0–1	Abyssal	Abyssal plain	32	97.2	47.6	PE	grain	0.49	8.0E-08	7.5E-08
St. 10	b	0–1	Abyssal	Abyssal plain	33	63.5	45.4	PE	grain	0.71	4.8E-08	4.5E-08
St. 10	b	0–1	Abyssal	Abyssal plain	34	16.4	11.0	PE	grain	0.67	7.2E-10	6.8E-10
St. 10	b	0–1	Abyssal	Abyssal plain	35	30.4	28.2	PE	grain	0.93	8.8E-09	8.3E-09

St. 10	b	0–1	Abyssal	Abyssal plain	36	73.0	34.8	PE	grain	0.48	3.2E-08	3.0E-08
St. 10	b	0–1	Abyssal	Abyssal plain	37	36.3	24.5	PE	grain	0.67	7.9E-09	7.5E-09
St. 10	c	0–1	Abyssal	Abyssal plain	1	180.0	164.4	PA	grain	0.91	1.8E-06	2.0E-06
St. 10	c	0–1	Abyssal	Abyssal plain	2	112.3	99.6	PA	grain	0.89	4.0E-07	4.6E-07
St. 10	c	0–1	Abyssal	Abyssal plain	3	52.3	30.5	PE	grain	0.58	1.8E-08	1.7E-08

Table S15. Summary and calculated microplastic (MP) mass in sediment samples from the western North Pacific as reported by Tsuchiya[7]

Station	Latitude (N), longitude (E)	Area	Sediment layer (cm)	Years of accumulation	Particle size range (μm)	Aspect ratio (Mean ± S.D.)	Median aspect ratio	Shannon diversity (H') (Mean ± S.D.)	Buoyant polymer (%)	Dense polymer (%)	MP abundance range ($\times 10^4$ pieces m^{-}	Calculated MP mass range (mg m^{-2})
St. 1	35°00.9540', 139°13.3250'	Bathyal (Sagami Bay)	0–1	3–4	31–235 (101 ± 53)	0.49 ± 0.16	0.51	1.4 ± 0.2	89	11	1.3–2.4 (1.9 ± 0.5)	1.5–4.6 (2.5 ± 1.5)
St. 2	35°01.1484', 139°22.6592′	Bathyal (Sagami Bay)	0–1	3–4	20–335 (70 ± 54)	0.59 ± 0.19	0.59	2.3 ± 0.2	64	36	2.5–5.8 (4.1 ± 1.4)	3.6–16 (8.1 ± 5.6)
St. 4	34°20.4296', 142°00.6006'	Hadal (Triple junction)	0–1	15	34–224 (73 ± 43)	0.55 ± 0.19	0.52	2.3 ± 03	58	42	1.5–3.3 (2.4 ± 0.9)	0.28–3.2 (1.7 ± 1.4)
St. 5	33°46.4374', 141°56.1556'	Hadal (Triple junction)	0–1	15	22–308 (101 ± 73)	0.56 ± 0.22	0.48	1.7 ± 0.3	80	20	1.3–2.8 (2.1 ± 0.8)	1.9–6.5 (4.2 ± 2.3)
St. 8	33°00.2359', 145°00.7300'	Abyssal (Abyssal plain)	0–1	463–614	15–1039 (85 ± 204)	0.67 ± 0.18	0.71	0.8 ± 0.5	81	19	37.9–94.7 (69.5 ± 23.6)	2.8–34 (15 ± 13)
St. 9	34°45.2560', 144°59.7032'	Abyssal (Abyssal plain)	0–1	463–614	22–814 (68 ± 121)	0.67 ± 0.13	0.67	0.5 ± 0.7	95	5	37.9–189.5 (123.2 ± 63.3)	16–57 (40 ± 18)
St. 10	35°55.7558', 144°58.3896'	Abyssal (Abyssal plain)	0–1	463–614	16–180 (54 ± 29)	0.68 ± 0.17	0.69	0.7 ± 0.2	89	11	28.9–341.0 (189.6 ± 127.6)	38–236 (129 ± 82)

Table S16. Annual global polymer resin production as reported by Geyer et al.[8] and estimated microplastic mass flux at Stn. KEO

Year	Annual global polymer resin production (million tons)	Percentage of production in 2015	Estimated microplastic mass flux at St. KEO (mg m^{-2} year^{-1})
2015	381	100	20
2014	367	96.3	19
2013	352	92.4	18
2012	338	88.7	17
2011	325	85.3	17
2010	313	82.2	16
2009	288	75.6	15
2008	281	73.8	15
2007	295	77.4	15
2006	280	73.5	14
2005	263	69.0	14
2004	256	67.2	13
2003	241	63.3	12
2002	231	60.6	12
2001	218	57.2	11
2000	213	55.9	11
1999	202	53.0	10
1998	188	49.3	10
1997	180	47.2	9.3
1996	168	44.1	8.7
1995	156	40.9	8.1
1994	151	39.6	7.8
1993	137	36.0	7.1
1992	132	34.6	6.8
1991	124	32.5	6.4
1990	120	31.5	6.2
1989	114	29.9	5.9
1988	110	28.9	5.7
1987	104	27.3	5.4
1986	96	25.2	5.0
1985	90	23.6	4.7
1984	86	22.6	4.4
1983	80	21.0	4.1
1982	73	19.2	3.8
1981	72	18.9	3.7
1980	70	18.4	3.6
1979	71	18.6	3.7
1978	64	16.8	3.3
1977	59	15.5	3.1
1976	54	14.2	2.8

1975	46	12.1	2.4
1974	52	13.6	2.7
1973	51	13.4	2.6
1972	44	11.5	2.3
1971	38	10.0	2.0
1970	35	9.2	1.8
1969	32	8.4	1.7
1968	27	7.1	1.4
1967	23	6.0	1.2
1966	20	5.2	1.0
1965	17	4.5	0.88
1964	15	3.9	0.78
1963	13	3.4	0.67
1962	11	2.9	0.57
1961	9	2.4	0.47
1960	8	2.1	0.41
1959	7	1.8	0.36
1958	6	1.6	0.31
1957	5	1.3	0.26
1956	5	1.3	0.26
1955	4	1.0	0.21
1954	3	0.8	0.16
1953	3	0.8	0.16
1952	2	0.5	0.10
1951	2	0.5	0.10
1950	2	0.5	0.10
Total	7823		405

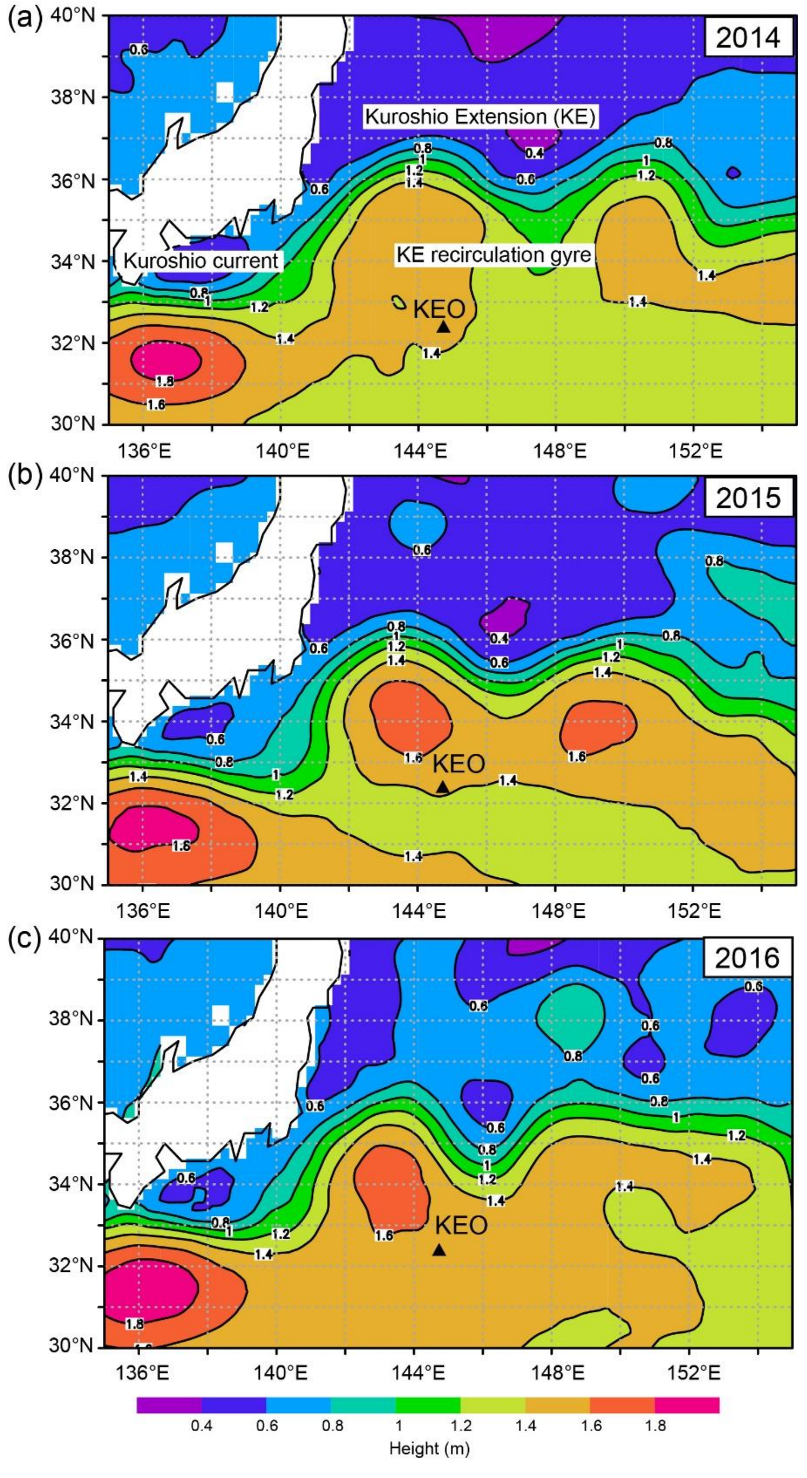

Figure S1. The Kuroshio Current, Kuroshio Extension (KE), and associated recirculation gyre at the study site. Contours and colors represent annual averaged absolute dynamic topography in (a) 2014, (b) 2015, and (c) 2016. The black triangle shows the location of Stn. KEO.

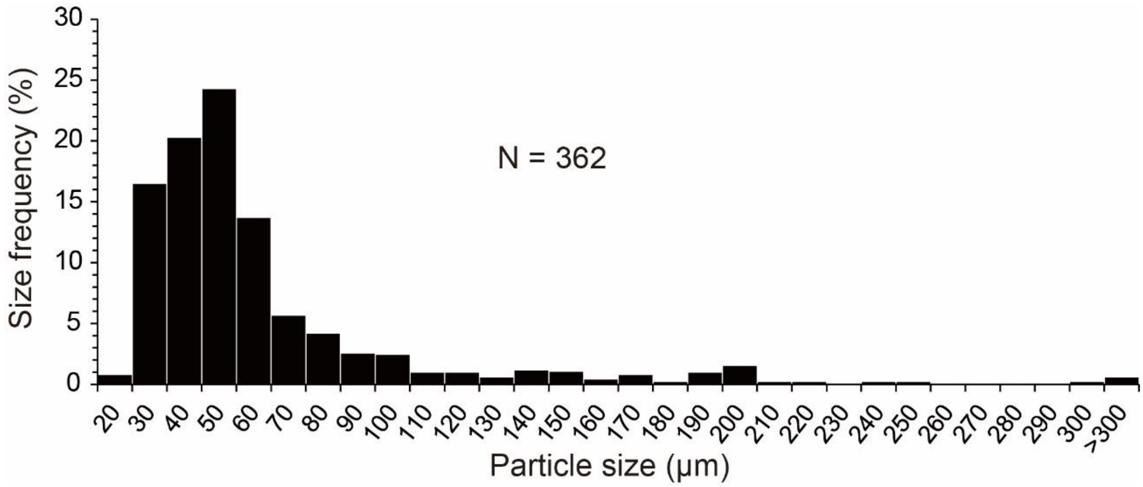

Figure S2. Relative microplastic particle size distribution at Stn. KEO.

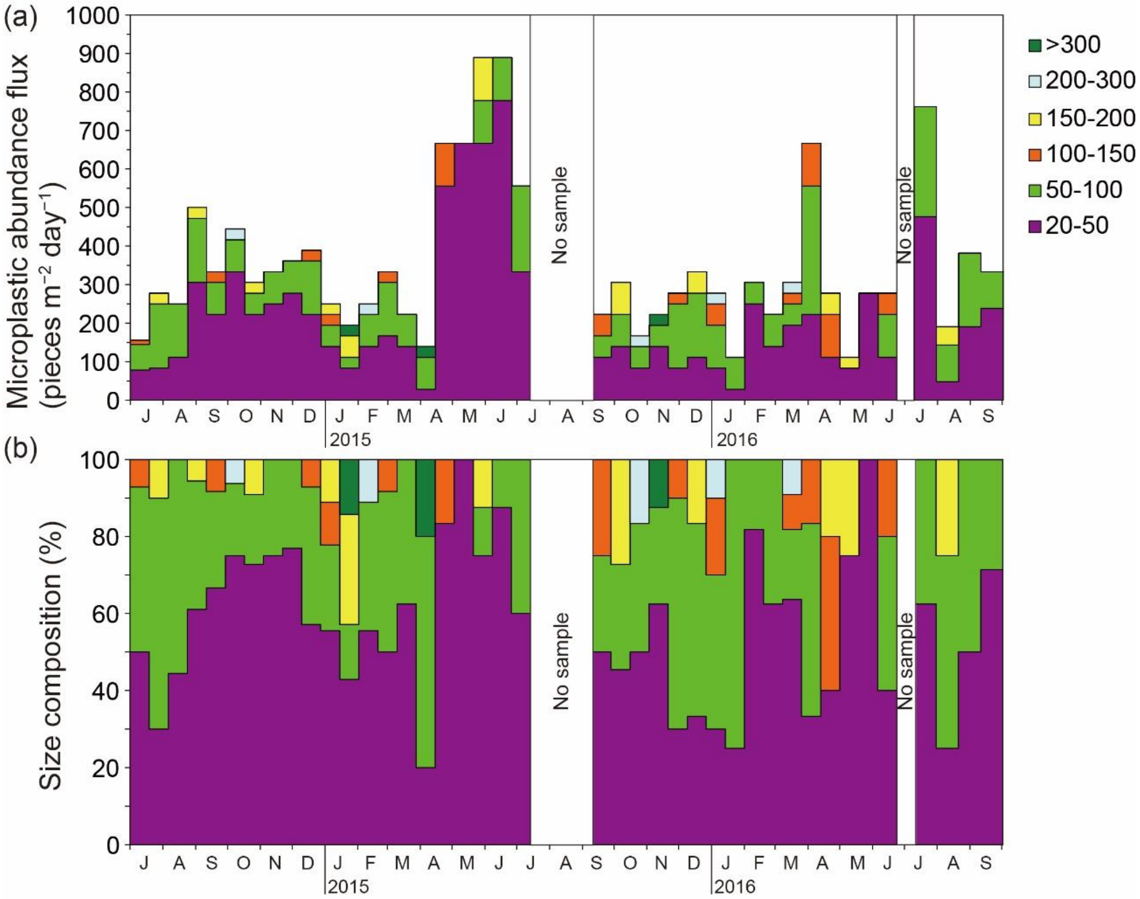

Figure S3. Time-series of microplastic abundance fluxes at Stn. KEO. (a) Microplastic abundance fluxes. (b) Size composition by number of particles.

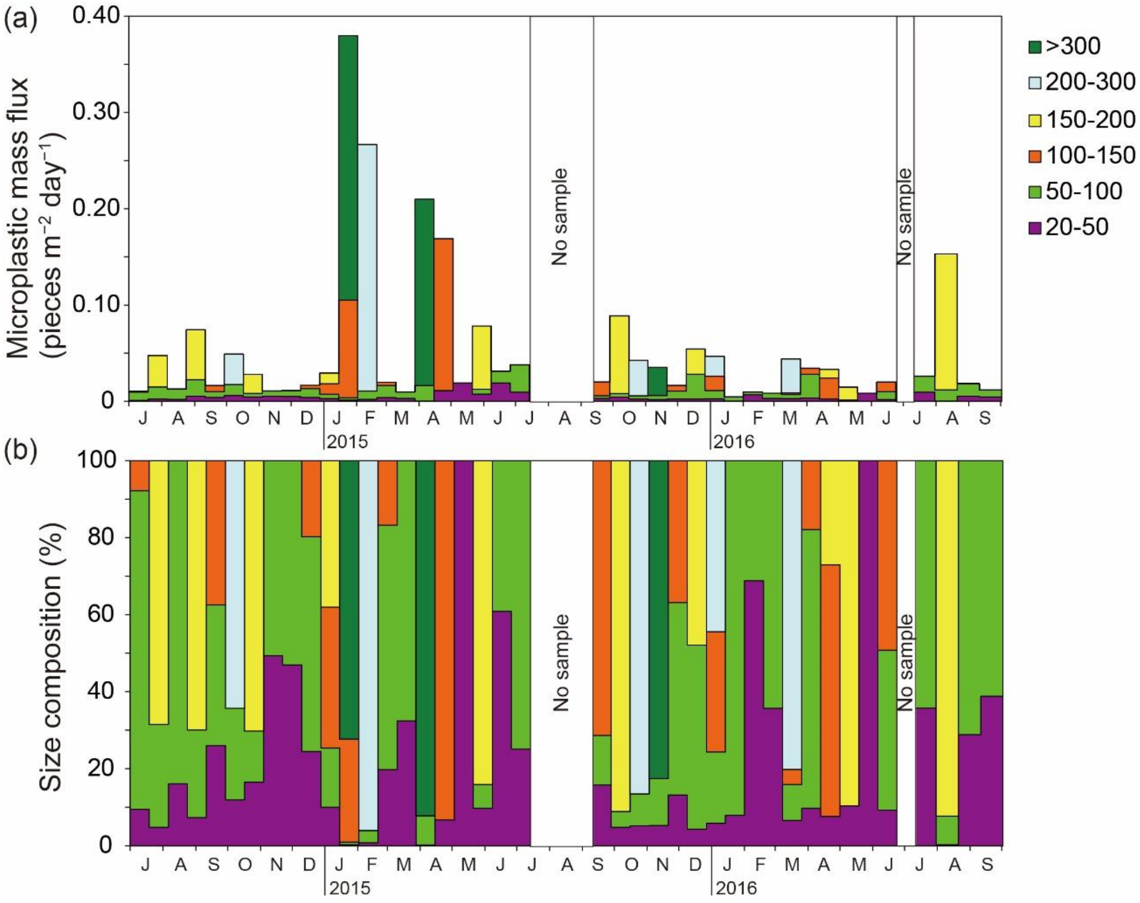

Figure S4. Time-series of microplastic mass fluxes at Stn. KEO. (a) Microplastic mass fluxes. (b) Size composition by mass.

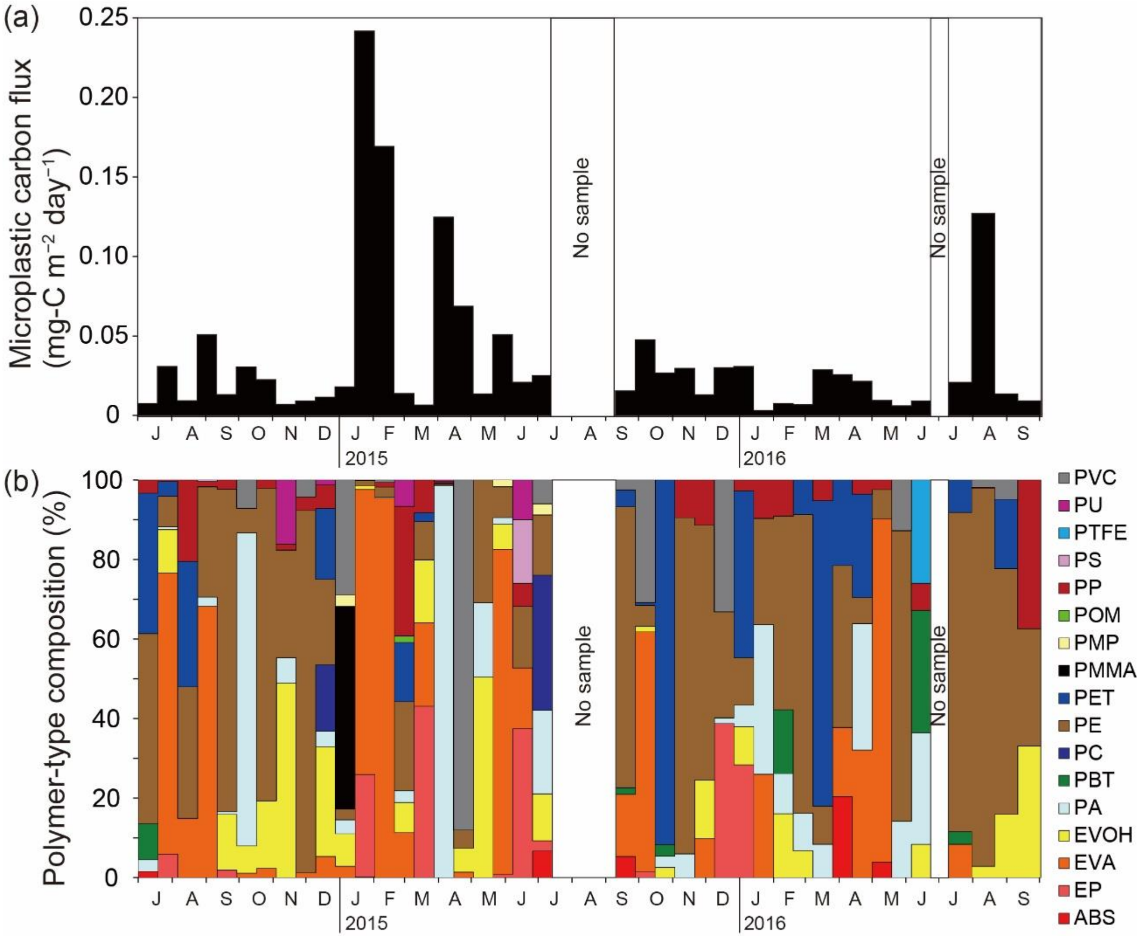

Figure S5. Time-series data of microplastic carbon fluxes at Stn. KEO. (a) Microplastic carbon fluxes. (b) Polymer-type composition by mass of carbon. Polymer-type abbreviations are as shown in Table S1.

SI References

(1) Behrenfeld, M. J.; Falkowski, P. G. Photosynthetic rates derived from satellite-based chlorophyll concentration. *Limnol. Oceanogr.* **1997** *42*(1), 1−20.

(2) Omnexus. Density of plastics: Technical properties, **2023**. https://omnexus.specialchem.com/polymer-properties/properties/density Accessed on August 31, 2023.

(3) Resin Library. What is Epoxy Resin, **2023**. https://www.resinlibrary.com/knowledge/article/epoxy-resin/ Accessed on August 31, 2023.

(4) Scientific Polymer Products Inc. Density of Polymers (By Density), **2019**. https://scipoly.com/density-of-polymers-by-density/ Accessed on January 15, 2024.

(5) Ji-Horng Plastic. Plastic Material: Density & Specific Gravity, **2023**. https://www.ji-horng.com/plastic-material-density-specific-gravity Accessed on August 31, 2023.

(6) Reineccius, J.; Waniek, J. J. First long-term evidence of microplastic pollution in the deep subtropical Northeast Atlantic. *Environ. Pollut.* **2022**, 305, 119302.

(7) Tsuchiya, M.; Kitahashi, T.; Nakajima, R.; Oguri, K.; Kawamura, K.; Nakamura, A.; Nakano, K.; Maeda, Y.; Murayama, M.; Chiba, S.; Fujikura, K. Distribution of microplastics in bathyal-to hadal-depth sediments and transport process along the deep-sea canyon and the Kuroshio Extension in the Northwest Pacific. *Mar. Pollut. Bull.* **2024**, *199*, 115466.

(8) Geyer, R.; Jambeck, J. R.; Law, K. L. Production, use, and fate of all plastics ever made. *Sci. Adv.* **2017**, *3* (7), e1700782.
